# Electrochemical Access to Aza‐Polycyclic Aromatic Hydrocarbons: Rhoda‐Electrocatalyzed Domino Alkyne Annulations

**DOI:** 10.1002/anie.201914775

**Published:** 2020-01-28

**Authors:** Wei‐Jun Kong, Zhigao Shen, Lars H. Finger, Lutz Ackermann

**Affiliations:** ^1^ Institut für Organische und Biomolekulare Chemie Georg-August-Universität Göttingen Tammannstraße 2 37077 Göttingen Germany

**Keywords:** aza-PAHs, C−H activation, domino reactions, metalla-electrocatalysis, rhodium catalysis

## Abstract

Nitrogen‐doped polycyclic aromatic hydrocarbons (aza‐PAHs) have found broad applications in material sciences. Herein, a modular electrochemical synthesis of aza‐PAHs was developed via a rhodium‐catalyzed cascade C−H activation and alkyne annulation. A multifunctional O‐methylamidoxime enabled the high chemo‐ and regioselectivity. The isolation of two key rhodacyclic intermediates made it possible to delineate the exact order of three C−H activation steps. In addition, the metalla‐electrocatalyzed multiple C−H transformation is characterized by unique functional group tolerance, including highly reactive iodo and azido groups.

## Graphical abstract

**Electrochemical access** to aza‐polycyclic aromatic hydrocarbons (aza‐PAHs) was achieved via rhoda‐electrocatalyzed domino C−H functionalization. A multifunctional *O*‐methylamidoxime facilitates the high chemo‐ and regioselectivity. The obtained aza‐PAHs and their derivatives have potential applications in catalysis, optoelectronics, and energy storage.

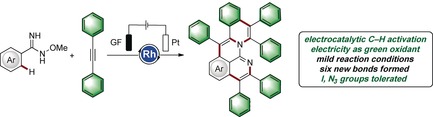

Polycyclic aromatic hydrocarbons (PAHs) are broadly explored in material sciences^1^ and synthetic chemistry.^2^ The incorporation of heteroatoms, such as nitrogen, into these conjugated π‐systems can tune or fundamentally alter their optoelectronic and catalytic properties.^3^ Therefore, the assembly of atomically precise aza‐PAHs in an efficient and economic manner has received considerable attention. However, the synthesis of PAHs and aza‐PAHs generally relies on stepwise elaborations, largely involving Diels–Alder cycloadditions, dehydrogenative cyclizations, and transition‐metal‐catalyzed cross‐couplings that require prefunctionalized substrates.^4^ Transition‐metal‐catalyzed oxidative C−H activation/annulation has been proven to be a powerful tool for PAH syntheses.^5^ However, the inherent sustainability of the C−H activation approach is compromised by the use of toxic and waste‐generating stoichiometric oxidants, such as copper(II) and silver(I) salts.

The merger of metal catalysis and electrosynthesis,^6^ metalla‐electrocatalysis,^7^ has been identified as a powerful strategy towards sustainable synthesis, which was substantiated by electrochemical metal‐catalyzed C−H functionalizations in recent years.^8^ Very recently, the groups led by Ackermann^9^ and Xu^10^ disclosed a new catalytic regime, namely anodic oxidation‐induced reductive elimination, where the electricity not only functions as the terminal oxidant, but is also responsible for anodic oxidation‐induced reductive elimination in rhodium‐catalyzed C−N and C−P bond formations. These findings may lead to new catalytic activities and transformations, which are not possible with common chemical oxidants.

While most of the aza‐PAHs synthesized by transition‐metal‐catalyzed C−H functionalization involve only one or two C−H activation steps, domino transformations in which three or more C−H bonds are activated and larger π‐extended systems constructed continue to be rare.^11^ To this end, we envisioned a multifunctional *O*‐methylamidoxime motif to facilitate threefold C−H activation by taking advantage of the imido group after the first internal oxidative C−H functionalization. With our ongoing interest in material syntheses by metalla‐electrocatalysis, we have now developed a one‐step electrochemical assembly of aza‐PAHs via rhoda‐electrocatalyzed cascade C−H annulations (Figure 1).^12^ Salient features of this electrocatalytic transformation include: a) electricity as a green oxidant, b) excellent functional group tolerance, c) three C−H bonds activated and six new bonds formed, and d) user‐friendly scale‐up.

**Figure 1:**
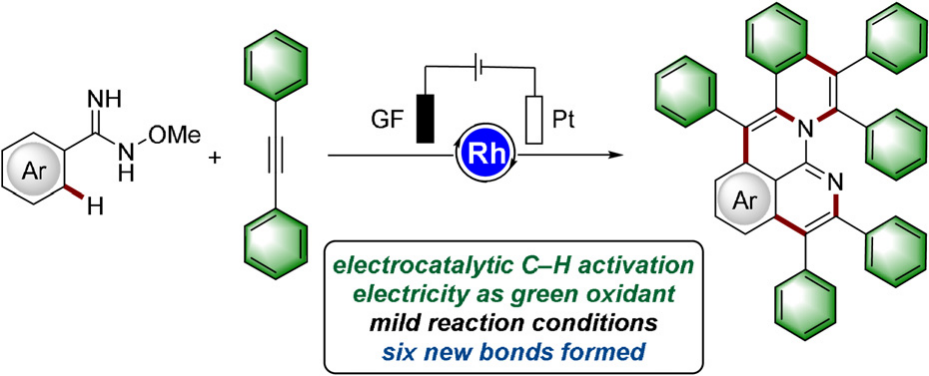
Electrochemical synthesis of aza‐nanographene via rhoda‐electrocatalyzed domino C−H annulations. GF: graphite felt.

We initiated our studies by using amidoxime **1 a** and diphenylacetylene (**2 a**) for the envisioned rhoda‐electrocatalyzed cascade C−H annulation (Table 1). When KOAc was used as the base and [Cp*RhCl_2_]_2_ (Cp*=C_5_Me_5_) as the catalyst precursor and with MeOH as the solvent and a constant current of 4.0 mA, the desired product **3 aa** was isolated in 38 % yield (entry 1). Other solvents, such as trifluoroethanol (TFE), H_2_O, and acetonitrile failed to deliver product **3 aa** (entries 2–5). Other bases showed inferior performance compared with KOAc (entries 5–8). The addition of catalytic amounts of carboxylic acids was found to be beneficial, with 1‐adamantanecarboxylic acid (AdOH) giving the best results (entries 9–11). The reaction gave a higher yield at a shorter reaction time, indicating that a high potential and prolonged electrolysis may lead to undesired side reactions or product decomposition (entry 12). Indeed, lowering the applied current to 2.0 mA increased the yield to 75 % with a prolonged reaction time (entry 13). Cationic rhodium catalyst [Cp*Rh(CH_3_CN)_3_](SbF_6_)_2_ further improved the efficacy, affording product **3 aa** in 90 % yield at 35 °C (entries 14 and 15). Control experiments verified the necessity of electricity and the rhodium catalyst (entries 16–18). Chemical oxidant Cu(OAc)_2_ failed to afford **3 aa** in effective yield (25 %).^13^ In addition, when nickel foam was used as the cathode or platinum as the anode, the reaction proceeded, albeit in inferior yields (67 % and 65 %, respectively).^13^

**Table 1:** Optimization of the rhoda‐electrocatalyzed domino C−H activation.^[a]^ 
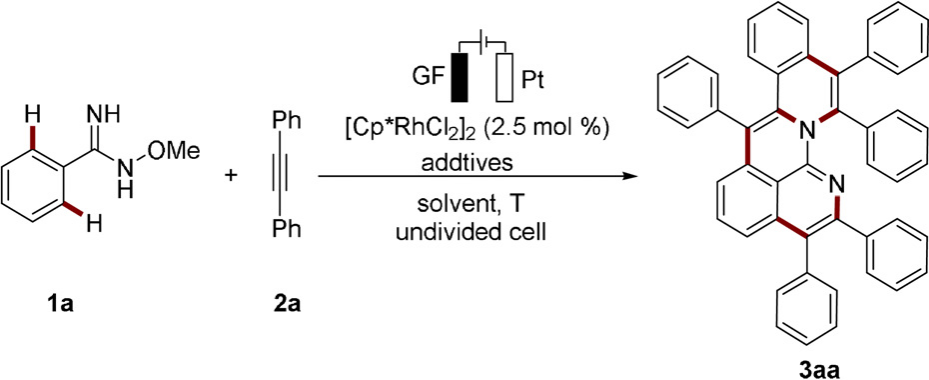

Entry	Base	Additive	Solv.	*I* [mA]	*t* [h]	**3 aa** [%]
1	KOAc	–	MeOH	4.0	10	38
2	KOAc	–	TFE	4.0	10	0
3	KOAc	–	H_2_O	4.0	10	0
4	KOAc	–	MeCN	4.0	10	0
5	KOAc	–	EtOH	4.0	10	4
6	NaOAc	–	MeOH	4.0	10	25
7	NaOPiv	–	MeOH	4.0	10	29
8	CsOAc	–	MeOH	4.0	10	9
9	KOAc	PivOH	MeOH	4.0	10	43
10	KOAc	AdOH	MeOH	4.0	10	46
11	KOAc	AcOH	MeOH	4.0	10	43
12	KOAc	AdOH	MeOH	4.0	6	56
13	KOAc	AdOH	MeOH	2.0	12	75
14	KOAc	AdOH	MeOH	2.0	12	90^[b]^
15	KOAc	AdOH	MeOH	2.0	12	89^[c]^
16	KOAc	AdOH	MeOH	–	12	trace^[d]^
17	KOAc	AdOH	MeOH	2.0	12	0^[e]^
18	KOAc	AdOH	MeOH	2.0	12	60^[f]^

[a] Undivided cell, graphite felt anode (GF) (10×15×6 mm^3^), platinum plate cathode (Pt) (10×15×0.25 mm^3^), **1 a** (0.2 mmol), **2 a** (0.7 mmol), [Cp*RhCl_2_]_2_ (2.5 mol %), base (2.0 equiv), additive (0.1 equiv), solvent (4.0 mL), 25 °C under air, yield of isolated product. [b] RhCp*(CH_3_CN)_3_(SbF_6_)_2_ (5.0 mol %), 35 °C. [c] RhCp*(CH_3_CN)_3_(SbF_6_)_2_ (2.5 mol %), 35 °C. [d] RhCp*(CH_3_CN)_3_(SbF_6_)_2_ (5.0 mol %), without electricity, 35 °C. [e] Without rhodium catalyst, 35 °C. [f] RhCp*(CH_3_CN)_3_(SbF_6_)_2_ (2.5 mol %), 35 °C, under N_2_.

After establishing the optimized reaction conditions for this rhoda‐electrocatalyzed cascade annulative C−H activation, we probed its versatility with various imidoximes **1** (Scheme 1). A broad range of aryl amidoximes **1** bearing electron‐donating (**1 b**, **1 c**, and **1 d**) and electron‐withdrawing groups (**1 e**–**1 h**) proved applicable for this electrocatalysis. For asymmetrically substituted substrates **1 i** and **1 j**, corresponding products were isolated with high levels of regioselectivity and good yields (**3 ia** and **3 ja**). Notably, the highly sensitive iodo substituent was well tolerated under the electrochemical conditions, which facilitates further postsynthetic modifications (**3 ka**).

**Scheme 1:**
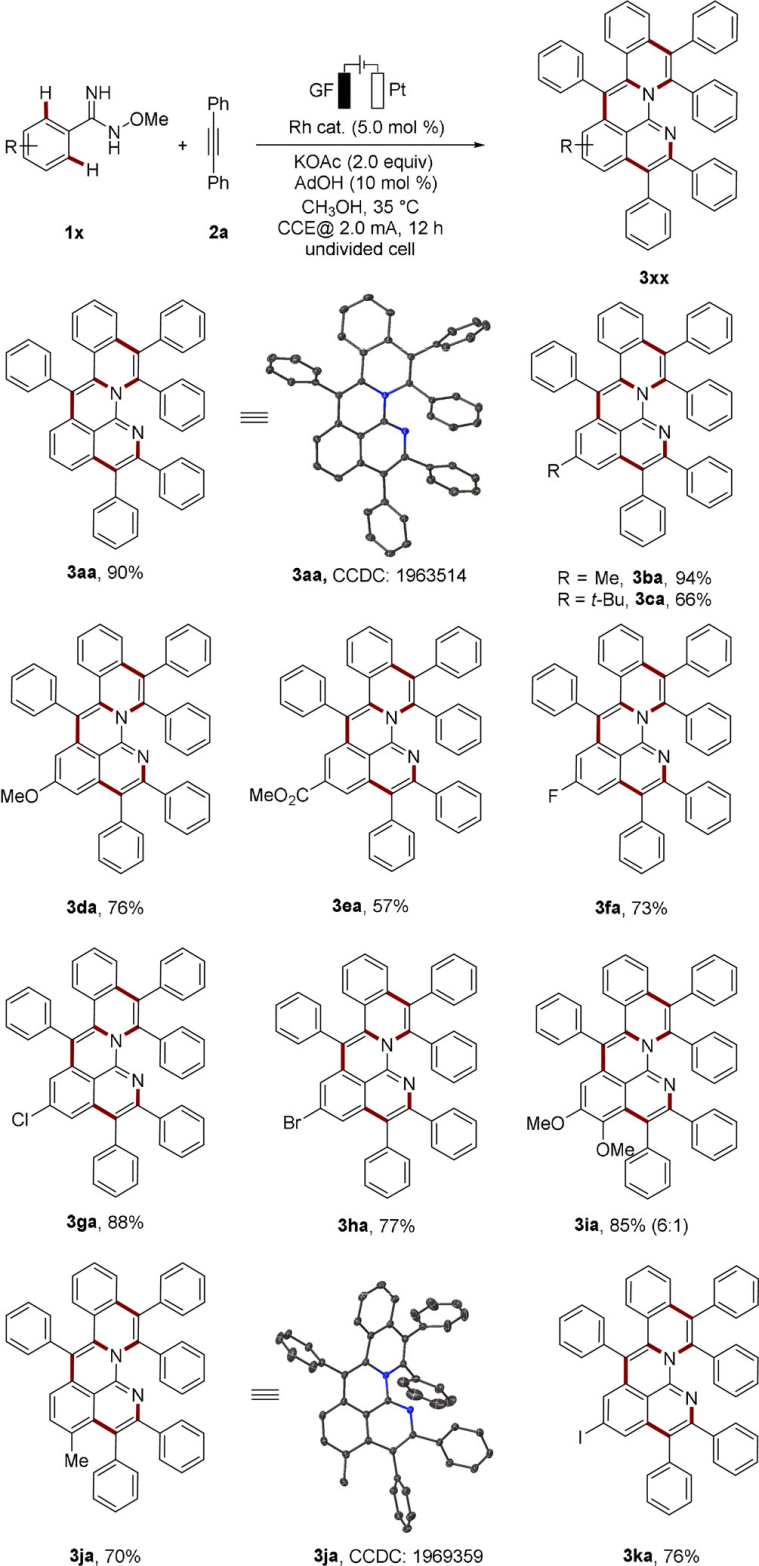
Rhoda‐electrocatalyzed C−H activation with amidoxime **1**. Rh cat.=[RhCp*(CH_3_CN)_3_](SbF_6_)_2_.

Subsequently, a variety of alkynes **2** were evaluated (Scheme 2). Alkynes **2** with electron‐donating substituents on the arene motif delivered the desired products (**3 ab**, **3 ac**, and **3 ad**). The trimethylsilyl group was well tolerated under the electrolysis conditions, serving as a handle for further transformations, such as Hiyama cross‐coupling.^14^ The cascade annulative reaction proceeded equally well with *meta*‐substituted alkyne **2 f**, affording the desired product **3 af** in high yield and selectivity. Remarkably, the challenging unsymmetrical alkyne **2 g** delivered the corresponding product with only two regioisomers and good selectivity. When alkyne **2 h** bearing pendant hydroxyl groups was applied, the micelle‐like PAH **3 ah** with hydrophilic hydroxyl groups surrounding the PAH moiety was obtained. Surprisingly, the highly functional azide group was well tolerated in this metalla‐electrocatalysis (**3 ai**), setting the stage for expedient postmodification and major potential in functional materials and bioimaging.

**Scheme 2:**
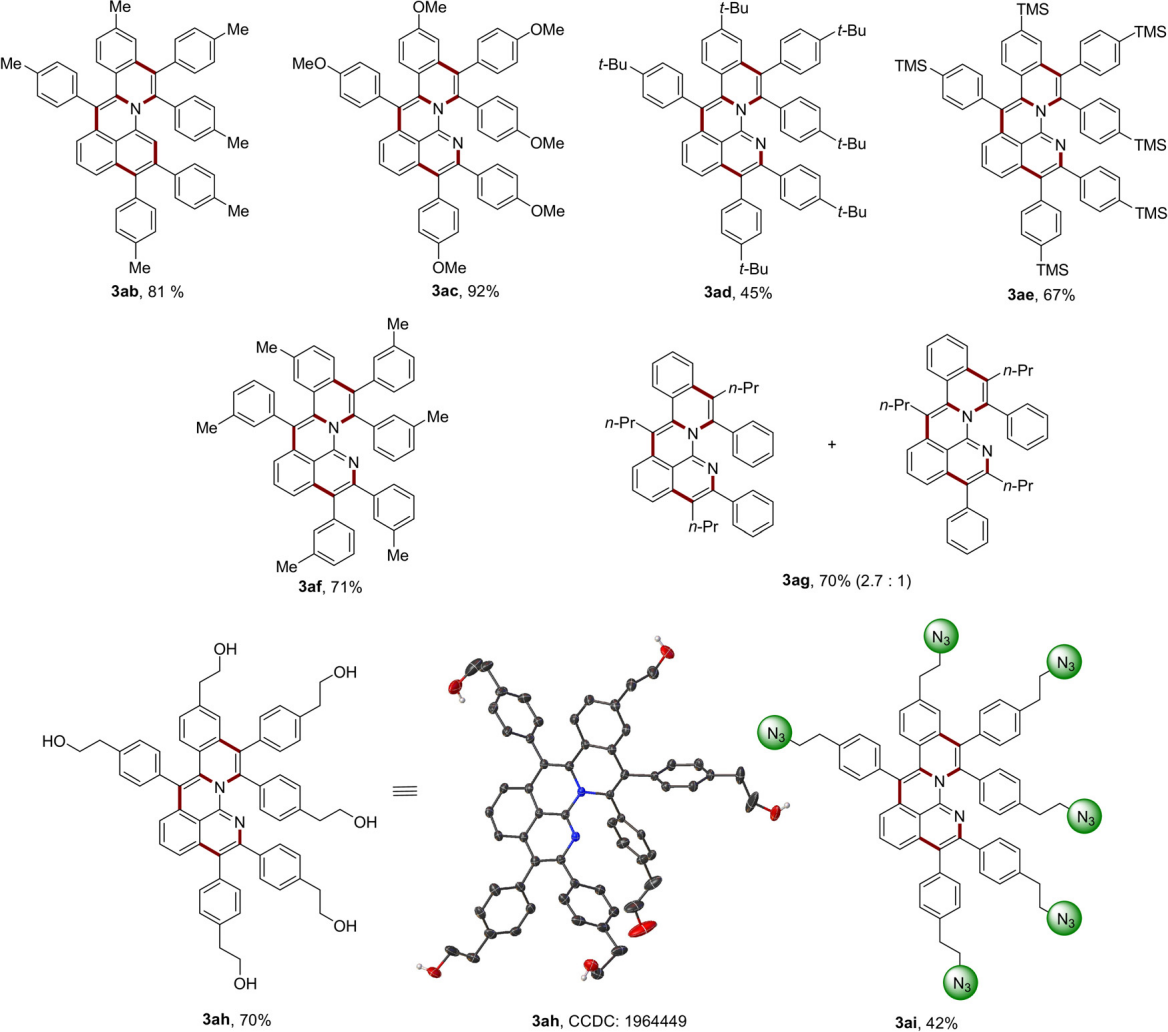
Rhoda‐electrocatalyzed C−H activation with alkyne **2**.

The high efficacy of this rhoda‐electrocatalyzed cascade C−H activation for the synthesis of aza‐PAHs motivated us to delineate its mode of action. To this end, intermediates **4** and **5** were isolated, which arise after the first and second C−H activation step, respectively (Scheme 3 a). Both rhodacycles **4** and **5** showed catalytic reactivity for the electrocatalysis (Scheme 3 b). This evidenced that the three C−H activation steps took place in the order of 1→2→3 (Scheme 3 c). As *N*‐methoxyamide was widely used in rhodium‐ and ruthenium‐catalyzed C−H annulations and functioned as an internal oxidant,^15^ a similar pathway might proceed for our new *O*‐methylamidoxime directing group, as N−O bond cleavage was observed in rhodacycle **5** and the products **3**. In addition, when cyclic voltammetry studies of **5** in the presence of alkyne **2 b** were conducted, a low oxidation peak at 0.23 V vs. ferrocene implied that an oxidatively induced reductive elimination pathway was involved for the second and third C−N bond formations (Figure 2). Based on these results, a detailed mechanism was proposed.^13^

**Figure 2:**
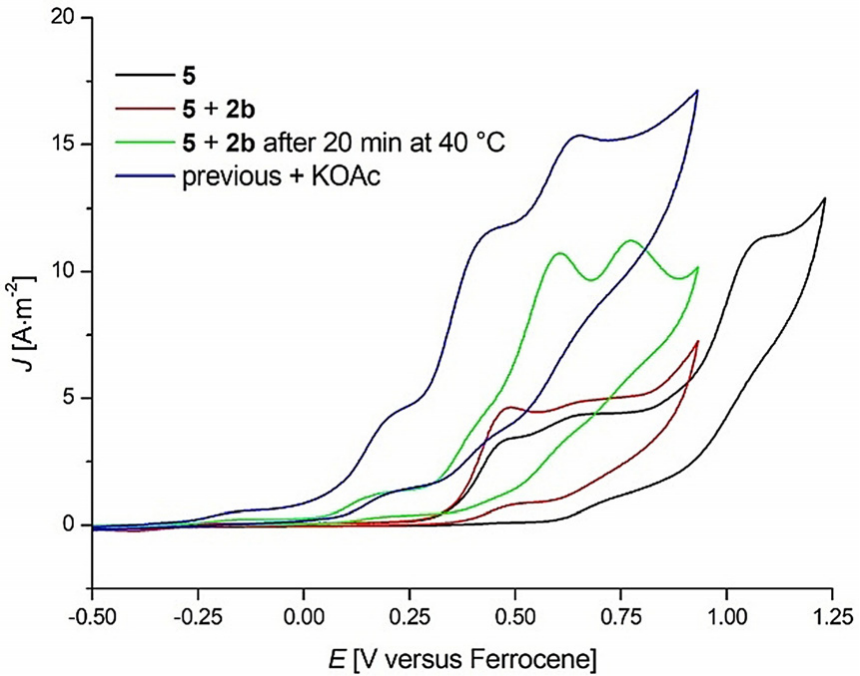
Cyclic voltammetry of complex **5** (2 mm, black), a mixture of **5** (2 mm) and **2 b** (10 mm, red), a mixture of **5** (2 mm) and **2 b** (10 mm) after heating to 40 °C for 20 minutes (green), and this mixture after addition of KOAc (10 mm, blue), all in methanol with *n*‐Bu_4_NPF_6_ (0.1 m) at 0.1 V s^−1^.

**Scheme 3:**
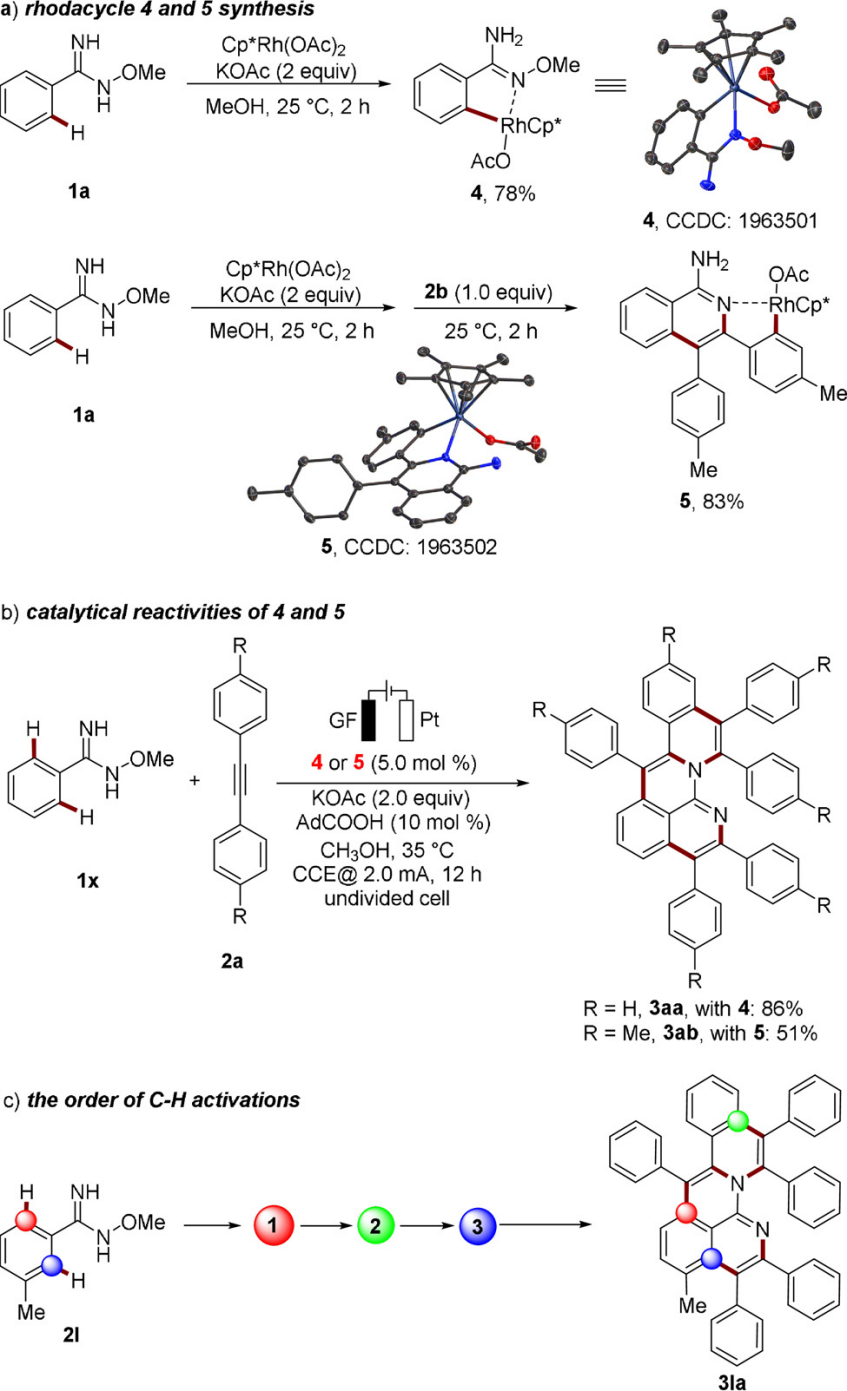
Mechanistic studies.

The obtained aza‐PAHs **3** could be easily transformed to further valuable functional molecular analogues. Treating aza‐PAH **3 aa** with iodomethane afforded a cationic nitrogen‐doped nanographene **6** in 93 % yield (Scheme 4), which showed reversible redox behavior with its radical form at a low potential of *E*_1/2_=−1.72 V vs. ferrocene (Figure 3). This promises applicability as a novel anolyte material in organic redox‐flow batteries.^16^ Intrigued by the six pendant azide groups on **3 aj**, we performed an azide–alkyne Huisgen cycloaddition reaction with a terminal alkyne linked to protected d‐lactone **7** (Scheme 5). Thus, a dendrimer with hydrophobic core and hydrophilic periphery **8** was assembled in high yield, which has potential applications in imaging and drug delivery.^17^

**Figure 3:**
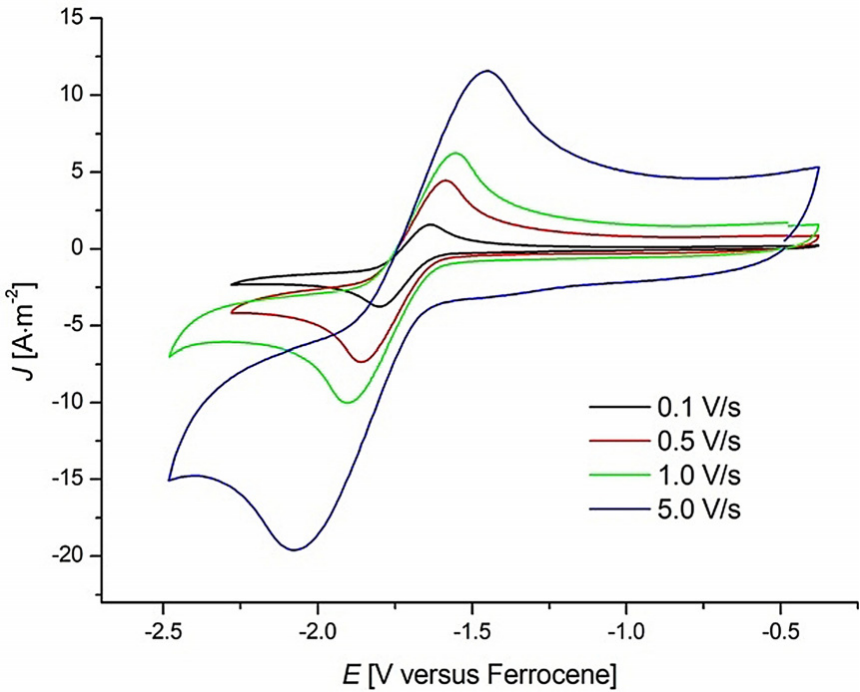
Cyclic voltammetry of compound **6** (2 mm) at varied scan rates, in dichloromethane with *n*‐Bu_4_NPF_6_ (0.1 m).

**Scheme 4:**
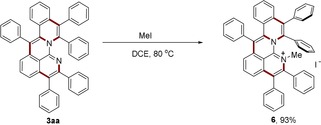
Synthesis of cationic aza‐nanographene **6**. DCE=1,2‐dichloroethane.

**Scheme 5:**
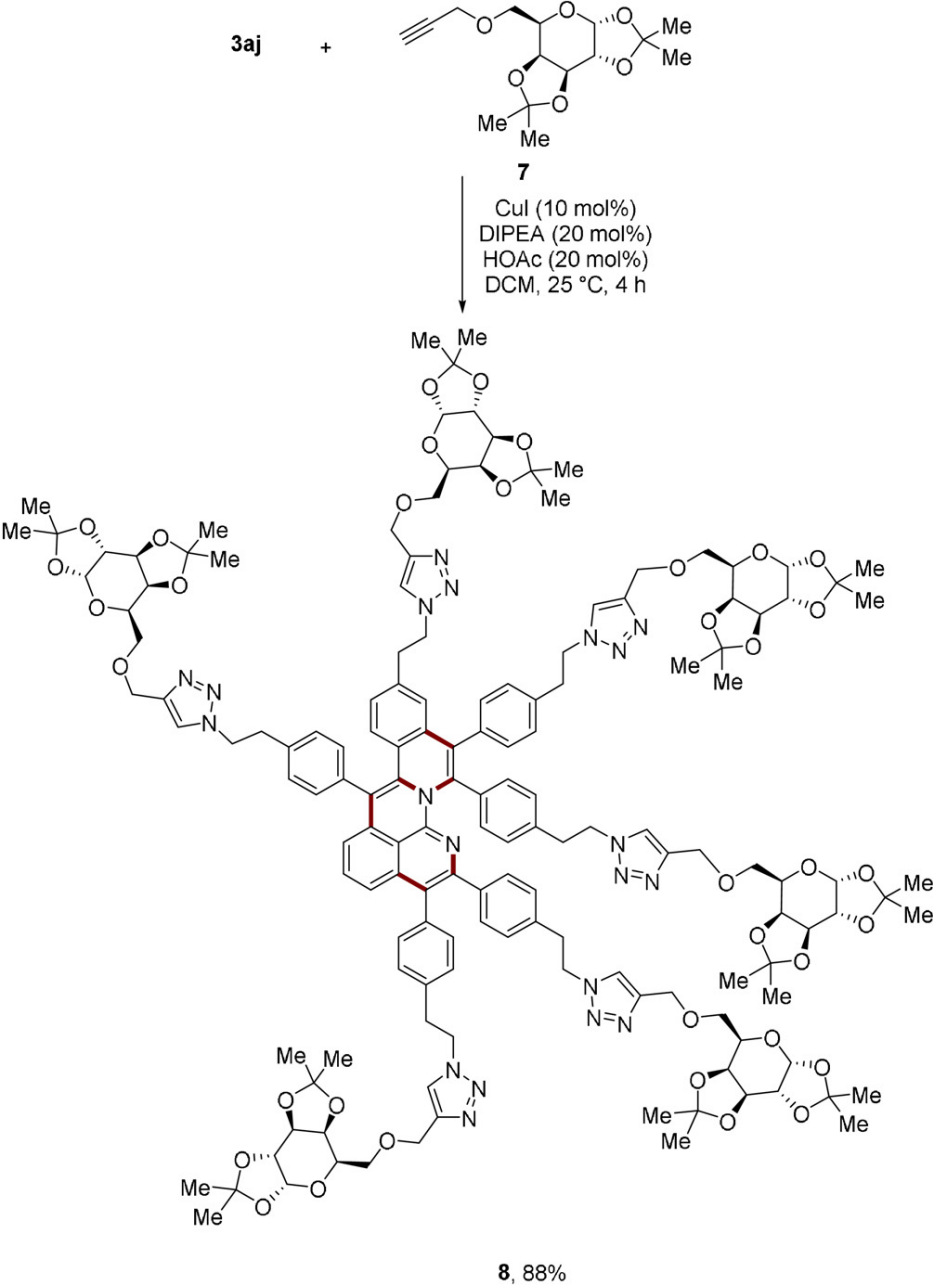
Synthesis of dendrimer **8** via sixfold aza‐alkyne addition between **3 aj** and **7**. DIPEA=*N*,*N*‐diisopropylethylamine.

The practicality of the rhoda‐electrocatalyzed cascade C−H activation for the synthesis of aza‐PAHs was further substantiated by its ease of scale up. Hence, the gram‐scale synthesis of product **3 aa** was realized with a reduced amount of the rhodium catalyst under a constant current of 12.0 mA (Scheme 6).

**Scheme 6:**
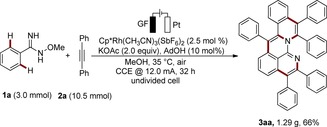
Gram‐scale synthesis of **3 aa**.

In summary, we have herein reported on a modular assembly of aza‐PAHs enabled by rhoda‐electrocatalyzed cascade C−H functionalization. A multifunctional and transformable *O*‐methylamidoxime was designed to guarantee the reactivity and selectivity. The isolation of two C−H‐activated rhodacycles revealed the order of the cascade C−H activation steps. The versatility of the electrosynthesis was demonstrated by its broad substrate scope and excellent functional group tolerance, including iodo and azido groups. The practicality of this reaction was reflected by its mild conditions, user‐friendly setup, and easy scale up. The obtained aza‐PAHs and their posttransformation derivatives were characterized in terms of their photophysical and electronic properties, which point to potential applications in optoelectronics, biomaterials, and energy storage.

## Conflict of interest

The authors declare no conflict of interest.

## Supporting information

As a service to our authors and readers, this journal provides supporting information supplied by the authors. Such materials are peer reviewed and may be re‐organized for online delivery, but are not copy‐edited or typeset. Technical support issues arising from supporting information (other than missing files) should be addressed to the authors.

Supplementary
