# Supplementary material for: Electrochemical Access to Aza‐Polycyclic Aromatic Hydrocarbons: Rhoda‐Electrocatalyzed Domino Alkyne Annulations
Source: Angew Chem Int Ed Engl. 2020 Jan 28;59(14):5551–6. doi: 10.1002/anie.201914775 (PMC7155118; doi:10.1002/anie.201914775)
Supplement: Supplementary file 1 — Supplementary [file ANIE-59-5551-s001.pdf]

Angewandte Chemie
Eine Zeitschrift der Gesellschaft Deutscher Chemiker

Supporting Information

Electrochemical Access to Aza-Polycyclic Aromatic Hydrocarbons: Rhoda-Electrocatalyzed Domino Alkyne Annulations

*Wei-Jun Kong+, Zhigao Shen+, Lars H. Finger, and Lutz Ackermann**

anie_201914775_sm_miscellaneous_information.pdf

Contents

1. General Remarks..S2

2. General Procedures for the Synthesis of Imidamides **1**..S3

3. Optimization of Rhodaelectro-Catalyzed Domino Alkyne Annulation...................S9

4. General Procedure for Rhodaelectro-Catalyzed Domino Alkyne Annulation S10

5. Mechanistic Studies S10

5.1. Rhodacycles **4** and **5** Synthesis..S10

5.2. Catalytic Reactivity of Rhodacycles **4** and **5**...S11

5.3 Cyclic Voltammetry...S12

6. UV-Vis Absorption and Emission Spectra of **3aa**..S17

7. Transformation of Product **3aa** and **3aj**..S18

8. X-Ray Crystallographic Analysis S21

9. Proposed Mechanism..S28

10. Characterization Data of Products S29

11. References..S47

12. NMR Spectra S48

1. General Remarks

The solvents methanol, dichloromethane, ethyl acetate, *n*-hexane and *n*-pentane were distilled prior to their use. Platinum electrodes (10 mm × 15 mm × 0.25 mm, 99.9%; obtained from ChemPur® Karlsruhe, Germany) and graphite felt electrodes (thickness of 6 mm, SIGRACELL® GFA 6 EA, obtained from SGL Carbon, Wiesbaden, Germany) were connected using stainless steel adapters. Electrocatalysis was conducted using a Keysight E36104A or an AXIOMET AX-3003P potentiostat. CV studies were performed using a Metrohm Autolab PGSTAT204 workstation and Nova 2.1 software. Yields refer to isolated compounds, estimated to be >95% pure as determined by ^{1}H NMR spectroscopy. Chromatography was carried out on Merck silica gel 60 (40–63 µm). NMR spectra were recorded on a Varian Mercury VX 300, Inova 500 or Bruker Avance III 300, Avance III 400 and Avance III HD 500 in the solvent indicated; chemical shifts (δ) are given in ppm relative to the residual solvent peak. All IR spectra were recorded on a Bruker FT-IR Alpha-P device. EI-MS was recorded on Jeol AccuTOF at 70eV, ESI-MS on Bruker MicrOTOF and maXis. GC-MS was recorded on Agilent 7890B and Agilent 5977B. M. p.: Stuart melting point apparatus SMP3, Barloworld Scientific, values are uncorrected. Fluorescence excitation and emission data in solution were recorded on a Jasco® FP-8500 spectrofluorometer.

2. General Procedures for the Synthesis of Imidamides

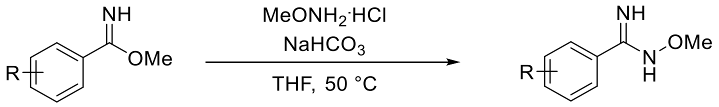

1-imidate **1**

Inspired by a literature procedure[1] we developed following general procedure **A** for the synthesis of **1**: To a 100 mL round flask was added **1-imidate** (1.0 equiv, 0.5 M in THF), methoxyammonium chloride (1.3 equiv), $NaHCO_3$ (1.3 equiv) and tetrahydrofuran. The reaction was stirred at 50 °C for 5 h. Removal of the solvent and subsequent column chromatography on silica gel afforded the corresponding *N*-methoxylimidamide **1**.

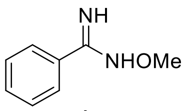

1a

N-Methoxybenzimidamide (1a)

The general procedure **A** was followed using methyl benzimidate (2.70 g, 20.0 mmol), $NaHCO_3$ (2.18 g, 26.0 mmol) and methoxyammonium chloride (2.17 g, 26.0 mmol) in THF (40 mL). Isolation by column chromatography (EtOAc/*n*-hexane: 1/5) yielded **1a** (2.61 g, 87%) as a white solid. M. p. = 59–60 °C. **^{1}H NMR** (400 MHz, $CDCl_3$) δ = 7.63 (dd, J = 7.5, 1.8 Hz, 2H), 7.44 – 7.33 (m, 3H), 4.80 (s_{br}, 2H), 3.92 (s, 3H). **^{13}C NMR** (101 MHz, $CDCl_3$) δ = 152.0 (C_q), 132.6 (C_q), 130.0 (CH), 128.7 (CH), 126.0 (CH), 61.6 (CH_3). **IR (ATR)**: 3440, 3317, 2936, 1631, 1567, 1399, 1046, 896, 520 cm^{-1}. **MS** (ESI) *m/z* (relative intensity): 151 (100) $[M+H]^+$, 173 (10) $[M+Na]^+$. **HR-MS** (ESI) *m/z* calcd for $C_8H_{11}N_2O$ $[M+H]^+$: 151.0866, found: 151.0868. The characterization data corresponds with those reported in the literature[2]

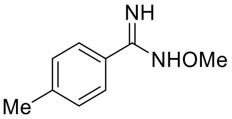

1b

N-Methoxy-4-methylbenzimidamide (1b)

The general procedure **A** was followed using methyl methyl 4-methylbenzimidate (0.70 g, 4.7 mmol), $NaHCO_3$ (0.51 g, 0.61 mmol) and methoxyammonium chloride (0.51 g, 0.61 mmol) in THF (9 mL). Isolation by column chromatography (EtOAc/*n*-hexane: 1/5) yielded **1b** (0.57 g, 74%) as a white solid. M. p. = 84–85 °C. **^{1}H NMR** (400 MHz, $CDCl_3$) δ = 7.52 (d, J = 7.8 Hz, 2H), 7.19 (d, J = 7.8 Hz, 2H), 4.78 (s_{br}, 2H), 3.91 (s, 3H), 2.36 (s, 3H). **^{13}C NMR** (100 MHz, $CDCl_3$) δ = 152.1 (C_q), 140.1 (C_q), 129.7 (C_q), 129.4 (CH), 125.9 (CH), 61.5 (CH_3), 21.5 (CH_3). **IR (ATR)**: 3453, 3306, 2955, 1625, 1398, 1045, 908, 820 cm^{-1}. **MS** (ESI) *m/z* (relative intensity): 165 (100) $[M+H]^+$, 187 (10) $[M+Na]^+$. **HR-MS** (ESI) *m/z* calcd for $C_9H_{13}N_2O$ $[M+H]^+$: 165.1022, found: 165.1026.

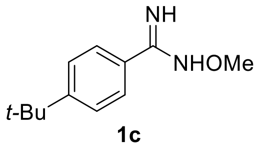

1c

4-(*t*ert-Butyl)-*N*-methoxybenzimidamide (1c)

The general procedure **A** was followed using methyl 4-(tert-butyl)benzimidate (1.22 g, 6.4 mmol), $NaHCO_3$ (0.70 g, 8.3 mmol) and methoxyammonium chloride (0.69 g, 8.3 mmol) in THF (13 mL). Isolation by column chromatography (EtOAc/*n*-hexane: 1/5) yielded **1c** (1.13 g, 86%) as a light yellow liquid. **^{1}H NMR** (300 MHz, $CDCl_3$) δ = 7.56 (d, J = 8.6 Hz, 2H), 7.40 (d, J = 8.6 Hz, 2H), 4.78 (s_{br}, 2H), 3.91 (s, 3H), 1.32 (s, 9H). **^{13}C NMR** (75 MHz, $CDCl_3$) δ = 153.3 (C_q), 152.0 (C_q), 129.8 (C_q), 125.7 (CH), 125.7 (CH), 61.5 (CH_3), 34.9 (C_q), 31.3 (CH_3). **IR (ATR)**: 3491, 3380, 2958, 1630, 1399, 1054, 900, 839 cm^{-1}. **MS** (ESI) *m/z* (relative intensity): 207 (100) $[M+H]^+$, 229 (20) $[M+Na]^+$. **HR-MS** (ESI) *m/z* calcd for $C_{12}H_{18}N_2O$ $[M+H]^+$: 207.1492, found: 207.1493.

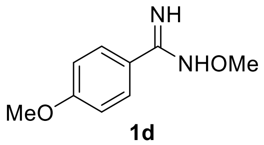

1d

***N*,4-Dimethoxybenzimidamide (1d)**

The general procedure **A** was followed using methyl 4-methoxybenzimidate (0.83 g, 5.0 mmol), $NaHCO_3$ (0.55 g, 6.5 mmol) and methoxyammonium chloride (0.54 g, 6.5 mmol) in THF (10 mL). Isolation by column chromatography (EtOAc/*n*-hexane: 1/5) yielded **1d** (0.74 g, 82%) as a white solid. M. p. = 92–93 °C. **^{1}H NMR** (400 MHz, $CDCl_3$) δ = 7.56 (d, J = 8.8 Hz, 2H), 6.90 (d, J = 8.8 Hz, 2H), 4.76 (s_{br}, 2H), 3.90 (s, 3H), 3.82 (s, 3H). **^{13}C NMR** (101 MHz, $CDCl_3$) δ = 161.1 (C_q), 151.9 (C_q), 127.4 (CH), 125.0 (C_q), 114.1 (CH), 61.5 (CH_3), 55.5 (CH_3). **IR (ATR)**: 3450, 3307, 2953, 1613, 1516, 1397, 1241, 1027, 838 cm^{-1}. **MS** (ESI) *m/z* (relative intensity): 181 (100) $[M+H]^+$, 203 (10) $[M+Na]^+$. **HR-MS** (ESI) *m/z* calcd for $C_9H_{13}N_2O_2$ $[M+H]^+$: 181.0972, found: 181.0972.

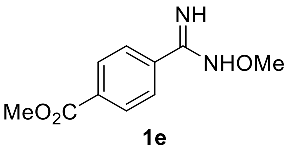

1e

Methyl 4-(*N*-methoxycarbamimidoyl)benzoate (1e)

The general procedure **A** was followed using methyl 4-(imino(methoxy)methyl) benzoate (0.40 g, 2.1 mmol), $NaHCO_3$ (0.23 g, 2.7 mmol) and methoxyammonium chloride (0.22 g, 2.7 mmol) in THF (5 mL). Isolation by column chromatography (Et_2O/*n*-pentane: 1/5) yielded **1e** (0.39 g, 89%) as a white solid. M. p. = 94–95 °C. **^{1}H NMR** (300 MHz, $CDCl_3$) δ = 8.04 (d, J = 8.2 Hz, 2H), 7.70 (d, J = 8.2 Hz, 2H), 4.83 (s_{br}, 2H), 3.93 (s, 3H), 3.92 (s, 3H). **^{13}C NMR** (75 MHz, $CDCl_3$) δ = 166.7 (C_q), 150.9 (C_q), 136.7 (C_q), 131.4 (C_q), 130.0 (CH), 125.9 (CH), 61.8 (CH_3), 52.4 (CH_3). **IR (ATR)**: 3449, 3348, 2948, 1698, 1640, 1399, 1283, 1040, 898 cm^{-1}. **MS** (ESI) *m/z* (relative intensity): 209 (100) $[M+H]^+$. **HR-MS** (ESI) *m/z* calcd for $C_{10}H_{13}N_2O_3$ $[M+H]^+$: 209.0921, found: 209.0928.

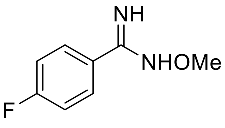

1f

4-Fluoro-*N*-methoxybenzimidamide (1f)

The general procedure **A** was followed using methyl 4-fluorobenzimidate (0.77 g, 5.0 mmol), $NaHCO_3$ (0.55 g, 6.5 mmol) and methoxyammonium chloride (0.54 g, 6.5 mmol) in THF (10 mL). Isolation by column chromatography (EtOAc/*n*-hexane: 1/5) yielded **1f** (0.72 g, 86%) as a white solid. M. p. = 74–75 °C. **^{1}H NMR** (300 MHz, $CDCl_3$) δ = 7.61 (td, J = 8.7, 5.4 Hz, 2H), 7.06 (t, J = 8.7 Hz, 2H), 4.77 (s_{br}, 2H), 3.90 (s, 3H). **^{13}C NMR** (75 MHz, $CDCl_3$) δ = 163.9 (C_q, $^1J_{C–F}$ = 249.4 Hz), 151.2 (C_q), 128.8 (C_q, ^4J = 3.3 Hz), 128.0 (CH, $^3J_{C–F}$ = 8.4 Hz), 115.8 (CH, $^2J_{C–F}$ = 21.8 Hz), 61.6 (CH_3). **^{19}F NMR** (282 MHz, $CDCl_{3)}$ δ =-110.90—110.99 (m). **IR (ATR)**: 3453, 3308, 2953, 1604, 1513, 1400, 1228, 1048, 837 cm^{-1}. **MS** (ESI) *m/z* (relative intensity): 169 (100) $[M+H]^+$. **HR-MS** (ESI) *m/z* calcd for $C_8H_{10}FN_2O$ $[M+H]^+$: 169.0772, found: 169.0773.

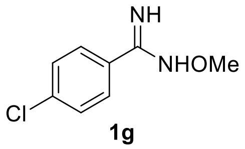

1g

4-Chloro-*N*-methoxybenzimidamide (1g)

The general procedure **A** was followed using methyl 4-chlorobenzimidate (0.85 g, 5.0 mmol), $NaHCO_3$ (0.55 g, 6.5 mmol) and methoxyammonium chloride (0.54 g, 6.5 mmol) in THF (10 mL). Isolation by column chromatography (EtOAc/*n*-hexane: 1/5) yielded **1g** (0.63 g, 68%) as a white solid. M. p. = 97–98 °C. **^{1}H NMR** (400 MHz, $CDCl_3$) δ = 7.58 (d, J = 8.5 Hz, 2H), 7.36 (d, J = 8.5 Hz, 2H), 4.77 (s_{br}, 2H), 3.91 (s, 3H). **^{13}C NMR** (101 MHz, $CDCl_3$) δ = 150.9 (C_q), 136.0 (C_q), 131.1 (C_q), 129.0 (CH), 127.3 (CH), 61.7 (CH_3). **IR (ATR)**: 3451, 3301, 2956, 1627, 1398, 1047, 907, 821 cm^{-1}. **MS** (ESI) *m/z* (relative intensity): 185 (100) $[M+H]^+$, 153 (10). **HR-MS** (ESI) *m/z* calcd for $C_8H_{10}ClN_2O$ $[M+H]^+$: 185.0476, found: 185.0476.

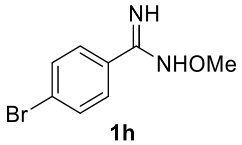

1h

4-Bromo-*N*-methoxybenzimidamide (1h)

The general procedure **A** was followed using methyl 4-bromobenzimidate (1.16 g, 5.5 mmol), $NaHCO_3$ (0.60 g, 7.1 mmol) and methoxyammonium chloride (0.60 g, 7.1 mmol) in THF (10 mL). Isolation by column chromatography (EtOAc/*n*-hexane: 1/5) yielded **1h** (0.54 g, 43%) as a white solid. M. p. = 111–112 °C. **^{1}H NMR** (300 MHz, $CDCl_3$) δ = 7.53 (s, 4H), 4.79 (s_{br}, 2H), 3.93 (s, 3H). **^{13}C NMR** (75 MHz, $CDCl_3$) δ = 151.0 (C_q), 131.9 (CH), 131.5 (C_q), 127.5 (CH), 124.2 (C_q), 61.7 (CH_3). **IR (ATR)**: 3450, 3302, 2955, 1628, 1399, 1047, 908, 822 cm^{-1}. **MS** (ESI) *m/z* (relative intensity): 229 (100) $[M+H]^+$, 251 (10). **HR-MS** (ESI) *m/z* calcd for $C_8H_{10}{}^{79}BrN_2O$ $[M+H]^+$: 228.9971, found: 228.9974.

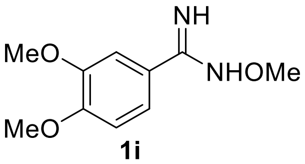

1i

_N_,3,4-Trimethoxybenzimidamide (1i)

The general procedure **A** was followed using methyl 3,4-dimethoxybenzimidate (2.10 g, 10.8 mmol), $NaHCO_3$ (1.17 g, 14.0 mmol) and methoxyammonium chloride (1.18 g, 14.0 mmol) in THF (20 mL). Isolation by column chromatography (Et_2O/*n*-pentane: 1/5) yielded **1i** (1.76 g, 76%) as a white solid. M. p. = 92–93 °C. **^{1}H NMR** (300 MHz, $CDCl_3$) δ = 7.19 (d, J = 2.0 Hz, 1H), 7.12 (dd, J = 8.3, 2.0 Hz, 1H), 6.83 (d, J = 8.3 Hz, 1H), 4.78 (s_{br}, 2H), 3.89 (s, 6H), 3.87 (s, 3H). **^{13}C NMR** (75 MHz, $CDCl_3$) δ = 152.0 (C_q), 150.6 (C_q), 149.0 (C_q), 125.3 (C_q), 118.5 (CH), 110.9 (CH), 109.2 (CH), 61.4 (CH_3), 56.0 (CH_3), 56.0 (CH_3). **IR (ATR)**: 3463, 3331, 2944, 1630, 1419, 1237, 1017, 869, 759 cm^{-1}. **MS** (ESI) *m/z* (relative intensity): 211 (100) $[M+H]^+$, 233 (20) $[M+Na]^+$. **HR-MS** (ESI) *m/z* calcd for $C_{10}H_{15}N_2O_3$ $[M+H]^+$: 211.1077, found: 211.1080.

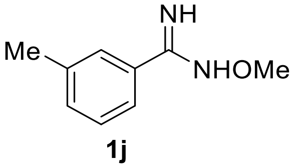

1j

***N*-Methoxy-3-methylbenzimidamide (1j)**

The general procedure **A** was followed using methyl 3-methylbenzimidate (0.81 g, 5.4 mmol), $NaHCO_3$ (0.59 g, 7.0 mmol) and methoxyammonium chloride (0.59 g, 7.0 mmol) in THF (20 mL). Isolation by column chromatography (EtOAc/*n*-hexane: 1/5) yielded **1j** (0.69 g, 78%) as a white solid. M. p. = 57–58 °C. **^{1}H NMR** (300 MHz, $CDCl_3$) δ = 7.49 (s, 1H), 7.43 (d, J = 7.5 Hz, 1H), 7.30 (t, J = 7.5 Hz, 1H), 7.24 (d, J = 7.5 Hz, 1H), 4.81 (s_{br}, 2H), 3.94 (s, 3H), 2.40 (s, 3H). **^{13}C NMR** (75 MHz, $CDCl_3$) δ = 152.2 (C_q), 138.5 (C_q), 132.5 (C_q), 130.8 (CH), 128.7 (CH), 126.7 (CH), 123.0 (CH), 61.6 (CH_3), 21.5 (CH_3). **IR (ATR)**: 3452, 2906, 1629, 1572, 1386, 1046, 912, 478 cm^{-1}. **MS** (ESI) *m/z* (relative intensity): 165 (100) $[M+H]^+$, 187 (10) $[M+Na]^+$. **HR-MS** (ESI) *m/z* calcd for $C_9H_{13}N_2O$ $[M+H]^+$: 165.1022, found: 165.1028.

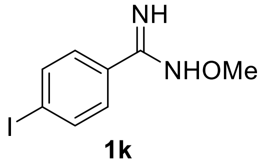

1k

4-Iodo-*N*-methoxybenzimidamide (1k)

The general procedure **A** was followed using methyl 4-iodobenzimidate (1.04 g, 4.0 mmol), $NaHCO_3$ (0.44 g, 5.2 mmol) and methoxyammonium chloride (0.43 g, 5.2 mmol) in THF (8 mL). Isolation by column chromatography (EtOAc/*n*-hexane: 1/5) yielded **1k** (0.76 g, 69%) as a white solid. M. p. = 125–126 °C. **^{1}H NMR** (400 MHz, $CDCl_3$) δ = 7.72 (d, J = 8.4 Hz, 2H), 7.36 (d, J = 8.4 Hz, 2H), 4.76 (s_{br}, 2H), 3.91 (s, 3H). **^{13}C NMR** (101 MHz, $CDCl_3$) δ = 151.0 (C_q), 137.9 (CH), 132.1 (C_q), 127.6 (CH), 96.1 (C_q), 61.7 (CH_3). **IR (ATR)**: 3450, 3303, 2953, 1627, 1396, 1045, 906, 812 cm^{-1}. **MS** (ESI) *m/z* (relative intensity): 277 (100) $[M+H]^+$, 227 (10). **HR-MS** (ESI) *m/z* calcd for $C_8H_{10}IN_2O$ $[M+H]^+$: 276.9832, found: 276.9833.

3. Optimization of Rhodaelectro-Catalyzed Domino Alkyne Annulation

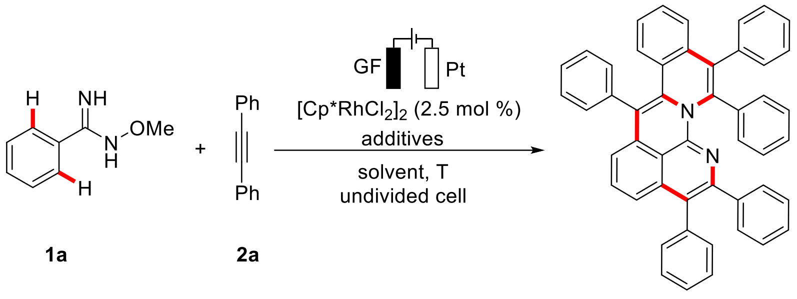

Entry	Base	Additive	Solvent	I [mA]	Time [h]	3aa [%]
1	KOAc	-	MeOH	4.0	10	38
2	KOAc	-	TFE	4.0	10	0
3	KOAc	-	H_2O	4.0	10	0
4	KOAc	-	MeCN	4.0	10	0
5	KOAc	-	EtOH	4.0	10	4
6	NaOAc	-	MeOH	4.0	10	25
7	NaOPiv	-	MeOH	4.0	10	29
8	CsOAc	-	MeOH	4.0	10	9
9	KOAc	PivOH	MeOH	4.0	10	43
10	KOAc	AdOH	MeOH	4.0	10	46
11	KOAc	AcOH	MeOH	4.0	10	43
12	KOAc	AdOH	MeOH	4.0	6	56
13	KOAc	AdOH	MeOH	2.0	12	75
14	KOAc	AdOH	MeOH	2.0	12	90[b]
15	KOAc	AdOH	MeOH	2.0	12	89[c]
16	KOAc	AdOH	MeOH	_	12	trace[d]
17	KOAc	AdOH	MeOH	2.0	12	0[e]
18	KOAc	AdOH	MeOH	2.0	12	60[f]
19	KOAc	AdOH	MeOH	2.0	12	25[g]
20	KOAc	AdOH	MeOH	2.0	12	67[h]
21	KOAc	AdOH	MeOH	2.0	12	65[i]

[a] Undivided cell, graphite felt anode (GF), platinum plate cathode (Pt), **1a** (0.2 mmol), **2a** (0.7 mmol), $[Cp^*RhCl_2]_2$ (2.5 mol %), base (2.0 equiv), additive (0.1 equiv), solvent (4.0 mL), 25 °C under air, isolated yield. [b] $[Cp^*Rh(CH_3CN)_3](SbF_6)_2$ (5.0 mol %), 35 °C. [c] $[Cp^*Rh(CH_3CN)_3](SbF_6)_2$ (2.5 mol %), 35 °C. [d] $[Cp^*Rh(CH_3CN)_3](SbF_6)_2$ (5.0 mol %), without electricity, 35 °C. [e] Without rhodium catalyst, 35 °C. [f] $[Cp^*Rh(CH_3CN)_3](SbF_6)_2$ (2.5 mol %), 35 °C, under N_2. [g] $[Cp^*Rh(CH_3CN)_3](SbF_6)_2$ (5.0 mol %), 35 °C, with $Cu(OAc)_2$ (4.0 equiv). [h] $[Cp^*Rh(CH_3CN)_3](SbF_6)_2$ (5.0 mol %), 35 °C, platinum plate was used as anode [i] $[Cp^*Rh(CH_3CN)_3](SbF_6)_2$ (5.0 mol %), 35 °C, nickel foam was used as cathode.

4. General Procedure for Rhodaelectro-Catalyzed Domino Alkyne Annulation

The electrocatalysis was carried out in an undivided cell with a graphite felt anode (10 mm × 15 mm × 6 mm) and a Pt cathode (10 mm × 15 mm × 0.25 mm). The cell was charged with imidamide **1** (0.2 mmol), alkyne **2** (0.7 mmol), KOAc (74.4 mg, 0.4 mmol), 1-adamantanecarboxylic acid (AdOH, 3.6 mg, 10 mol %), $[Cp^*Rh(CH_3CN)_3](SbF_6)_2$ (8.4 mg, 5.0 mol %) and MeOH (4.0 mL). Electrocatalysis was performed at 35 °C with a constant current of 2.0 mA maintained for 12 h. The graphite felt anode was washed with CH_2Cl_2 (8 × 5 mL) in an ultrasonic bath. Evaporation of the solvents and subsequent column chromatography on silica gel afforded the corresponding product **3**.

5. Mechanistic Studies

5.1. Rhodacycles 4 and 5 Synthesis

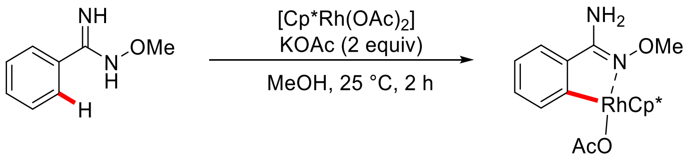

1a **4**

A 15 mL Schlenk tube was charged with $[Cp^*Rh(OAc)_2]$ (21.4 mg, 0.06 mmol), KOAc (12.0 mg, 0.12 mmol), and MeOH (2.0 mL). The mixture was stirred at 25 °C for 2 h. After removal of the solvent by vacuum, the mixture was extracted with CH_2Cl_2 (4.0 mL) and filtered with a syringe filter. After removal of CH_2Cl_2, crystallization (*n*-pentane and CH_2Cl_2) afforded rhodacycle **4** as a red solid (20.8 mg, 78%). M. p. = 189–189 °C. **^{1}H NMR** (300 MHz, CD_3OD) δ = 7.92 (d, J = 7.5 Hz, 1H), 7.54 (d, J = 7.7 Hz, 1H), 7.34 (t, J = 7.3 Hz, 1H), 7.16 (t, J = 7.3 Hz, 1H), 5.51 (s, 2H), 3.79 (s, 3H), 1.89 (s, 3H), 1.68 (s, 15H). **^{13}C NMR** (100 MHz, CD_3OD) δ = 178.7 (C_q), 173.1 (C_q), 167.1 (C_q), 136.8 (C_q), 135.7 (CH), 130.8 (CH), 128.0 (CH), 123.5 (CH), 95.3 (d, $^1J_{C\text{-}Rh}$ = 6.0 Hz, C_q), 59.6 (CH_3), 22.8 (CH_3), 8.0 (CH_3). **IR** (ATR): 3150, 3128, 2922, 1645, 1574, 1369, 1013, 727, 662 cm^{-1}. **HR-MS** (ESI) *m/z* calcd for $RhC_{18}H_{24}N_2O$ $[M\text{-}OAc]^+$: 387.0938, found: 387.0939.

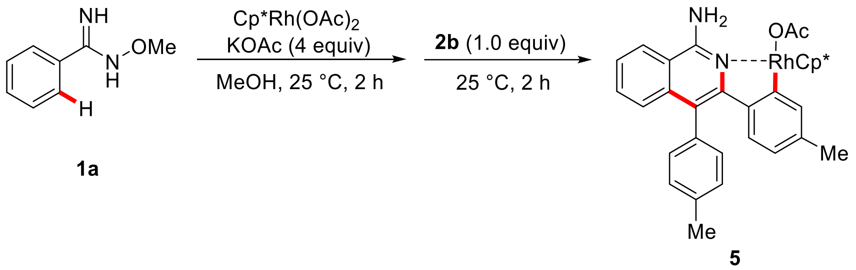

A 15 mL Schlenk tube was charged with $Cp^*Rh(OAc)_2$ (42.7 mg, 0.12 mmol), KOAc (47.0 mg, 0.48 mmol), and MeOH (2.0 mL). The mixture was stirred at 25 °C for 2 h. Then alkyne **2b** (24.7 mg, 0.12 mmol) was added to the mixture, which was stirred at 25 °C for another 2 h. After removal of the solvent by vacuum, the mixture was extracted with CH_2Cl_2 (4.0 mL) and filtered with a syringe filter. After removal of CH_2Cl_2, crystallization (*n*-pentane and CH_2Cl_2) afforded rhodacycle **5** as a red solid (62.0 mg, 83%). Decomposed at 285 °C. **^{1}H NMR** (400 MHz, $CDCl_3$) δ = 8.54 (s_{br}, 2H), 8.02 (d, J = 8.1 Hz, 1H), 7.70 (s, 1H), 7.55 – 7.36 (m, 4H), 7.34 – 7.22 (m, 2H), 7.12 (d, J = 7.6 Hz, 1H), 6.52 – 6.45 (m, 1H), 6.26 (d, J = 8.1 Hz, 1H), 2.51 (s, 3H), 2.34 (s, 3H), 2.08 (s, 3H), 1.48 (s, 15H). **^{13}C NMR** (100 MHz, $CDCl_3$) δ = 180.4 (d, $^2J_{C\text{-}Rh}$ = 1.3 Hz, C_q), 180.1 (d, $^2J_{C\text{-}Rh}$ = 29.2 Hz, C_q), 159.2 (C_q), 152.7 (C_q), 143.9 (C_q), 137.8 (C_q), 137.2 (C_q), 137.2 (C_q), 135.8 (CH), 135.1 (C_q), 131.5 (CH), 130.9 (CH), 130.6 (CH), 130.5 (CH), 129.7 (CH), 127.8 (CH), 126.0 (CH), 125.4 (CH), 123.6 (CH), 122.5 (CH), 119.8 (C_q), 116.4 (C_q), 94.8 (d, $^1J_{C\text{-}Rh}$= 6.5 Hz, C_q), 25.2 (d, $^1J_{C\text{-}Rh}$ = 1.3 Hz, CH_3), 21.4 (CH_3) , 21.3 (CH_3), 9.4 (CH_3). **IR** (ATR): 3365, 3117, 2909, 1646, 1574, 1510, 1391, 1025, 817, 773 cm^{-1}. **HR-MS** (ESI) *m/z* calcd for $RhC_{33}H_{34}N_2$ $[M\text{-}OAc]^+$: 561.1772, found: 561.1771.

5.2. Catalytic Reactivity of Rhodacycles 4 and 5

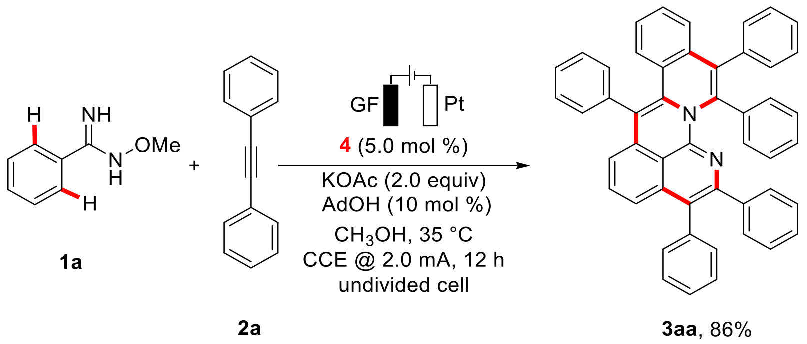

The electrocatalysis was carried out in an undivided cell with a graphite felt anode (10 mm × 15 mm × 6 mm) and a Pt cathode (10 mm × 15 mm × 0.25 mm). The cell was charged with imidate **1a** (0.2 mmol), alkyne **2a** (0.7 mmol), KOAc (39.3 mg, 0.4 mmol), AdOH (3.6 mg, 10 mol %), **4** (4.5 mg, 5.0 mol %) and MeOH (4.0 mL). Electrocatalysis was performed at 35 °C with a constant current of 2.0 mA maintained for 12 h. The graphite felt anode was washed with CH_2Cl_2 (8 × 5.0 mL) in an ultrasonic bath. Evaporation of the solvents and subsequent column chromatography on silica gel (*n*-hexane/EtOAc = 50 : 1) afforded the corresponding product **3aa** (112.0 mg, 86%).

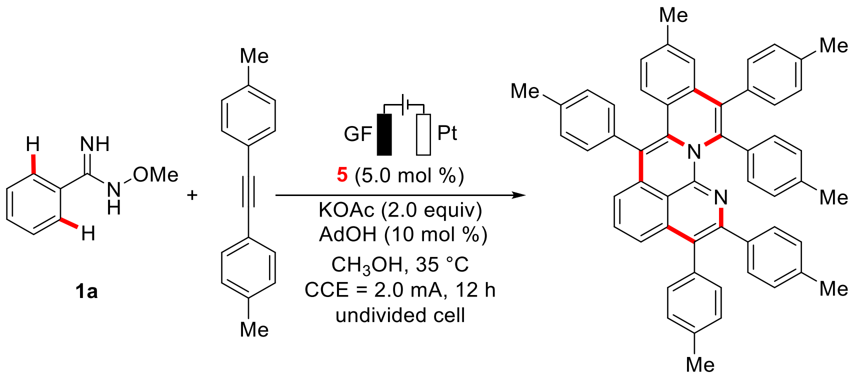

1a **2b** **3ab**, 51%

The electrocatalysis was carried out in an undivided cell with a graphite felt anode (10 mm × 15 mm × 6 mm) and a Pt cathode (10 mm × 15 mm × 0.25 mm). The cell was charged with imidate **1a** (0.2 mmol), alkyne **2b** (0.7 mmol), KOAc (39.3 mg, 0.4 mmol), AdOH (3.6 mg, 10 mol %), **5** (6.2 mg, 5.0 mol %) and MeOH (4.0 mL). Electrocatalysis was performed at 35 °C with a constant current of 2.0 mA maintained for 12 h. The graphite felt anode was washed with CH_2Cl_2 (8 × 5.0 mL) in an ultrasonic bath. Evaporation of the solvents and subsequent column chromatography on silica gel (*n*-hexane/EtOAc = 50 : 1) afforded the corresponding product **3ab** (75.2 mg, 51%).

5.3 Cyclic Voltammetry

Cyclic voltammetry measurements were conducted with a Metrohm Autolab PGSTAT204 potentiostat and Nova 2.1 software. For all experiments a glassy carbon

working electrode (disk, diameter: 3 mm), a platinum wire counter electrode, and an aqueous SCE (saturated calomel electrode) reference electrode were employed. Methanol with 0.1 mol/L *n*-Bu_4NPF_6 and dichloromethane with 0.1 mol/L *n*-Bu_4NPF_6 as conducting salt served as electrolytes for the measurements. The solvents were dried and degassed prior to their use. The voltammograms were referenced internally versus ferrocene and recorded at a scan rate of 100 mV/s, if not indicated otherwise.

CV studies of complex 5

Upon combination of complex **5** and alkyne **2b** and subsequent heating of the mixture a new process appears at 0.23 V versus ferrocene (Figure S1). This process intensifies upon addition of potassium acetate. Comparing to very recent studies this may indicate an oxidatively induced reductive elimination pathway for the second alkyne annulation of the cascade C–H activation.[3]

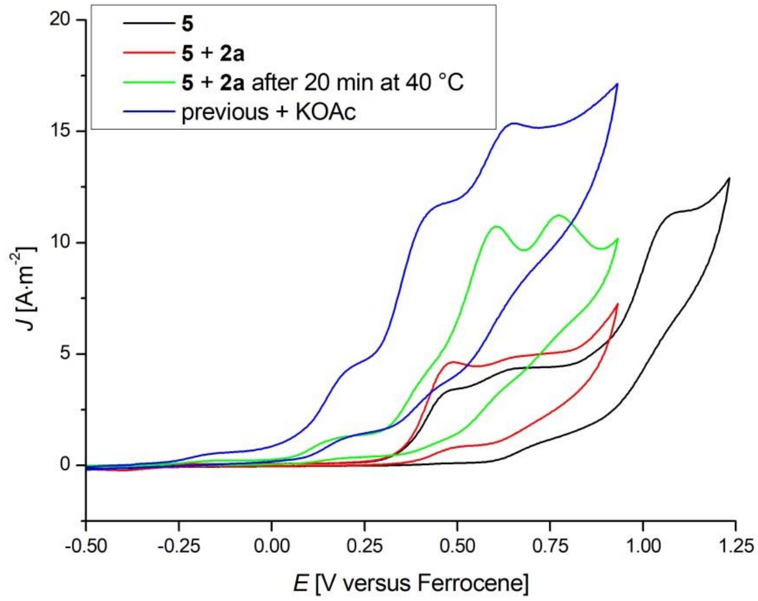

Figure S1. Cyclic voltammetry of complex **5** (2 mM, black), the mixture of **5** (2 mM) and **2b** (10 mM, red), the mixture of **5** (2 mM) and **2b** (10 mM) after heating to 40 °C for 20 minutes (green), and this mixture after addition of KOAc (10 mM, blue), all in methanol with *n*-Bu_4NPF_6 (0.1 M) at 0.1 V/s.

CV studies of 3aa and 6

In addition, we investigated the products **3aa** and **6** by cyclic voltammetry in dichloromethane. Aza-PAH **3aa** is characterized by a chemically reversible redox

process at $E_{1/2}$ = 0.16 V and a chemically irreversible process with an anodic peak potential of E_{pa} = 0.88 V (scan rate: 0.1 V/s). The chemical reversibility of the first process is only then evident, when a vertex potential before the second oxidation wave is selected (green line in Figure S2). If the second oxidation is crossed the reductive wave of the previous process disappears completely (scan rate: 0.1 V/s). The chemical follow up reaction of the electrochemical oxidation at higher potentials leads to one ore more substances, which are responsible for additional small processes developing at e.g. E_{pc} = -0.34 V, E_{pc} = -1.80 V, and E_{pa} = -0.08 V versus ferrocene (**Figure S2** and **Figure S3**).

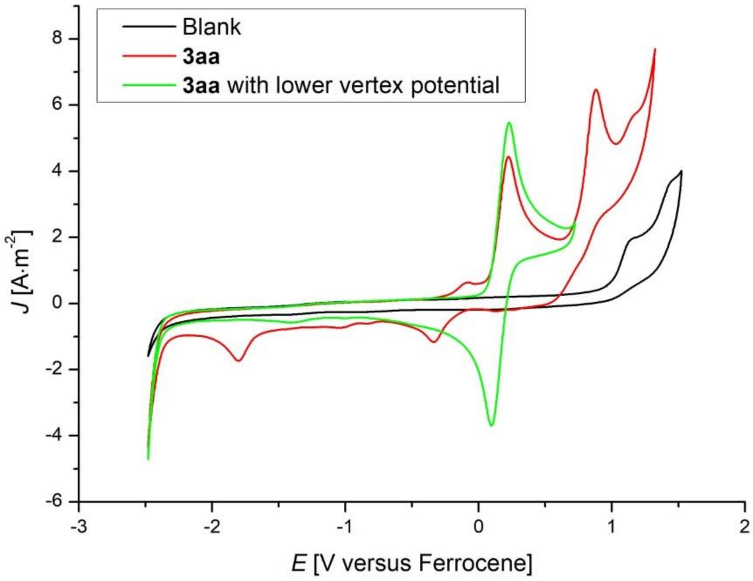

Figure S2. Cyclic voltammetry of the blank spectrum (black), compound **3aa** (2 mM) with a vertex potential at 1.32 V, and compound **3aa** (2 mM) with a vertex potential at 0.72 V, all in dichloromethane with *n*-Bu_4NPF_6 (0.1 M) at 0.1 V/s.

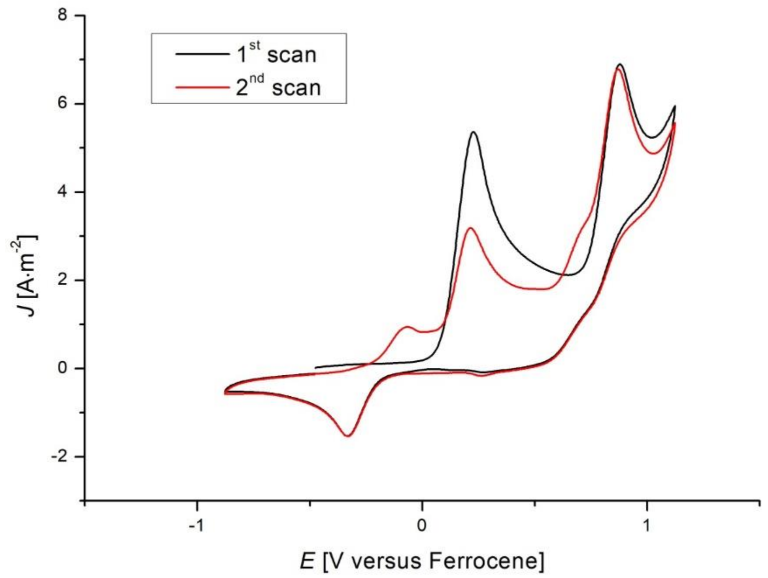

Figure S3. Comparison of the first and second scan during cyclic voltammetry of compound **3aa** (2 mM) with a vertex potential at 1.12 V, in dichloromethane with *n*-Bu_4NPF_6 (0.1 M) at 0.1 V/s.

If measuring at high scan rates both processes appear chemically reversible (**Figure S4**).

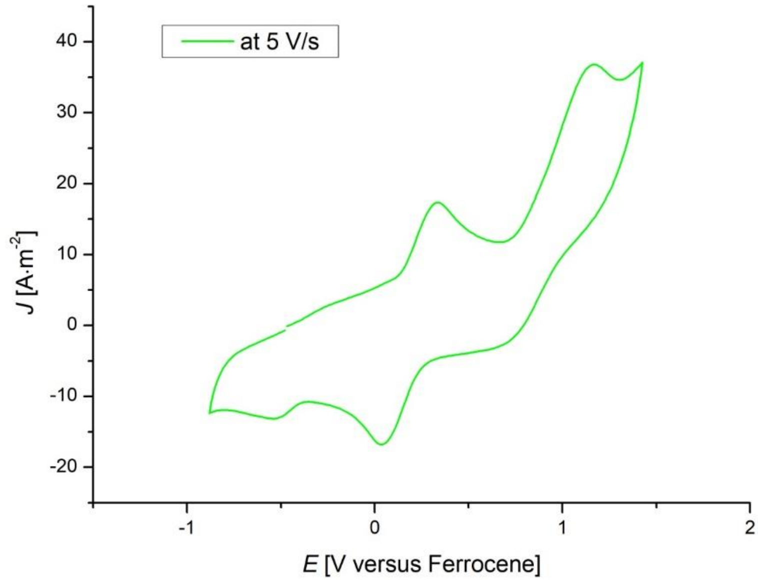

Figure S4. Cyclic voltammetry of compound **3aa** (2 mM) with a vertex potential at 1.42 V at a scan rate of 5 V/s, in dichloromethane with *n*-Bu_4NPF_6 (0.1 M).

Compound **6** shows a reversible redox process at $E_{1/2}$ = -1.72 V (scan rate dependence in **Figure S5**) and a reversible redox process at $E_{1/2}$ = 0.91 V. **Table S1** summarizes the CV data for compounds **3aa** and **6**.

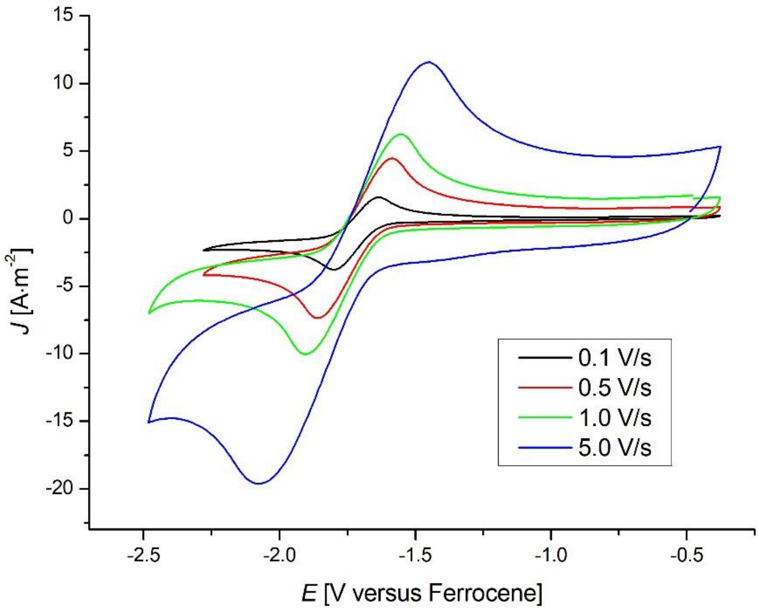

Figure S5. Cyclic voltammetry of compound **6** (2 mM) at varied scan rates, in dichloromethane with *n*-Bu_4NPF_6 (0.1 M).

Table S1. CV data for compounds **3aa** and **6** in dichloromethane with *n*-Bu_4NPF_6 (0.1 M).

Compound	E_{pc} [V vs. Fc]	E_{pa} [V vs. Fc]	$E_{1/2}$ [V vs. Fc]
**3aa**	0.10	0.23	0.16
	-	0.88	-
**3aa** at 5 V/s	0.05	0.34	0.20
	0.65	1.16	0.91
**6**	-1.80	-1.64	-1.72
		0.37	
	0.81	1.01	0.91

6. UV-Vis Absorption and Emission Spectra of 3aa

Absorption spectrum of **3aa**

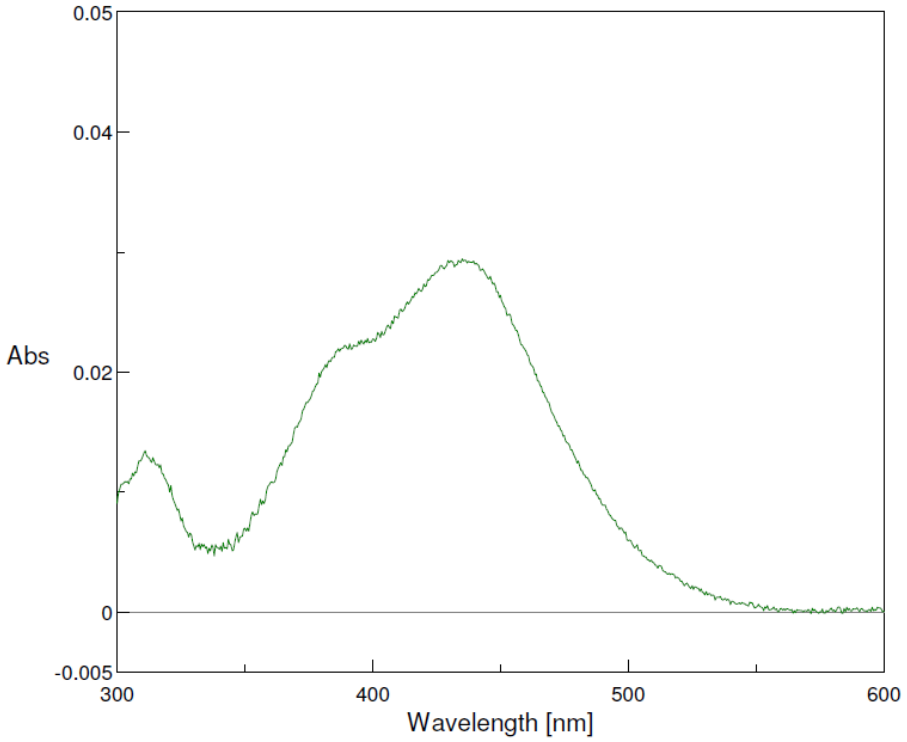

Emission spectrum of **3aa**

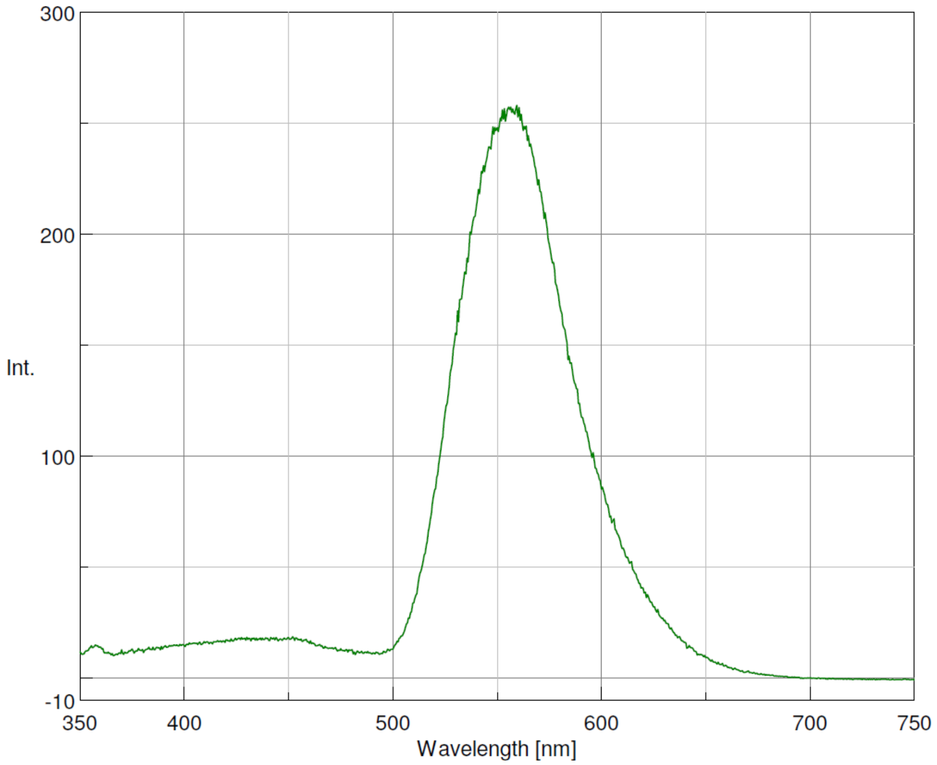

7. Transformation of Product 3aa and 3aj

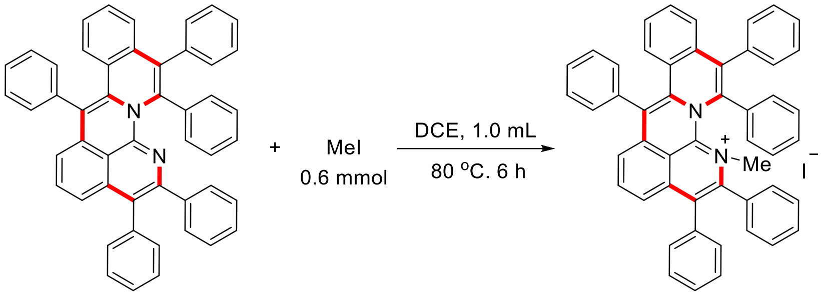

3aa (0.1 mmol) **6**, 93%

To a 25 mL Schlenk tube was added **3aa** (65.2 mg, 0.1 mmol), MeI (85 mg, 0.6 mmol, 20 µl) and 1.0 mL DCE. The reaction was stirred at 80 °C for 6 h. Then the solvent was removed and purification of the residue by column chromatography (DCM/MeOH = 50 : 1) yielded the product **6** (73.4 mg, 93%) as a red solid. M. p. = 229–230 °C. **^{1}H NMR** (400 MHz, $CDCl_3$) δ = 7.93 (t, J = 8.0 Hz, 1H), 7.81 (d, J = 7.7 Hz, 1H), 7.74 (t, J = 7.2 Hz, 1H), 7.61 (t, J = 7.4 Hz, 1H), 7.54 – 7.50 (m, 3H), 7.45 – 7.35 (m, 7H), 7.32 – 7.27 (m, 3H), 7.26 – 6.98 (m, 13H), 6.55 (d, J = 7.6 Hz, 1H), 5.51 (d, J = 7.7 Hz, 1H), 3.63 (s, 3H). **^{13}C NMR** (101 MHz, $CDCl_3$) δ = 151.9 (C_q), 144.3 (C_q), 136.0 (CH), 135.8 (C_q), 135.5 (C_q), 135.4 (C_q, overlapped, 2C), 135.1 (C_q), 135.0 (C_q), 134.6 (C_q), 134.1 (C_q), 133.6 (C_q), 132.5 (C_q), 132.0 (CH), 131.7 (C_q), 131.7 (C_q), 131.6 (CH), 130.9 (CH), 130.7 (CH), 130.4 (CH), 130.3 (CH), 130.0 (CH), 129.7 (CH), 129.5 (CH), 129.1 (CH), 129.0 (CH), 128.8 (CH), 128.7 (CH), 128.4 (CH), 128.4 (CH), 128.3 (CH), 128.2 (CH), 128.1 (CH), 127.6 (C_q), 126.8 (CH), 125.4 (C_q), 123.6 (CH), 122.6 (CH), 122.6 (C_q), 46.9 (CH_3). **IR (ATR)**: 3031, 3004, 1619, 1549, 1439, 1328, 1246, 758, 697 cm^{-1}. **HR-MS** (ESI) m/z calcd for $C_{50}H_{34}N_2$ $[M-I]^+$: 663.2795, found: 663.2799.

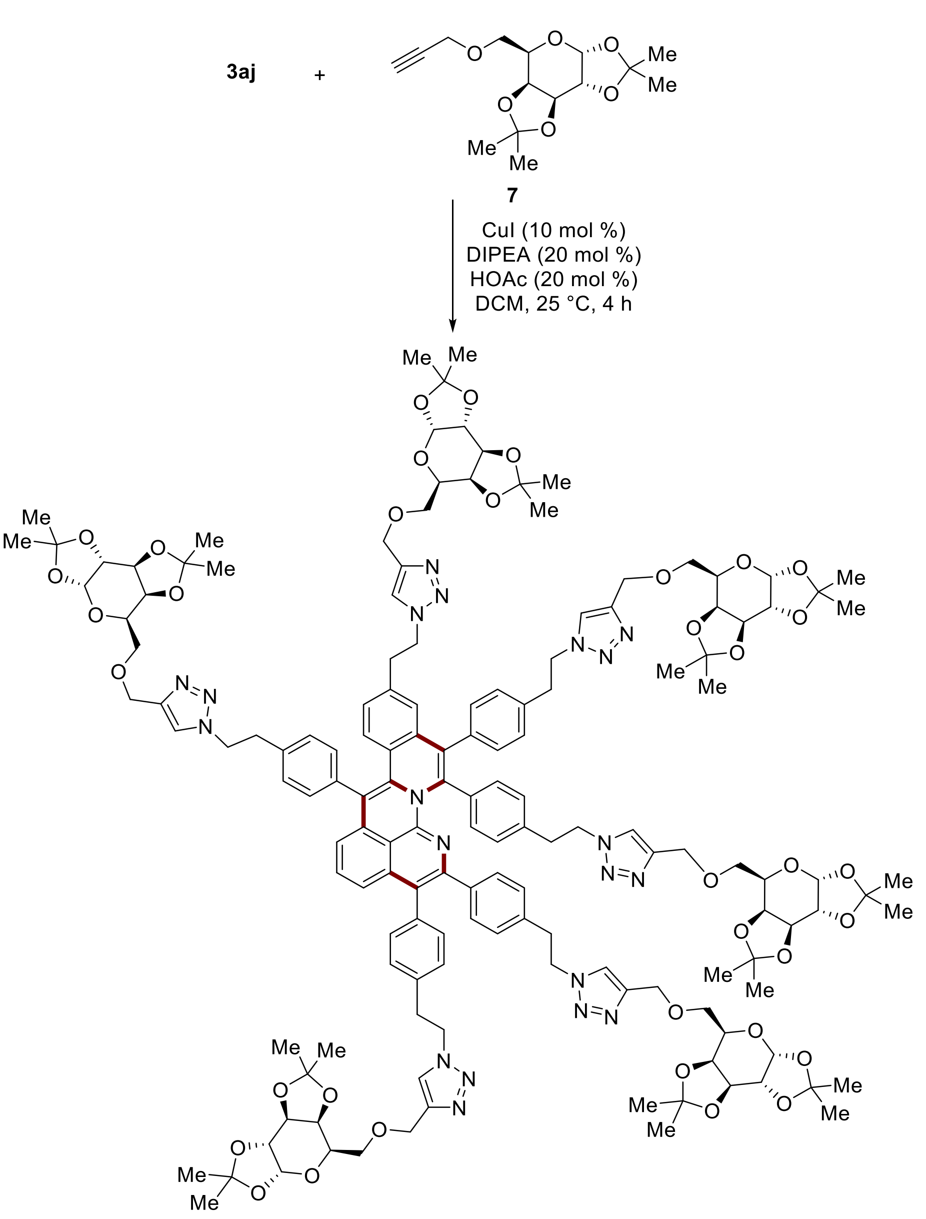

8, 88%

A 15 mL reaction tube was charged with CuI (1.9 mg, 0.005 mmol), acetic acid (0.01 mmol, 10 μL, 1.0 M in CH_2Cl_2) and *N*,*N*-diisopropylethylamine (0.01 mmol, 10 μL, 1.0 M in CH_2Cl_2) and CH_2Cl_2 (0.5 mL). Then, **3aj** (53.0 mg, 0.05 mmol) and alkyne **7** (107.3 mg, 0.36 mmol) were added to the mixture, which was stirred at 25 °C for 4 h. Evaporation of the solvent and subsequent column chromatography on silica gel (CH_2Cl_2/MeOH = 30 : 1) afforded product **8** as red solid (125.5 mg, 88%). M. p. = 103–104 °C. **^{1}H NMR** (400 MHz, $CDCl_3$) δ = 7.60 – 7.56 (m, 2H), 7.44 – 7.38 (m, 6H), 7.10 – 7.03 (m, 4H), 7.02 – 6.95 (m, 4H), 6.82 (t, J = 9.6 Hz, 4H), 6.77 – 6.69 (m, 4H), 6.41 (d, J = 7.9 Hz, 2H), 5.53 – 5.48 (m, 6H), 4.74 – 4.53 (m, 30H),

4.50 – 4.43 (m, 6H), 4.31 – 4.26 (m, 6H), 4.25 – 4.19 (m, 6H), 4.03 – 3.94 (m, 6H), 3.73 – 3.60 (m, 12H), 3.25 (t, J = 6.8 Hz, 2H), 3.26 – 3.16 (m, 4H), 3.09 – 3.02 (m, 6H), 1.52 – 1.48 (m, 18H), 1.43 – 1.38 (m, 18H), 1.33 – 1.27 (m, 36H). **^{13}C NMR** (100 MHz, $CDCl_3$) δ = 149.2 (C_q), 149.2 (C_q), 145.2 (C_q), 145.2 (C_q), 145.0 (C_q), 144.8 (C_q, overlapped, 2C), 139.3 (C_q), 144.8 (C_q), 137.7 (C_q), 137.5 (C_q), 137.4 (C_q), 137.0 (C_q), 136.4 (C_q), 135.9 (C_q), 135.8 (C_q), 135.7 (C_q, overlapped, 2C), 135.6 (C_q), 135.5 (C_q), 135.3 (C_q), 135.3 (C_q), 135.1 (C_q), 134.9 (C_q), 132.1 (CH), 132.0 (CH), 131.6 (CH), 131.2 (CH), 130.8 (CH), 130.5 (CH), 130.2 (CH), 129.0 (CH), 128.9 (CH), 128.7 (CH), 128.5 (CH), 127.8 (CH), 127.3 (CH, overlapped, 2C), 126.5 (CH), 126.1 (C_q), 124.9 (C_q), 124.8 (CH), 123.0 (CH), 122.9 (C_q), 122.8 (CH), 122.7 (CH), 122.7 (CH), 121.7 (C_q), 119.1 (CH), 118.4 (C_q), 117.6 (CH), 109.2 (C_q, overlapped, 2C), 109.2 (C_q), 109.2 (C_q, overlapped, 3C), 108.5 (C_q, overlapped, 3C), 108.5 (C_q, overlapped, 3C), 96.3 (CH, overlapped, 6C), 71.2 (CH, overlapped, 4C), 71.1 (CH, overlapped, 2C), 70.6 (CH, overlapped, 6C), 70.5 (CH, overlapped, 2C), 70.5 (CH, overlapped, 4C), 69.3 (CH_2), 69.2 (CH_2, overlapped, 3C), 69.2 (CH_2, overlapped, 2C), 66.8 (C_q, overlapped, 2C), 66.7 (C_q), 66.7 (C_q, overlapped, 3C), 64.8 (CH_2), 64.7 (CH_2, overlapped, 3C), 64.7 (CH_2, overlapped, 2C), 51.5 (CH_2, overlapped, 3C), 51.3 (CH_2), 51.3 (CH_2), 50.9 (CH_2), 36.6 (CH_2), 36.5 (CH_2), 36.5 (CH_2), 36.4 (CH_2), 36.3 (CH_2), 36.3 (CH_2), 26.1 (CH_3, overlapped, 6C), 26.0 (CH_3, overlapped, 6C), 24.9 (CH_3, overlapped, 6C), 24.4 (CH_3, overlapped, 3C), 24.4 (CH_3, overlapped, 3C). **IR** (ATR): 2986, 2933, 2171, 2096, 2031, 1956, 1379, 1213, 1069, 1006, 755 cm^{-1}. **HR-MS** (ESI) *m/z* calcd for $C_{151}H_{182}N_{20}O_{36}Na$ $[M+Na]^+$: 2874.2918, found: 2874.2921.

8. X-Ray Crystallographic Analysis

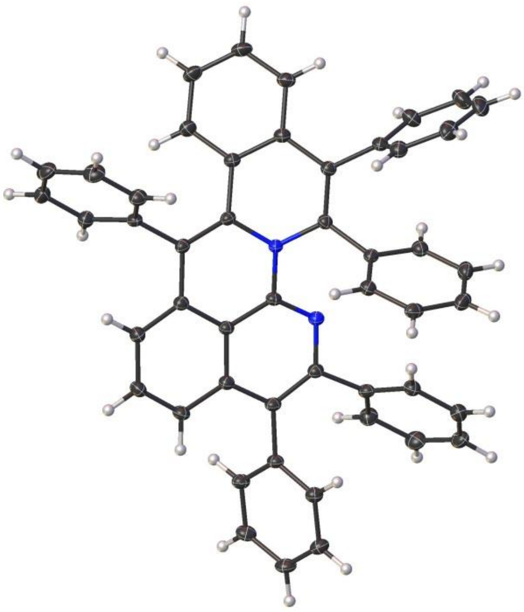

3aa, CCDC: 1963514

**Crystal data and structure refinement for 0739_CG_0m.**	
Identification code	0739_CG_0m
Empirical formula	$C_{49}H_{32}N_2$
Formula weight	648.76
Temperature/K	100.02
Crystal system	triclinic
Space group	P-1
a/Å	12.4709(19)
b/Å	12.9320(18)
c/Å	12.9338(15)
α/°	109.251(4)
β/°	113.349(4)
γ/°	102.261(4)
Volume/Å^3	1660.4(4)
Z	2
ρ_{calc}g/cm^3	1.298
μ/mm^{-1}	0.075
F(000)	680.0
Crystal size/mm^3	0.432 × 0.187 × 0.06
Radiation	MoKα (λ = 0.71073)
2Θ range for data collection/°	5.118 to 57.484

Index ranges	$-16 \le h \le 16, -17 \le k \le 17, -17 \le l \le 15$
Reflections collected	82539
Independent reflections	8607 [R_{int} = 0.0286, R_{sigma} = 0.0160]
Data/restraints/parameters	8607/0/460
Goodness-of-fit on F^2	1.054
Final R indexes [I>=2σ (I)]	R_1 = 0.0440, wR_2 = 0.1182
Final R indexes [all data]	R_1 = 0.0474, wR_2 = 0.1212
Largest diff. peak/hole / e $Å^{-3}$	0.43/-0.24

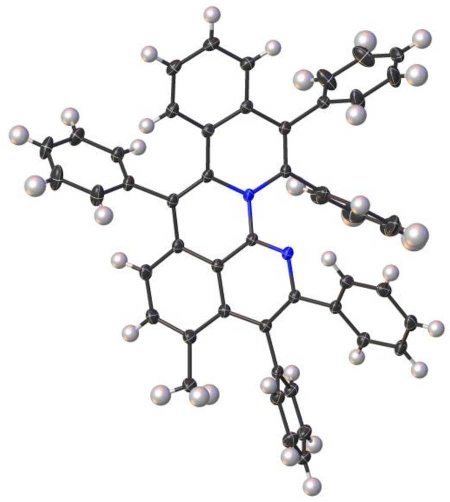

3ja, CCDC: 1969359

Identification code	mo_0855_CG_0m
Empirical formula	$C_{50}H_{34}N_2$
Formula weight	662.79
Temperature/K	100.0
Crystal system	triclinic
Space group	P-1
a/Å	10.7998(14)
b/Å	13.4372(19)
c/Å	14.2355(15)
α/°	115.427(4)
β/°	94.110(4)
γ/°	107.077(4)
Volume/$Å^3$	1736.0(4)
Z	2
ρ_{calc}g/cm^3	1.268
μ/mm^{-1}	0.073

F(000)	696.0
Crystal size/mm^3	0.17 × 0.12 × 0.04
Radiation	MoKα (λ = 0.71073)
2Θ range for data collection/°	4.58 to 59.256
Index ranges	$-15 \leq h \leq 14$, $-18 \leq k \leq 18$, $-19 \leq l \leq 18$
Reflections collected	61986
Independent reflections	9651 [R_{int} = 0.0315, R_{sigma} = 0.0223]
Data/restraints/parameters	9651/0/470
Goodness-of-fit on F^2	1.042
Final R indexes [I>=2σ (I)]	R_1 = 0.0506, wR_2 = 0.1356
Final R indexes [all data]	R_1 = 0.0577, wR_2 = 0.1415
Largest diff. peak/hole / e Å^{-3}	0.46/-0.26

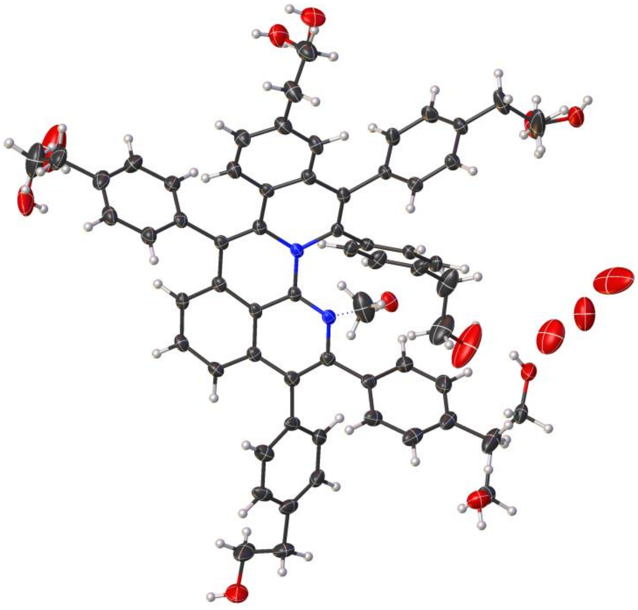

3ah, CCDC: 1964449

Identification code	mo_0829_CG_0m_4
Empirical formula	$C_{62}H_{62.72}N_2O_{8.36}$
Formula weight	969.62
Temperature/K	100.0
Crystal system	triclinic
Space group	P-1

a/Å	13.039(5)
b/Å	13.666(6)
c/Å	15.552(7)
α/°	100.671(10)
β/°	93.567(10)
γ/°	108.865(11)
Volume/Å^3	2554.6(18)
Z	2
ρ_{calc}g/cm^3	1.261
μ/mm^{-1}	0.083
F(000)	1031.0
Crystal size/mm^3	0.4 × 0.35 × 0.09
Radiation	MoKα (λ = 0.71073)
2Θ range for data collection/°	4.47 to 54.374
Index ranges	-16 ≤ h ≤ 16, -17 ≤ k ≤ 17, -19 ≤ l ≤ 19
Reflections collected	18816
Independent reflections	18816 [R_{int} = ?, R_{sigma} = 0.0517]
Data/restraints/parameters	18816/3/726
Goodness-of-fit on F^2	1.052
Final R indexes [I>=2σ (I)]	R_1 = 0.0722, wR_2 = 0.1744
Final R indexes [all data]	R_1 = 0.0920, wR_2 = 0.1871
Largest diff. peak/hole / e Å^{-3}	0.74/-0.59

Severe disorder is found for all hydroxy arms. The disorder of the water molecule O12>14 might possibly be a methanol, but no stable model was found for that. Some hydrogen atoms were positioned on arbitrary positions that make chemical sense in terms of hydrogen bonds. For the disordered water molecules no stable hydrogen atom positions were found, so they were left naked, although this is not ideal. Furthermore, there might be a connection between the disorder and the twinning, as BASF and occupancy of some Parts refine to very similar values. The nature of this connection was not found yet. Surprisingly, the data reduction appears rather good, given the Rint, I/sigma etc. The high R-value must be due to the non-trivial model.

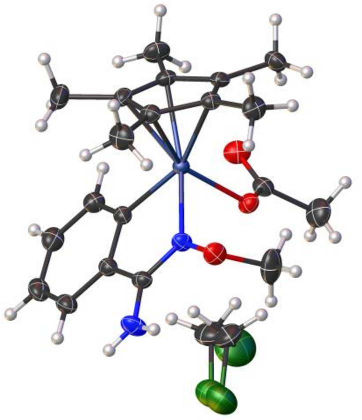

4, CCDC: 1963501

**Crystal data and structure refinement for 0798_CG_0m.**	
Identification code	0798_CG_0m
Empirical formula	$C_{21}H_{29}Cl_2N_2O_3Rh$
Formula weight	531.27
Temperature/K	150.0
Crystal system	monoclinic
Space group	$P2_1/n$
a/Å	11.0806(4)
b/Å	14.4903(5)
c/Å	14.8038(6)
α/°	90
β/°	103.698(2)
γ/°	90
Volume/Å^3	2309.31(15)
Z	4
ρ_{calc}g/cm^3	1.528
μ/mm^{-1}	0.995
F(000)	1088.0
Crystal size/mm^3	0.402 × 0.342 × 0.328
Radiation	MoKα (λ = 0.71073)
2Θ range for data collection/°	4.714 to 57.556
Index ranges	$-14 \leq h \leq 14$, $-19 \leq k \leq 19$, $-19 \leq l \leq 20$
Reflections collected	41085
Independent reflections	5927 [R_{int} = 0.0216, R_{sigma} = 0.0144]
Data/restraints/parameters	5927/3/303
Goodness-of-fit on F^2	1.066
Final R indexes [I>=2σ (I)]	R_1 = 0.0186, wR_2 = 0.0482

Final R indexes [all data]	R_1 = 0.0199, wR_2 = 0.0491
Largest diff. peak/hole / e Å^{-3}	0.36/-0.74

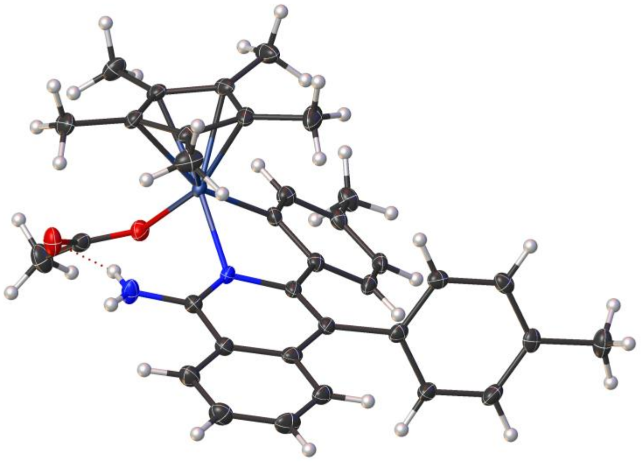

5, CCDC: 1963502

**Table 1 Crystal data and structure refinement for mo_0811_CG_0m_4.**	
Identification code	mo_0811_CG_0m_4
Empirical formula	$C_{35}H_{37}N_2O_2Rh$
Formula weight	620.57
Temperature/K	100.0
Crystal system	triclinic
Space group	P-1
a/Å	11.9810(5)
b/Å	14.0352(6)
c/Å	17.4705(7)
α/°	89.808(2)
β/°	85.5210(10)
γ/°	88.1530(10)
Volume/Å^3	2927.3(2)
Z	4
ρ_{calc}g/cm^3	1.408
μ/mm^{-1}	0.618
F(000)	1288.0
Crystal size/mm^3	0.191 × 0.142 × 0.06
Radiation	MoKα (λ = 0.71073)
2Θ range for data collection/°	3.982 to 57.446
Index ranges	-16 ≤ h ≤ 16, -18 ≤ k ≤ 18, -23 ≤ l ≤ 23
Reflections collected	25982

Independent reflections	25982 [R_{int} = ?, R_{sigma} = 0.0389]
Data/restraints/parameters	25982/0/754
Goodness-of-fit on F^2	1.040
Final R indexes [I>=2σ (I)]	R_1 = 0.0337, wR_2 = 0.0802
Final R indexes [all data]	R_1 = 0.0439, wR_2 = 0.0863
Largest diff. peak/hole / e $Å^{-3}$	0.74/-0.69

9. Proposed Mechanism

Based on following results, a mechanism for the rhodaelectro-catalyzed domino C–H activation was proposed:

1. The order of three C–H activation steps was confirmed by rhodacycles **4** and **5**.

2. The cleavage of the N–O bond in complex **5** and final products **3** proved that *N*-methoxylimidamide served as an internal oxidant in the first C–H functionalization step.

3. CV studies of **5** show a facile oxidation peak at 0.23 V (vs ferrocene), implying an oxidatively induced reductive elimination for the second and third C–N bonds formation.

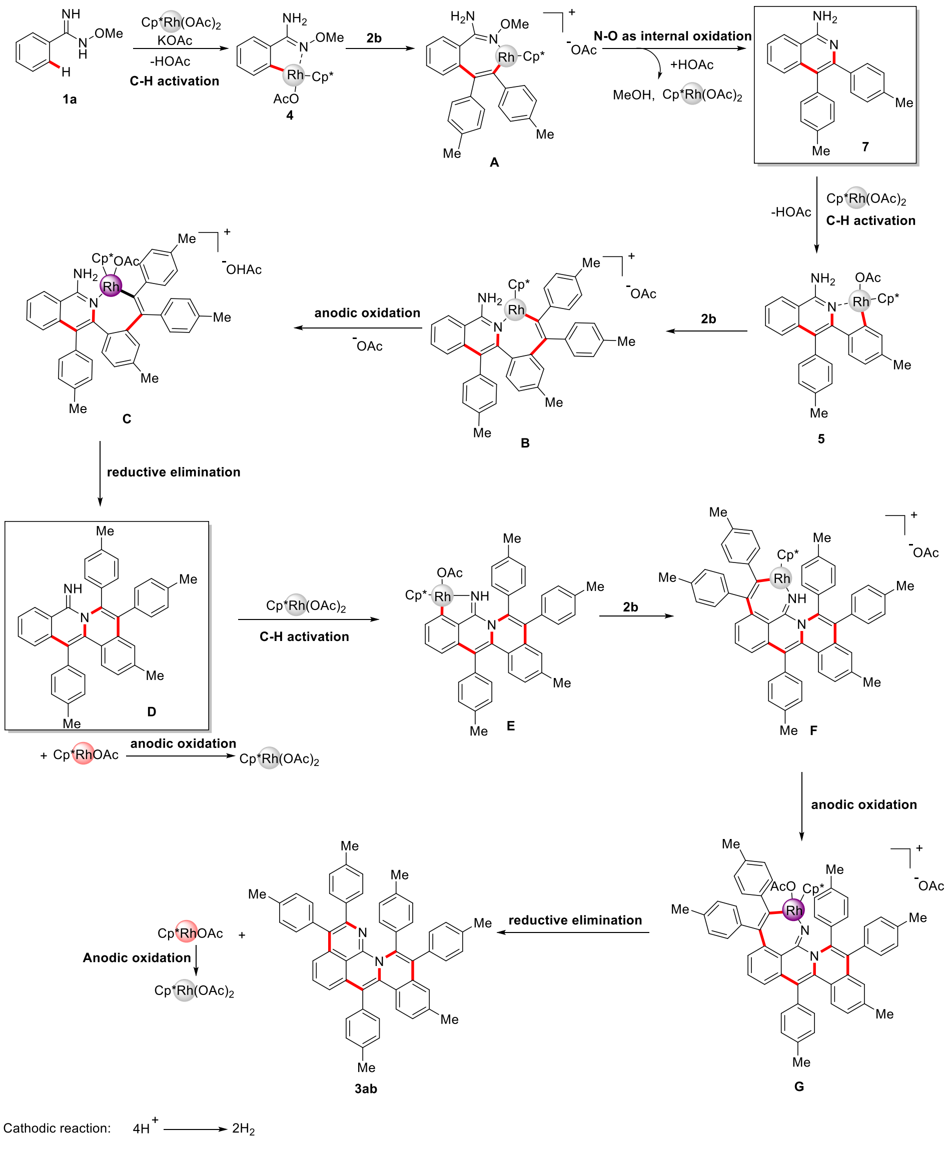

10. Characterization Data of Products

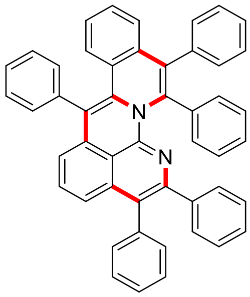

3aa

2,3,7,12,13-Pentaphenylbenzo[de]isoquinolino[2,1-a][1,8]naphthyridine (3aa)

The general procedure was followed using **1a** (30.0 mg, 0.2 mmol) and **2a** (124.6 mg, 0.7 mmol) at 35 °C for 12 h. Purification by column chromatography on silica gel (*n*-hexane/EtOAc = 50 : 1) yielded **3aa** (116.6 mg, 90%) as a red solid. M. p. = 295–296 °C. **^{1}H NMR** (400 MHz, $CDCl_3$) δ = 7.62 – 7.42 (m, 6H), 7.37 – 7.23 (m, 9H), 7.21 – 7.02 (m, 11H), 7.00 – 6.94 (m, 3H), 6.86 (ddd, J = 8.3, 7.1, 1.5 Hz, 1H), 6.60 – 6.56 (m, 2H). **^{13}C NMR** (100 MHz, $CDCl_3$) δ = 150.1 (C_q), 149.5 (C_q), 140.7 (C_q), 138.8 (C_q), 137.9 (C_q), 137.8 (C_q), 137.5 C_q), 137.1 (C_q, overlapped, 2C), 137.0 (C_q), 135.8 (C_q), 135.4 (C_q), 134.7 (C_q), 132.0 (CH), 131.9 (CH), 131.4 (CH), 131.1 (CH), 130.4 (CH, overlapped, 2C), 130.2 (CH), 129.9 (CH), 128.6 (CH), 128.4 (CH, overlapped, 2C), 128.1 (CH), 128.0 (CH), 127.5 (C_q), 127.1 (CH), 126.9 (CH), 126.8 (CH, overlapped), 126.7 (CH), 126.0 (CH), 125.7 (C_q), 125.0 (CH), 123.3 (C_q), 122.0 (C_q), 119.1 (CH), 117.7 (CH). **IR** (ATR): 1738, 1610, 1536, 1342, 1234, 757, 699 cm^{-1}. **HR-MS** (ESI) *m/z* calcd for $C_{49}H_{33}N_2$ $[M+H]^+$: 649.2638, found: 649.2635. The characterization data corresponds with those reported in the literature [4]

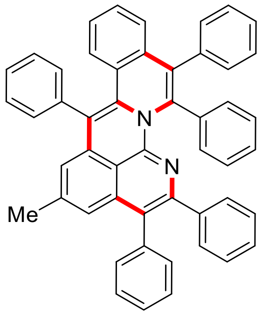

3ba

5-Methyl-2,3,7,12,13-pentaphenylbenzo[de]isoquinolino[2,1-a][1,8]naphthyridine

(3ba)

The general procedure was followed using **1b** (33.0 mg, 0.2 mmol) and **2a** (124.6 mg, 0.7 mmol) at 35 °C for 12 h. Purification by column chromatography on silica gel (*n*-hexane/EtOAc = 50 : 1) yielded **3ba** (124.0 mg, 94%) as a red solid. M. p. = 275–276 °C. **^{1}H NMR** (500 MHz, $CDCl_3$) δ = 7.54 – 7.50 (m, 4H), 7.49 – 7.44 (m, 1H), 7.28 – 7.19 (m, 8H), 7.12 – 6.92 (m, 12H), 6.89 (dd, J = 8.4, 6.9 Hz, 2H), 6.81 – 6.77 (m, 1H), 6.73 – 6.68 (m, 1H), 6.46 (d, J = 7.0 Hz, 2H), 2.28 (s, 3H). **^{13}C NMR** (125 MHz, $CDCl_3$) δ = 150.4 (C_q), 149.4 (C_q), 141.6 (C_q), 140.9 (C_q), 138.9 (C_q), 138.2 (C_q), 138.0 (C_q), 137.9 (C_q), 137.6 (C_q), 137.3 (C_q), 137.1 (C_q), 136.0 (C_q), 135.4 (C_q), 134.8 (C_q), 132.0 (CH), 132.0 (CH), 131.5 (CH), 130.4 (CH), 130.3 (CH), 130.0 (CH), 128.7 (CH), 128.5 (CH), 128.4 (CH), 128.1 (CH), 128.0 (CH), 127.6 (C_q), 127.2 (CH), 126.9 (CH), 126.8 (CH), 126.8 (CH), 126.7 (CH), 126.7 (CH), 126.0 (CH), 125.6 (C_q), 125.0 (CH), 123.0 (C_q), 120.6 (C_q), 119.2 (CH) 118.9 (CH), 22.8 (CH_3). **IR (ATR)**: 3055, 1612, 1542, 1338, 1278, 732, 697 cm^{-1}. **MS** (ESI) *m/z* (relative intensity): 663 (100) $[M+H]^+$, 685 (15) $[M+Na]^+$. **HR-MS** (ESI) *m/z* calcd for $C_{50}H_{35}N_2^+$ $[M+H]^+$: 663.2795, found: 663.2797. The characterization data corresponds with those reported in the literature [4]

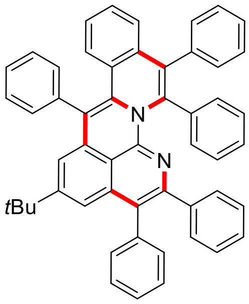

3ca

5-(*tert*-Butyl)-2,3,7,12,13-pentaphenylbenzo[de]isoquinolino[2,1-a][1,8] naphthyridine (3ca)

The general procedure was followed using **1c** (41.2.0 mg, 0.2 mmol) and **2a** (124.6 mg, 0.7 mmol) at 35 °C for 12 h. Purification by column chromatography on silica gel (*n*-hexane/EtOAc = 50 : 1) yielded **3ca** (92.0 mg, 66%) as a red solid. M. p. = 292–293 °C. **^{1}H NMR** (300 MHz, $CDCl_3$) δ = 7.61 – 7.49 (m, 5H), 7.33 – 7.24 (m,

9H), 7.20 – 7.12 (m, 5H), 7.11 – 6.91 (m, 9H), 6.87 – 6.80 (m, 1H), 6.53 (d, J = 7.0 Hz, 2H), 1.23 (s, 9H). **^{13}C NMR** (150 MHz, $CDCl_3$) δ = 154.3 (C_q), 150.3 (C_q), 149.3 (C_q), 141.0 (C_q), 139.0 (C_q), 138.1 (C_q), 137.7 (C_q), 137.6 (C_q), 137.3 (C_q), 137.2 (C_q), 135.7 (C_q), 135.0 (C_q), 134.8 (C_q), 132.1 (CH), 131.9 (CH), 131.4 (CH), 130.5 (CH), 130.5 (CH), 129.8 (CH), 128.8 (CH), 128.4 (CH), 128.3 (CH), 128.1 (CH), 128.0 (CH), 127.7 (C_q), 127.2 (CH), 126.9 (CH), 126.8 (CH), 126.8 (CH), 126.7 (CH), 126.6 (CH), 126.0 (CH), 125.5 (C_q), 125.0 (CH), 123.5 (C_q), 120.5 (C_q), 119.4 (C_q), 116.3 (CH), 115.2 (CH), 35.6 (C_q), 31.2 (CH_3). **IR (ATR)**: 3055, 2959, 1609, 1574, 1338, 1281, 909, 696 cm^{-1}. **MS** (ESI) m/z (relative intensity): 705 (100) $[M+H]^+$, 559 (15). **HR-MS** (ESI) m/z calcd for $C_{53}H_{40}N_2^+$ $[M+H]^+$: 705.3264, found: 705.3264. The characterization data corresponds with those reported in the literature [4]

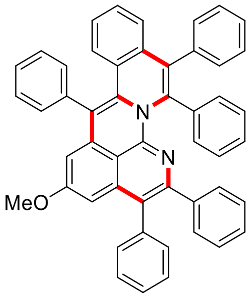

3da

5-Methoxy-2,3,7,12,13-pentaphenylbenzo[de]isoquinolino[2,1-a][1,8] naphthyridine (3da)

The general procedure was followed using **1d** (36.0 mg, 0.2 mmol) and **2a** (124.6 mg, 0.7 mmol) at 35 °C for 12 h. Purification by column chromatography on silica gel (n-hexane/EtOAc = 50 : 1) yielded **3da** (103.0 mg, 76%) as a red solid. M. p. > 300 °C. **^{1}H NMR** (400 MHz, $CDCl_3$) δ = 7.54 – 7.40 (m, 5H), 7.29 – 7.16 (m, 8H), 7.13 – 7.05 (m, 6H), 7.06 – 6.95 (m, 5H), 6.92 – 6.87 (m, 2H), 6.79 (ddd, J = 8.4, 6.6, 1.9 Hz, 1H), 6.61 (d, J = 2.2 Hz, 1H), 6.51 – 6.45 (m, 3H), 3.64 (s, 3H). **^{13}C NMR** (100 MHz, $CDCl_3$) δ = 162.0 (C_q), 150.7 (C_q), 149.0 (C_q), 140.7 (C_q), 139.8 (C_q), 138.6 (C_q), 138.1 (C_q), 137.4 (C_q), 137.3 (C_q), 137.0 (C_q), 136.4 (C_q, overlapped, 2C 134.6 (C_q), 131.9 (CH), 131.7 (CH), 131.2 (CH), 130.3 (CH, overlapped, 2C), 130.1 (CH), 129.9 (CH), 128.6 (CH), 128.4 (CH), 128.4 (CH), 128.0 (CH), 127.9 (CH), 127.3 (C_q), 127.0 (CH), 126.7 (CH, overlapped, 2C), 126.6 (CH), 126.5 (CH), 125.9

(CH), 125.4 (C_q), 124.9 (CH), 122.8 (C_q), 118.5 (C_q), 117.8 (Cq), 107.2 (CH), 100.2 (CH), 55.1(CH_3). **IR** (ATR): 1610, 1574, 1537, 1401, 1346, 1208, 759, 701 cm^{-1}. **HR-MS** (ESI) *m/z* calcd for $C_{50}H_{35}N_2O$ $[M+H]^+$: 679.2744, found: 679.2733. The characterization data corresponds with those reported in the literature [4]

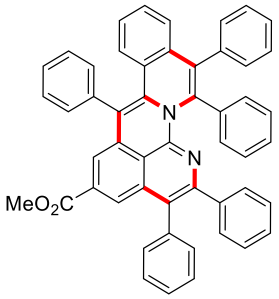

3ea

Methyl 2,3,7,12,13-pentaphenylbenzo[de]isoquinolino[2,1-a][1,8]naphthyridine-5-carboxylate (3ea)

The general procedure was followed using **1e** (41.6 mg, 0.2 mmol) and **2a** (124.6 mg, 0.7 mmol) at 35 °C for 12 h. Purification by column chromatography on silica gel (*n*-hexane/EtOAc = 50 : 1) yielded **3ea** (81.0 mg, 57%) as a red solid. M. p. = 193 – 194 °C. **^{1}H NMR** (400 MHz, $CDCl_3$) δ = 7.97 (d, *J* = 1.2 Hz, 1H), 7.59 – 7-50 (m, 6H), 7.33 – 7.26 (m, 8H), 7.17 – 6.99 (m, 11H), 6.97 – 6.91 (m, 2H), 6.86 (ddd, *J* = 8.4, 6.6, 1.9 Hz, 1H), 6.53 (d, *J* = 7.1 Hz, 2H), 3.84 (s, 3H). **^{13}C NMR** (100 MHz, $CDCl_3$) δ = 167.4 (C_q), 151.1 (C_q), 149.4 (C_q), 140.4 (C_q), 138.3 (C_q), 137.6 (C_q), 137.3 (C_q), 137.3 (C_q), 136.9 (C_q), 136.8 (C_q), 136.8 (C_q), 136.6 (C_q), 135.9 (C_q), 134.7 (C_q), 132.6 (C_q), 132.0 (CH), 131.8 (CH), 131.4 (CH), 130.4 (CH), 130.3 (CH), 130.2 (CH), 128.8 (CH), 128.7 (CH), 128.7 (CH), 128.5 (CH), 128.1 (CH), 128.1 (CH), 7.4 (C_q), 127.3 (CH), 127.2 (CH), 127.0 (CH), 127.0 (CH), 126.9 (CH), 126.2 (CH), 125.2 (CH), 124.2 (C_q), 123.4 (C_q), 120.8 (CH), 119.2 (C_q), 117.5 (CH), 52.5 (CH_3). **IR (ATR)**: 3058, 2919, 1725, 1609, 1541, 1339, 1229, 764, 669 cm^{-1}. **MS** (ESI) *m/z* (relative intensity): 707 (100) $[M+H]^+$, 739 (15) $[M+Na]^+$. **HR-MS** (ESI) *m/z* calcd for $C_{51}H_{34}N_2O_2^+$ $[M+H]^+$: 707.2693, found: 707.2695.

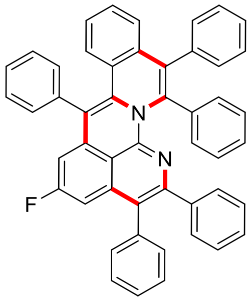

3fa

5-Fluoro-2,3,7,12,13-pentaphenylbenzo[de]isoquinolino[2,1-a][1,8]phthyridine (3fa)

The general procedure was followed using **1a** (33.6 mg, 0.2 mmol) and **2a** (124.6 mg, 0.7 mmol) at 35 °C for 12 h. Purification by column chromatography on silica gel (*n*-hexane/EtOAc = 50 : 1) yielded **3fa** (98.0 mg, 73%) as a red solid. M. p. = 278–280 °C. **^{1}H NMR** (300 MHz, $CDCl_3$) δ = 7.60 – 7.49 (s, 5H), 7.34 – 7.22 (m, 8H), 7.18 – 7.01 (m, 11H), 6.95 (t, J = 7.4 Hz, 2H), 6.89 – 6.82 (m, 2H), 6.62 (dd, J = 10.6, 1.9 Hz, 1H), 6.53 (d, J = 7.3 Hz, 2H). **^{13}C NMR** (100 MHz, $CDCl_3$) δ = 165.1 (C_q, $^1J_{C–F}$ = 247.1 Hz), 151.2 (C_q), 149.2 (C_q), 140.4 (C_q), 140.31 (C_q, $^3J_{C–F}$ = 11.2 Hz), 138.7 (C_q, ^3J = 11.0 Hz), 138.4 (C_q), 137.7 (C_q), 137.4 (C_q), 137.2 (C_q), 137.1 (C_q), 136.9 (C_q), 134.7 (C_q), 131.9 (CH), 131.8 (CH), 131.8 (CH), 131.2 (CH), 130.4 (CH), 130.2 (CH), 130.2 (CH), 128.8 (CH), 128.8 (CH) 128.7 (CH), 128.4 (CH), 128.1 (CH), 127.2 (CH), 127.2 (C_q), 127.1 (CH), 127.0 (CH), 127.0 (CH), 126.8 (CH), 126.2 (CH), 126.1 (C_q), 125.3 (CH), 123.32 (C_q, $^4J_{C–F}$ = 4.7 Hz), 119.2 (C_q), 118.5 (C_q), 105.9 (CH, $^2J_{C–F}$ = 27.3 Hz), 103.72 (CH, $^2J_{C–F}$ = 24.6 Hz). **^{19}F NMR** (282 MHz, $CDCl_{3)}$ δ = -105.93 (t, J = 10.7 Hz). **IR (ATR)**: 3051, 2961, 2161, 1609, 1572, 1342, 1021, 696 cm^{-1}. **MS** (ESI) *m/z* (relative intensity): 649 (100), 667 (15) $[M+H]^+$. **HR-MS** (ESI) *m/z* calcd for $C_{49}H_{32}FN_2$ $[M+H]^+$: 667.2544, found: 667.2531. The characterization data corresponds with those reported in the literature [4]

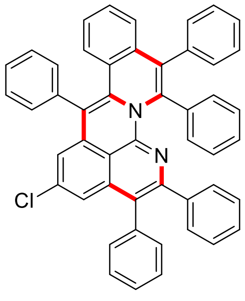

3ga

5-Chloro-2,3,7,12,13-pentaphenylbenzo[de]isoquinolino[2,1-a][1,8]naphthyridine (3ga)

The general procedure was followed using **1d** (36.8 mg, 0.2 mmol) and **2a** (124.6 mg, 0.7 mmol) at 35 °C for 12 h. Purification by column chromatography on silica gel (*n*-hexane/EtOAc = 50 : 1) yielded **3ga** (120.1 mg, 88%) as a red solid. M. p. = 177–178 °C. ^{1}**H NMR** (400 MHz, $CDCl_3$) δ = 7.57 – 7.44 (m, 5H), 7.30 – 7.17 (m, 8H), 7.15 – 6.95 (m, 12H), 6.94 – 6.88 (m, 2H), 6.84 – 6.79 (m, 2H), 6.50 – 6.47 (m, 2H). ^{13}**C NMR** (100 MHz, $CDCl_3$) δ = 151.2 (C_q), 149.1 (C_q), 140.2 (C_q), 139.0 (C_q), 138.1 (C_q), 138.0 (C_q), 137.3 (C_q), 137.2 (C_q, overlapped, 2C), 137.1 (C_q), 136.9 (C_q), 136.7 (C_q), 134.5 (C_q), 131.8 (CH), 131.6 (CH), 131.1 (CH), 130.2 (CH), 130.1 (CH), 130.0 (CH) 128.7 (CH), 128.6 (CH), 128.5 (CH), 128.3 (CH), 127.9 (CH), 127.1 (CH), 127.1 (C_q), 127.0 (CH), 126.8 (CH, overlapped, 3C), 126.7 (CH), 126.1 (CH), 126.0 (C_q), 125.1 (CH), 122.6 (C_q), 120.1 (C_q), 118.0 (C_q), 117.8 (CH), 117.4 (CH). **IR** (ATR): 1609, 1566, 1537, 1338, 1275, 878, 757, 701 cm^{-1}. **HR-MS** (ESI) *m/z* calcd for $C_{49}H_{32}N_2Cl$ $[M+H]^+$: 683.2249, found: 683.2247. The characterization data corresponds with those reported in the literature [4]

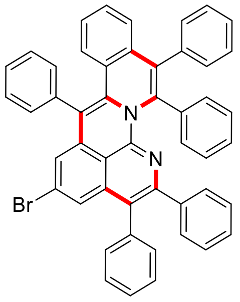

3ha

5-Bromo-2,3,7,12,13-pentaphenylbenzo[de]isoquinolino[2,1-a][1,8]naphthyridine

（**3ha**）

The general procedure was followed using **1a** (45.8 mg, 0.2 mmol) and **2a** (124.6 mg, 0.7 mmol) at 35 °C for 12 h. Purification by column chromatography on silica gel (*n*-hexane/EtOAc = 50 : 1) yielded **3ha** (112.0 mg, 77%) as a red solid. M. p. > 300 °C. **^{1}H NMR** (300 MHz, $CDCl_3$) δ 7.61 –7.49 (m, 5H), 7.34 – 7.21 (m, 9H), 7.16 – 6.99 (m, 12H), 6.98 – 6.91 (m, 2H), 6.86 (ddd, *J* = 8.4, 6.0, 2.5 Hz, 1H), 6.51 (d, *J* = 6.9 Hz, 2H). **^{13}C NMR** (101 MHz, $CDCl_3$) δ = 151.3 (C_q), 149.3 (C_q), 140.4 (C_q), 139.4 (C_q), 139.4 (C_q), 138.2 (C_q), 137.4 (C_q), 137.3 (C_q), 137.2 (C_q), 137.2 (C_q), 137.2 (C_q), 137.0 (C_q), 136.8 (C_q), 136.8 (C_q), 134.7 (C_q), 131.9 (CH), 131.8 (CH), 131.3 (CH), 130.4 (CH), 130.3 (CH), 130.2 (CH), 128.9 (CH), 128.8 (CH), 128.7 (CH), 128.5 (CH), 128.1 (CH), 127.3 (CH), 127.2 (CH), 127.1 (CH), 127.0 (CH), 127.0 (CH), 126.9 (CH), 126.3 (CH), 125.3 (CH), 122.6 (C_q), 121.0 (CH), 120.4 (C_q), 120.2 (CH), 118.1 (C_q). **IR (ATR)**: 3056, 2204, 1601, 1563, 1333, 1269, 694 cm^{-1}. **MS** (ESI) *m/z* (relative intensity):727 (100) $[M+H]^+$, 381 (15). **HR-MS** (ESI) *m/z* calcd for $C_{49}H_{32}N_2{}^{79}Br^+$ $[M+H]^+$: 727.1743, found: 727.1740. The characterization data corresponds with those reported in the literature [4]

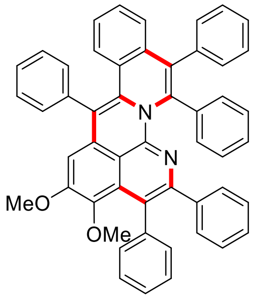

3ia

5,6-Dimethoxy-2,3,7,12,13-pentaphenylbenzo[de]isoquinolino[2,1-a][1,8] naphthyridine (3ia)

The general procedure was followed using **1i** (42.0 mg, 0.2 mmol) and **2a** (124.6 mg, 0.7 mmol) at 35 °C for 12 h. Purification by column chromatography on silica gel (*n*-hexane/EtOAc = 50 : 1) yielded **3ia** (120.4 mg, 85%) as a red solid. M. p. > 300 °C. A ratio of 6 : 1 for two regioisomers was observed. Spectra for major isomer: **^{1}H NMR** (500 MHz, $CDCl_3$) δ = 7.53 – 7.51 (m, 4H), 7.27 – 7.16 (m, 6H), 7.08 –

6.90 (m, 14H), 6.88 – 6.84 (m, 2H), 6.79 (ddd, J = 8.4, 5.9, 2.6 Hz, 1H), 6.62 (s, 1H), 6.43 – 6.39 (m, 2H), 3.71 (s, 3H), 3.03 (s, 3H). **^{13}C NMR** (125 MHz, $CDCl_3$) δ = 157.2 (C_q), 154.8 (C_q), 152.2 (C_q), 149.5 (C_q), 141.1 (C_q), 140.7 (C_q), 140.2 (C_q), 139.9 (C_q), 138.9 (C_q), 137.8 (C_q), 137.4 (C_q), 137.1(C_q), 135.3 (C_q), 135.3 (C_q), 134.7 (C_q), 132.9 (C_q), 131.9 (CH, overlapped, 2C), 131.5 (CH), 130.3 (CH), 130.1 (CH), 129.9 (CH), 128.4 (CH), 128.2 (CH), 128.1 (CH), 127.8 (CH), 127.4 (C_q), 126.9 (CH), 126.7 (CH), 126.4 (CH), 126.3 (CH), 126.1 (CH), 125.9 (CH), 125.5 (CH), 125.0 (CH), 120.9 (CH), 119.0 (C_q), 118.4 (C_q), 104.8 (CH), 60.3 (CH_3), 56.0 (CH_3). Selected distinct peaks for minor isomer: **^{1}H NMR** (500 MHz, $CDCl_3$) δ = 6.70 (s, 1H), 6.54 – 6.52 (m, 2H), 3.70 (s, 3H), 3.24 (s, 3H). **^{13}C NMR** (125 MHz, $CDCl_3$) δ = 101.3 (CH), 61.3 (CH_3), 55.5 (CH_3). **IR** (ATR): 3051, 1608, 1573, 1539, 1452, 1347, 767, 694 cm^{-1}. **HR-MS** (ESI) *m/z* calcd for $C_{51}H_{37}N_2O_2$ $[M+H]^+$: 709.2805, found: 709.2850.

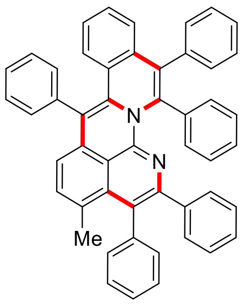

3ja

4-Methyl-2,3,7,12,13-pentaphenylbenzo[de]isoquinolino[2,1-a][1,8]naphthyridine (3ja)

The general procedure was followed using **1j** (32.8 mg, 0.2 mmol) and **2a** (124.6 mg, 0.7 mmol) at 35 °C for 12 h. Purification by column chromatography on silica gel (*n*-hexane/EtOAc = 50 : 1) yielded **3ja** (93.0 mg, 70%) as a red solid. M. p. = 230–231 °C. **^{1}H NMR** (400 MHz, $CDCl_3$) δ = 7.58 – 7.44 (m, 5H), 7.31 – 7.20 (m, 6H), 7.18 – 6.98 (m, 12H), 6.97 (d, J = 7.5 Hz, 2H), 6.91 (t, J = 7.3 Hz, 2H), 6.89 – 6.78 (m, 2H), 6.38 (d, J = 7.0 Hz, 2H), 1.81 (s, 3H). **^{13}C NMR** (100 MHz, $CDCl_3$) δ = 151.8 (C_q), 150.2 (C_q), 141.2 (C_q), 140.6 (C_q), 139.0 (C_q), 137.9 (C_q), 137.5 (C_q), 137.2 (C_q), 135.7 (C_q), 135.1 (CH), 134.8 (C_q), 134.7 (C_q), 134.2 (C_q), 132.4 (CH),

132.0 (CH), 132.0 (CH), 130.2 (CH), 130.0 (CH), 129.3 (C_q), 128.4 (CH), 128.3 (CH), 128.1 (CH), 127.9 (CH), 127.6 (CH), 127.5 (C_q), 127.1 (CH), 127.1 (CH), 126.8 (CH), 126.6 (CH), 126.6 (CH), 126.5 (CH), 126.2 (CH), 126.0 (CH), 125.5 (C_q), 125.0 (CH), 123.9 (C_q), 123.5 (C_q), 118.8 (C_q), 118.2 (CH), 24.1 (CH_3). **IR (ATR)**: 3054, 2923, 1735, 1603, 1539, 1441, 1323, 695 cm^{-1}. **MS** (ESI) *m/z* (relative intensity): 663 (100) $[M+H]^+$, 517 (10). **HR-MS** (ESI) *m/z* calcd for $C_{50}H_{35}N_2^+$ $[M+H]^+$: 663.2795, found: 663.2790.

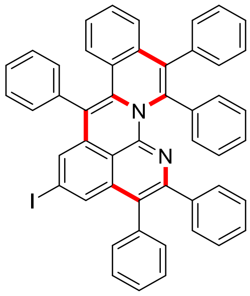

3ka

5-Iodo-2,3,7,12,13-pentaphenylbenzo[de]isoquinolino[2,1-a][1,8]naphthyridine (3ka)

The general procedure was followed using **1k** (55.2 mg, 0.2 mmol) and **2a** (124.6 mg, 0.7 mmol) at 35 °C for 12 h. Purification by column chromatography on silica gel (*n*-hexane/EtOAc = 50 : 1) yielded **3ka** (118.0 mg, 76%) as a red solid. M. p. = 175–176 °C. **^{1}H NMR** (400 MHz, $CDCl_3$) δ = 7.56 – 7.44 (m, 6H), 7.28 – 7.16 (m, 8H), 7.15 (d, J = 1.4 Hz, 1H), 7.13 – 6.95 (m, 11H), 6.93 – 6.86 (m, 2H), 6.81 (ddd, J = 8.4, 6.4, 2.1 Hz, 1H), 6.48 – 6.45 (m, 2H). **^{13}C NMR** (100 MHz, $CDCl_3$) δ = 151.0 (Cq), 149.3 (Cq), 140.2 (Cq), 139.1 (Cq), 138.1 (Cq), 137.2 (Cq), 137.2 (Cq), 136.9 (Cq), 136.8 (Cq, overlapped, 3C) 136.7 (Cq, overlapped, 2C), 134.5 (Cq), 131.8 (CH), 131.7 (CH), 131.2 (CH), 130.2 (CH), 130.1 (CH, overlapped, 2C), 128.7 (CH), 128.6 (CH), 128.5 (CH), 128.4 (CH), 127.9 (CH), 127.3 (CH), 127.1 (CH), 127.0 (CH), 126.8 (CH, overlapped, 3C), 126.7 (CH), 126.1 (CH), 125.8 (CH), 125.1 (CH), 122.0 (Cq), 120.5 (Cq), 117.6 (Cq), 99.9 (Cq). **IR** (ATR): 1666, 1607, 1561, 1335, 1259, 1072, 1019, 798, 700 cm^{-1}. **HR-MS** (ESI) *m/z* calcd for $C_{49}H_{32}N_2I$ $[M+H]^+$: 775.1605, found: 775.1594. The characterization data corresponds with those reported in the literature [4]

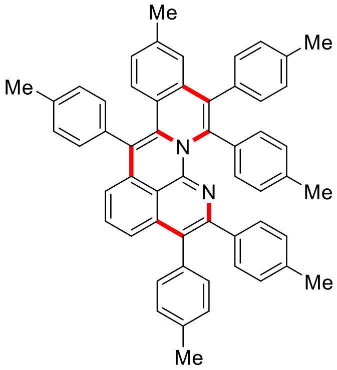

3ab

10-Methyl-2,3,7,12,13-penta-p-tolylbenzo[de]isoquinolino[2,1-a][1,8] naphthyridine (3ab)

The general procedure was followed using **1a** (30.0 mg, 0.2 mmol) and **2b** (144.4 mg, 0.7 mmol) at 35 °C for 12 h. Purification by column chromatography on silica gel (*n*-hexane/EtOAc = 50 : 1) yielded **3ab** (118.6 mg, 81%) as a red solid. M. p. = 198–199 °C. **^{1}H NMR** (400 MHz, $CDCl_3$) δ = 7.48 – 7.33 (m, 5H), 7.22 – 7.10 (m, 7H), 7.04 (d, J = 7.6 Hz, 4H), 7.00 – 6.90 (m, 3H), 6.87 (d, J = 8.0 Hz, 2H), 6.80 (d, J = 7.9 Hz, 2H), 6.69 (d, J = 8.3 Hz, 1H), 6.49 (d, J = 8.0 Hz, 2H), 2.51 (s, 3H), 2.40 (s, 3H), 2.38 (s, 3H), 2.27 (s, 3H), 2.24 (s, 3H), 2.20 (s, 3H). **^{13}C NMR** (100 MHz, $CDCl_3$) δ = 145.0 (C_q), 149.5 (C_q), 138.1 (C_q, overlapped 3C), 138.0 (C_q), 137.6 (C_q), 137.5 (C_q), 136.1 (C_q), 136.0 (C_q), 136.0 ($C_{q,}$ overlapped, 4C), 135.7 (C_q), 135.2 (C_q), 135.0 (C_q), 134.34 (C_q, overlapped, 2C), 131.8 (CH), 131.7 (CH), 131.2 (CH), 130.8 (CH), 130.6 (CH), 130.3 (CH), 130.1 (CH), 129.1 (CH), 128.6 (CH), 128.5 (CH), 127.7 (CH), 127.5 (CH) 127.0 (CH), 125.2 (C_q), 125.2 (CH), 122.8 (C_q), 121.7 (C_q), 118.7 (CH), 118.1 (C_q), 117.1 (CH), 21.5 (CH_3, overlapped, 2C), 21.4 (CH_3), 21.3 ($CH_{3,}$ overlapped, 2C) 21.2 (CH_3). **IR** (ATR): 2919, 1606, 1541, 1508, 1340, 907, 815, 730 cm^{-1}. **HR-MS** (ESI) *m/z* calcd for $C_{55}H_{45}N_2$ $[M+H]^+$: 733.3577, found: 733.3568. The characterization data corresponds with those reported in the literature [4]

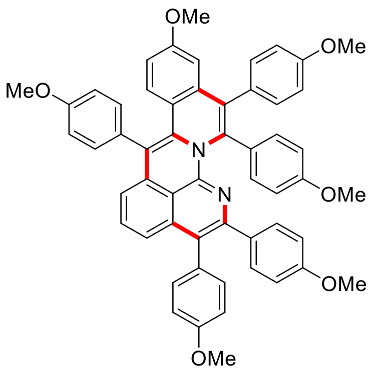

3ac

10-Methoxy-2,3,7,12,13-pentakis(4-methoxyphenyl)benzo[de]isoquinolino[2,1-a][1,8]naphthyridine (3ac)

The general procedure was followed using **1a** (7.5 mg, 0.05 mmol), **2c** (49.6 mg, 0.175 mmol) and [Cp*Rh(CH_3CN)$_3$](SbF_6)$_2$ (4.2 mg, 10 mol %) in a solvent mixture (4.0 mL, MeOH/*t*-AmOH = 7 : 1) at 35 °C for 3 h. Purification by column chromatography on silica gel (*n*-hexane/EtOAc = 50 : 1) yielded **3ac** (38.2 mg, 92%) as a red solid. M. p. = 177–178 °C. **^{1}H NMR** (400 MHz, $CDCl_3$) δ = 7.45 – 7.39 (m, 3H), 7.22 – 7.14 (m, 3H), 7.11 – 6.97 (m, 7H), 6.91 – 6.82 (m, 5H), 6.64 (d, J = 2.7 Hz, 1H), 6.60 (dd, J = 8.7, 5.3 Hz, 4H), 6.52 (d, J = 8.8 Hz, 2H), 6.48 – 6.42 (m, 1H), 3.93 (s, 3H), 3.84 (s, 3H), 3.82 (s, 3H), 3.73 (s, 3H), 3.71 (s, 3H), 3.65 (s, 3H). **^{13}C NMR** (100 MHz, $CDCl_3$) δ = 159.2 (C_q, overlapped, 2C), 158.3 (C_q, overlapped, 2C), 158.2 (C_q), 158.1 (C_q), 149.6 (C_q), 149.4 (C_q), 138.1 (C_q), 137.9 (C_q), 137.0 (C_q), 135.9 (C_q), 135.8 (C_q), 133.6 (C_q), 133.0 (CH), 132.8 (CH), 132.4 (CH), 131.6 (CH, overlapped, 2C), 131.4 (CH), 131.0 (C_q), 130.9 (CH), 130.5 (C_q), 130.1 (CH), 129.7 (C_q), 129.4 (C_q), 124.5 (C_q), 122.1 (C_q), 121.3 (C_q), 121.0 (C_q), 118.4 (CH), 116.9 (C_q), 116.8 (CH), 115.3 (CH), 113.9 (CH), 113.5 (CH), 112.5 (CH), 112.3 (CH), 109.2 (CH), 55.3 (CH_3, overlapped, 2C), 55.2 (CH_3, overlapped, 2C), 55.1 (CH_3), 55.0 (CH_3). **IR** (ATR): 2928, 1602, 1570, 1507, 1284, 1241, 1172, 1030, 826, 540 cm^{-1}. **HR-MS** (ESI) *m/z* calcd for $C_{55}H_{45}N_2O_6$ $[M+H]^+$: 829.3272, found: 829.3268. The characterization data corresponds with those reported in the literature [4]

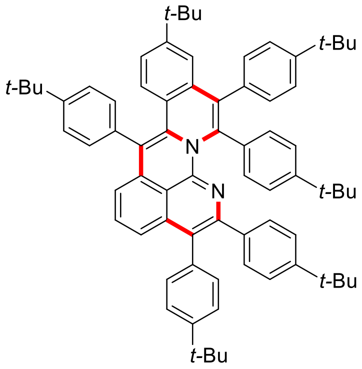

3ad

10-(*tert*-Butyl)-2,3,7,12,13-pentakis(4-(tert-butyl)phenyl)benzo[de]isoquinolino [2,1-a][1,8]naphthyridine (3ad)

The general procedure was followed using **1a** (30.0 mg, 0.2 mmol) and **2d** (203.4 mg, 0.7 mmol) at 35 °C for 12 h. Purification by column chromatography on silica gel (*n*-hexane/EtOAc = 50 : 1) yielded **3ad** (88.6 mg, 45%) as a red solid. M. p. = 205–206 °C. **^{1}H NMR** (400 MHz, $CDCl_3$) δ = 7.58 (d, J = 8.3 Hz, 2H), 7.49 (d, J = 8.0 Hz, 2H), 7.43 (t, J = 7.9 Hz, 1H), 7.30 (dd, J = 12.8, 4.1 Hz, 6H), 7.21 – 7.11(m, 2H), 7.10 – 7.00 (m, 6H), 6.97 – 6.88 (m, 4H), 6.85 (dd, J = 8.7, 2.0 Hz, 1H), 6.47 – 6.45 (s, 2H), 1.48 (s, 9H), 1.37 (s, 9H), 1.35 (s, 9H), 1.26 (s, 9H), 1.22 (s, 9H), 1.18 (s, 9H). **^{13}C NMR** (100 MHz, $CDCl_3$) δ = 151.0 (C_q), 150.8 (C_q), 150.1 (C_q), 149.5 (C_q), 149.4 (C_q), 149.3 (C_q), 149.0 (C_q), 148.9 (C_q), 138.0 (C_q), 137.9 (C_q), 137.5 (C_q), 136.0 (C_q), 135.9 (C_q), 135.8 (C_q), 135.2 (C_q), 134.5 (C_q), 134.4 (C_q), 134.3 (C_q), 131.6 (CH), 131.4 (CH), 130.9 (CH), 130.7 (CH), 130.0 (CH), 129.9 (C_q), 128.2 (CH), 126.7 (CH), 125.6 (C_q), 125.3 (C_q), 125.1 (CH), 124.4 (CH), 123.7 (CH), 123.5 (CH), 123.1 (CH), 122.6 (C_q), 121.6 (CH), 121.5 (CH), 118.5 (CH), 117.8 (C_q), 117.0 (CH), 34.8 (C_q), 34.6 (C_q), 34.5 ($C_{q,}$ overlapped, 2C), 34.4 (C_q), 34.2 (C_q), 31.5 (CH_3), 31.4 (CH_3, overlapped, 2C), 31.3 (CH_3, overlapped, 2C), 31.0 (CH_3). **IR** (ATR): 2959, 2902, 1611, 1577, 1507, 1339, 1268, 1110, 1018, 823 cm^{-1}. **HR-MS** (ESI) *m/z* calcd for $C_{73}H_{81}N_2$ $[M+H]^+$: 985.6394, found: 985.6379.

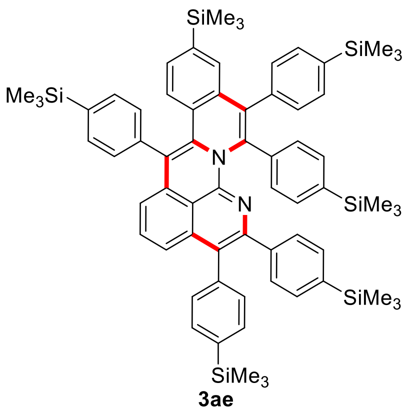

3ae

10-(Trimethylsilyl)-2,3,7,12,13-pentakis(4-(trimethylsilyl)phenyl)benzo[de]isoquinolino[2,1-a][1,8]naphthyridine (3ae)

The general procedure was followed using **1a** (30.0 mg, 0.2 mmol) and **2e** (225.0 mg, 0.7 mmol) at 35 °C for 12 h. Purification by column chromatography on silica gel (*n*-hexane/EtOAc = 50 : 1) yielded **3ae** (145.0 mg, 67%) as a red solid. M. p. = 219–220°C. **^{1}H NMR** (400 MHz, $CDCl_3$) δ = 7.68 (d, *J* = 7.8 Hz, 2H), 7.51 (d, *J* = 7.5 Hz, 2H), 7.42 – 7.38 (m, 5H), 7.31 (s, 1H), 7.23 – 7.15 (m, 5H), 7.09 – 7.01 (m, 6H), 6.94 (s, 2H), 6.87 (d, *J* = 7.6 Hz, 1H), 6.40 (d, *J* = 8.1 Hz, 2H), 0.37 (s, 9H), 0.28 (s, 9H), 0.26 (s, 9H), 0.18 (s, 9H), 0.14 (s, 9H), 0.10 (s, 9H). **^{13}C NMR** (100 MHz, $CDCl_3$) δ = 150.1 (C_q), 149.6 (C_q), 141.1 (C_q), 140.9 (C_q), 140.2 (C_q), 139.5 (C_q), 138.6 (C_q), 138.5 (C_q), 138.5 (C_q), 138.4 (C_q), 138.2 (C_q), 137.8 (C_q), 137.6 (C_q), 137.6 (C_q), 137.5 (C_q), 135.9 (C_q), 135.6 (C_q), 134.9 (CH), 133.5 (C_q), 133.4 (CH), 132.6 (CH), 132.0 (CH), 131.9 (CH), 131.4 (CH), 131.1 (CH), 131.0 (CH), 130.8 (CH), 130.7 (CH), 130.2 (CH), 129.6 (CH), 129.6 (CH), 128.0 (C_q), 127.6 (CH), 125.9 (C_q), 123.2 (C_q), 122.0 (C_q), 119.1 (CH), 119.0 (C_q), 117.7 (CH), -0.8 (CH_3), -0.9 (CH_3), -0.9 (CH_3), -1.0 (CH_3), -1.1 (CH_3), -1.3 (CH_3). **IR (ATR)**: 2954, 2203, 1548, 1342, 1249, 1108, 840 cm^{-1}. **MS** (ESI) *m/z* (relative intensity): 1081 (98) $[M+H]^+$, 1082 (100) $[M(^{13}C,^{29}Si)+H]^+$. **HR-MS** (ESI) *m/z* calcd for $C_{67}H_{80}N_2Si_6$ $[M+H]^+$: 1081.5010, found: 1081.5017.

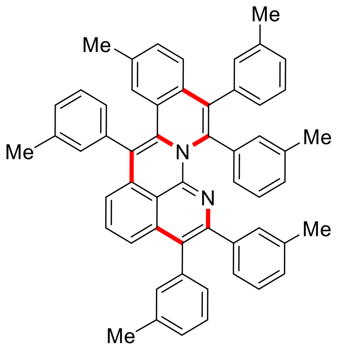

3af

9-Methyl-2,3,7,12,13-penta-m-tolylbenzo[de]isoquinolino[2,1-a][1,8] naphthyridine (3af)

The general procedure was followed using **1a** (30.0 mg, 0.2 mmol) and **2f** (144.0 mg, 0.7 mmol) at 35 °C for 12 h. Purification by column chromatography on silica gel (*n*-hexane/EtOAc = 50 : 1) yielded **3af** (104.0mg, 71%) as a red solid. M. p. = 153–154 °C. ^{1}**H NMR** (400 MHz, $CDCl_3$) δ = 7.51 – 7.44 (m, 2H), 7.42 – 7.33 (m, 3H), 7.29 – 7.19 (m, 3H), 7.11 – 7.03 (m, 7H), 7.00 – 6.91 (m, 6H), 6.88 – 6.82 (m, 3H), 6.47 (s, 2H), 2.47 (s, 3H), 2.35 (s, 3H), 2.34 (s, 3H), 2.18 (s, 3H), 2.12 (s, 3H), 2.00 (s, 3H). ^{13}C NMR (101 MHz, $CDCl_3$) δ = 150.1 (C_q), 149.6 (C_q), 140.7 (C_q), 139.5 (C_q), 139.1 (C_q), 138.1 (C_q), 137.8 (C_q), 137.7 (C_q), 137.3 (C_q), 137.3 (C_q), 137.0 (C_q) 136.7 (C_q), 136.4 ($C_{q,}$ overlapped, 2C), 135.8 (C_q), 135.5 (C_q), 135.3 (C_q), 132.6 (CH), 132.4 ($C_{q,}$ overlapped, 2C), 132.2 (CH), 131.9 (CH), 131.5 (CH), 130.9 (CH), 130.8 (CH), 129.7 (CH), 129.3 (CH), 129.3 (CH), 129.0 (CH), 128.9 (CH), 128.6 (CH), 128.5 (CH), 128.3 (CH), 128.3 (CH), 127.7 (CH), 127.7 (CH), 127.5 (CH), 127.4 (CH), 127.4 (CH), 127.2 (CH), 127.0 (CH), 126.7 (CH), 125.7 (C_q), 125.0 (CH), 123.3 (C_q), 122.0 ($C_{q,}$ overlapped, 2C), 118.9 (CH), 117.5 (CH), 21.6 (CH_3), 21.5 (CH_3), 21.5 (CH_3), 21.4 (CH_3), 21.3 (CH_3), 21.3 (CH_3).. **IR (ATR)**: 3023, 2914, 2091, 1997, 1606, 1538, 1337, 1289, 703 cm^{-1}. **MS** (ESI) *m/z* (relative intensity): 733 (100) $[M+H]^+$, 765 (10) $[M+Na]^+$. **HR-MS** (ESI) *m/z* calcd for $C_{55}H_{45}N_2O$ $[M+H]^+$: 733.3577, found: 733.3578.

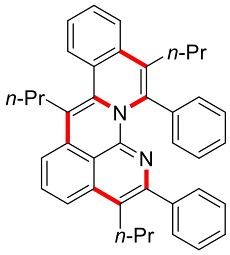

3ag

2,13-Diphenyl-3,7,12-tripropylbenzo[de]isoquinolino[2,1-a][1,8]naphthyridine (3ag)

The general procedure was followed using **1a** (30.0 mg, 0.2 mmol) and **2g** (101.0 mg, 0.7 mmol) at 35 °C for 12 h. Purification by column chromatography on silica gel (*n*-hexane/EtOAc = 100 : 1) yielded two regioisomers **3ag** (55.5 mg) and **3ag'** (20.5 mg) (76.0 mg, 70%, **3ag**: **3ag'** = 2.7 : 1). Characterization data for **3ag**: red solid. M. p. = 175 – 176°C. **^{1}H NMR** (400 MHz, $CDCl_3$) δ = 7.70 (t, J = 8.0 Hz, 2H), 7.54 (dd, J = 8.0, 1.1 Hz, 1H), 7.47 – 7.41 (m, 2H), 7.37 – 7.31 (m, 2H), 7.28 – 7.10 (m, 8H), 6.77 – 6.72 (m, 2H), 3.08 – 2.96 (m, 2H), 2.67 – 2.55 (m, 4H), 1.96 – 1.84 (m, 2H), 1.56 – 1.43 (m, 4H), 1.18 (t, J = 7.3 Hz, 3H), 0.73 (t, J = 7.4 Hz, 3H), 0.69 (t, J = 7.3 Hz, 3H). **^{13}C NMR** (101 MHz, $CDCl_3$) δ = 150.8 (C_q), 147.7 (C_q), 141.5 (C_q), 137.9 (C_q), 137.6 (C_q), 137.4 (C_q), 136.4 (C_q), 134.5 (C_q), 134.2 (C_q), 130.8 (CH), 129.6 (CH, overlapped, 2C), 128.8 (C_q), 128.7 (CH), 127.7 (CH), 127.3 (CH, overlapped, 2C), 127.1 (CH), 126.9 (CH), 126.1 (CH), 123.2 (CH), 122.5 (C_q), 121.0 (C_q), 120.9 (C_q), 117.5 (CH), 116.7 (C_q), 115.3 (CH), 30.9 (CH_2), 30.4 (CH_2), 29.8 (CH_2), 22.8 (CH_2, overlapped, 2C), 21.2 (CH_2), 14.5 (CH_3), 14.1 (CH_3), 13.9 (CH_3). **IR (ATR)**: 3065, 2957, 2866, 1611, 1547, 1334, 1281, 757 cm^{-1}. **MS** (ESI) *m/z* (relative intensity): 547 (100) $[M+H]^+$, 579 (40) $[M+Na]^+$. **HR-MS** (ESI) *m/z* calcd for $C_{40}H_{39}N_2$ $[M+H]^+$: 547.3108, found: 547.3108.

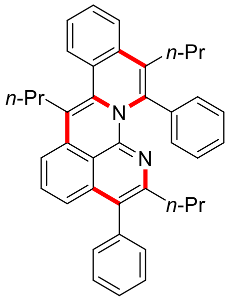

3ag'

3,13-Diphenyl-2,7,12-tripropylbenzo[de]isoquinolino[2,1-a][1,8]naphthyridine (3ag')

Characterization data for **3ag'**: **^{1}H NMR** (400 MHz, $CDCl_3$) δ = 7.74 (t, J = 7.9 Hz, 1H), 7.65 (d, J = 7.5 Hz, 1H), 7.54 (d, J = 8.3 Hz, 1H), 7.49 – 7.37 (m, 6H), 7.41 – 7.30 (m, 5H), 7.27 – 7.15 (m, 2H), 6.75 (dd, J = 7.6, 1.4 Hz, 1H), 3.19 – 3.01 (m, 2H), 3.03 – 2.98 (m, 2H), 2.85 – 2.79 (m, 2H), 1.94 – 1.81 (m, 2H), 1.65 (dq, J = 14.9, 7.3 Hz, 2H), 1.17 (t, J = 7.3 Hz, 3H), 0.87 (t, J = 7.3 Hz, 3H), 0.73 (p, J = 7.4 Hz, 2H), 0.45 (t, J = 7.3 Hz, 3H). **^{13}C NMR** (100 MHz, $CDCl_3$) δ = 151.0 (C_q), 148.0 (C_q), 142.1 (C_q), 139.6 (C_q), 138.8 (C_q), 137.5 (C_q), 136.5 (C_q), 135.5 (C_q), 133.9 (C_q), 131.5 (CH), 131.1 (CH), 129.4 (CH), 128.6 (CH), 128.6 (CH), 128.1 (CH), 127.5 (C_q), 127.4 (CH), 127.2 (CH), 126.7 (CH), 125.5 (CH), 124.9 (CH), 123.4 (C_q), 122.1 (C_q), 122.1 (C_q), 117.6 (CH), 117.6 (C_q), 115.7 (CH), 31.0 (CH_2), 30.7 (CH_2), 30.7 (CH_2), 23.1 (CH_2), 21.3 (CH_2), 20.0 (CH_2), 14.5 (CH_3), 14.3 (CH_3), 13.5 (CH_3). **IR (ATR)**: 3063, 2959, 2870, 1614, 1549, 1340, 1257, 767 cm^{-1}. **MS** (ESI) m/z (relative intensity): 547 (100) $[M+H]^+$, 579 (20) $[M+Na]^+$. **HR-MS** (ESI) m/z calcd for $C_{40}H_{39}N_2$ $[M+H]^+$: 547.3108, found: 547.3110.

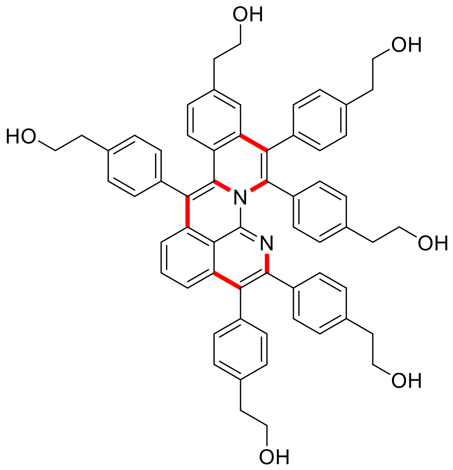

3ah

2,2',2'',2''',2''''-((10-(2-Hydroxyethyl)benzo[de]isoquinolino[2,1-a][1,8] naphthyridine-2,3,7,12,13-pentayl)pentakis(benzene-4,1-diyl))pentakis(ethan-1-ol) (3ah)

The general procedure was followed using **1a** (30.0 mg, 0.2 mmol) and **2h** (186.4 mg, 0.7 mmol) at 35 °C for 12 h. Purification by column chromatography on silica gel (CH_2Cl_2/MeOH = 30 : 1) yielded **3ah** (128.1 mg, 70%) as a red solid. M. p. = 248–250 °C. **^{1}H NMR** (400 MHz, CD_3OD) δ = 7.34 – 7.26 (m, 3H), 7.25 – 7.17 (m, 2H), 7.14 – 7.04 (m, 6H), 7.01 – 6.86 (m, 6H), 6.83 – 6.72 (m, 7H), 6.39 (d, J = 8.1 Hz, 2H), 3.81 – 3.71 (m, 6H), 3.66 – 3.53 (m, 6H), 2.90 – 2.76 (m, 6H), 7.59 – 2.67 (m, 6H). **^{13}C NMR** (100 MHz, CD_3OD) δ = 149.7 (C_q), 139.8 (C_q), 139.0 (C_q), 138.1 (C_q), 137.7 (C_q), 137.6 (C_q), 137.5 (C_q, overlapped, 3C), 137.4 (C_q), 137.1 (C_q), 136.0 (C_q), 135.3 (C_q), 135.2 (C_q), 135.1 (C_q), 134.8 (C_q), 134.7 (C_q), 134.4 (C_q), 131.7 (CH), 131.2 (CH), 131.0 (CH, overlapped, 2C), 130.4 (CH), 130.3 (CH, overlapped, 2C), 128.8 (CH), 128.4 (CH), 127.6 (CH), 127.2 (CH, overlapped, 2C), 126.8 (CH), 125.8 (C_q), 125.7 (C_q), 125.6 (CH), 123.2 (C_q), 121.5 (C_q), 118.9 (CH), 118.6 (Cq), 117.9 (CH), 63.0 (CH_2), 62.8 (CH_2), 62.7 (CH_2), 62.6 (CH_2, overlapped, 2C), 62.1 (CH_2), 38.6 (CH_2, overlapped, 3C), 38.47 (CH_2, overlapped, 3C). **IR** (ATR): 3292 2927, 2863, 1606, 1506, 1337, 1043, 818 cm^{-1}. **HR-MS** (ESI) *m/z* calcd for $C_{61}H_{57}N_2O_6$ $[M+H]^+$: 913.4211, found: 913.4186.

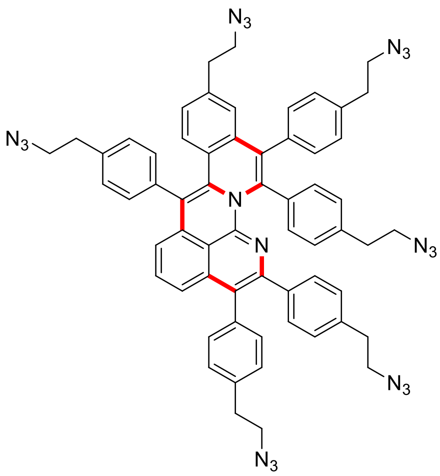

3ai

10-(2-Azidoethyl)-2,3,7,12,13-pentakis(4-(2-azidoethyl)phenyl) benzo[de]isoquinolino[2,1-a][1,8]naphthyridine (3ai)

The general procedure was followed using **1a** (30.0 mg, 0.2 mmol) and **2i** (221.5 mg, 0.7 mmol) at 35 °C for 12 h. Purification by column chromatography on silica gel (*n*-hexane/EtOAc = 50 : 1) yielded **3ai** (89.3 mg, 42%) as a red solid. M. p. = 99–100 °C. **^{1}H NMR** (400 MHz, $CDCl_3$) δ = 7.52 – 7.38 (m, 5H), 7.24 – 7.16 (m, 7H), 7.10 – 7.03 (m, 4H), 6.99 – 6.94 (m, 2H), 6.91 – 6.87 (m, 3H), 6.80 (d, *J* = 8.1 Hz, 2H), 6.74 – 6.67 (m, 1H), 6.51 (d, *J* = 8.1 Hz, 2H). 3.64 (t, *J* = 6.9 Hz, 2H), 3.54 (t, *J* = 6.9 Hz, 2H), 3.51 (t, *J* = 6.9 Hz, 2H), 3.40 – 3.32 (m, 6H), 3.05 (t, *J* = 6.9 Hz, 2H), 2.94 (t, *J* = 6.6 Hz, 2H), 2.91 (t, *J* = 7.1 Hz, 2H), 2.80 – 2.69 (m, 6H). **^{13}C NMR** (100 MHz, $CDCl_3$) δ = 149.6 (C_q), 149.4 (C_q), 139.2 (C_q), 138.4 (C_q), 138.0 (C_q), 137.7 (C_q), 137.5 (C_q), 137.2 (C_q), 136.7 ($C_{q,}$ overlapped, 2C), 136.6 (C_q), 136.40 (C_q, overlapped, 3C), 135.6 (C_q), 135.5 (C_q), 135.4 (C_q), 135.3 (C_q), 134.9 (C_q), 132.1 (CH, overlapped, 2C), 131.5 (CH), 131.1 (CH), 130.5 (CH, overlapped, 2C), 130.4 (CH), 129.0 (CH), 128.9 (CH), 128.6 (CH), 127. 5 (CH), 127.3 (CH), 126.6 (CH), 126.2 (C_q), 125.0 (CH), 122.8 (C_q), 121.8 (C_q), 119.0 (CH), 118.4 (C_q), 117.6 (CH), 52.5 (CH_2, overlapped, 3C), 52.4 (CH_2, overlapped, 2C), 51.9 (CH_2), 35.5 (CH_2), 35.4 (CH_2), 35.3 (CH_2), 35.1 (CH_2), 35.0 (CH_2, overlapped, 2C). **IR** (ATR): 2924, 2085, 1606, 1508, 1337, 1252, 825, 773 cm^{-1}. **HR-MS** (ESI) *m/z* calcd for $C_{61}H_{51}N_{20}$ $[M+H]^+$: 1063.4594, found: 1063.4600.

11. References

[1] J. D. Williams, S. T. Nguyen, S. Gu, X. Ding, M. M. Butler, T. F. Tashjian, T. J. Opperman, R. G. Panchal, S. Bavari, N. P. Peet, D. T. Moir, T. L. Bowlin, *Bioorg. Med. Chem.* **2013**, *21*, 7790–7806.

[2] J. E. Johnson, S. C. Cornell, *J. Org. Chem.* **1980**, *45*, 4144–4148.

[3] W.-J. Kong, L. H. Finger, A. M. Messinis, R. Kuniyil, J. C. A. Oliveira, L. Ackermann, *J. Am. Chem. Soc.* **2019**, *141*, 17198–17206.

[4] J. Jayakumar, K. Parthasarathy, Y. H. Chen, T. H. Lee, S. C. Chuang, C. H. Cheng, *Angew. Chem. Int. Ed.* **2014**, *53*, 9889–9892.

12. NMR Spectra

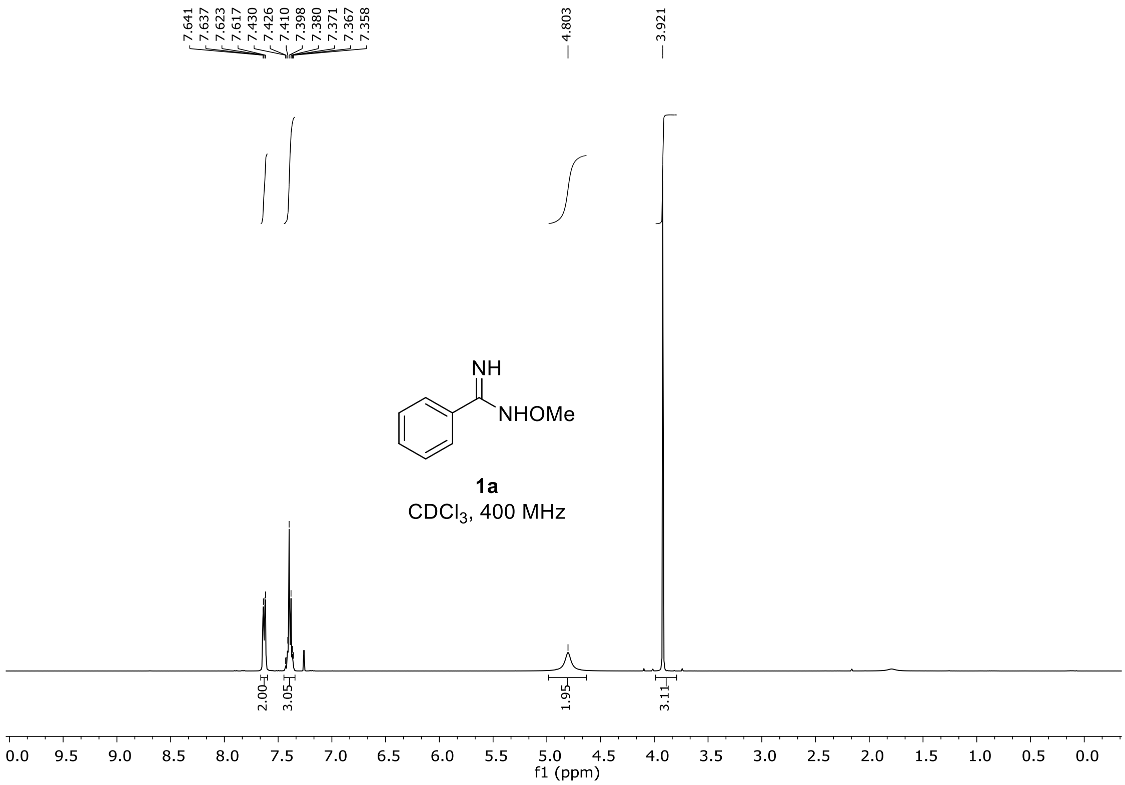

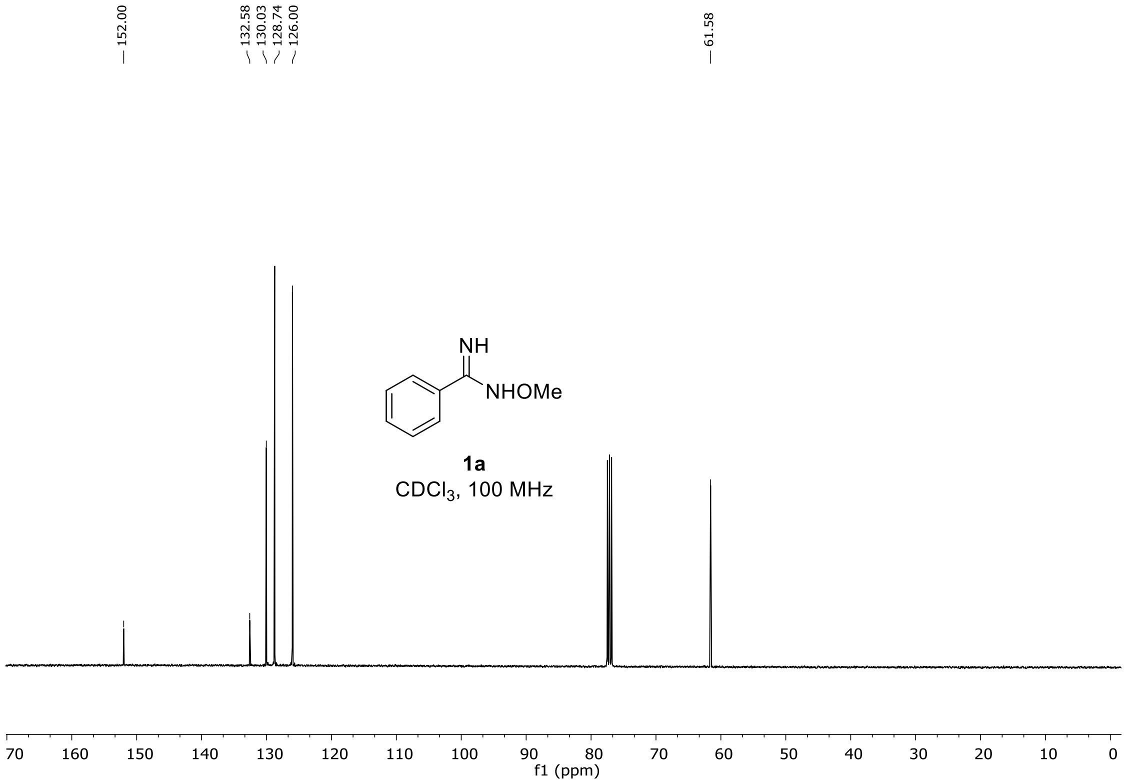

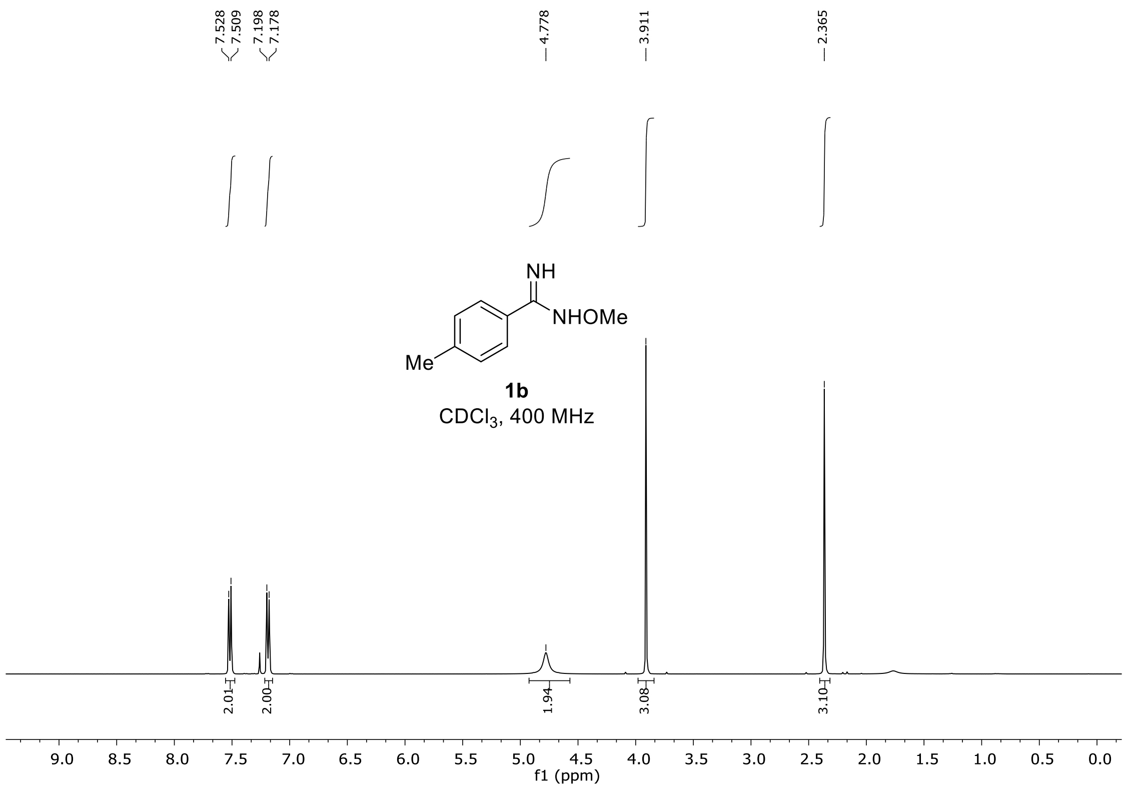
7.528
7.509
7.198
7.178
4.778
3.911
2.365
NH
NHOMe
Me
1b
CDCl3, 400 MHz
2.01
2.00
1.94
3.08
3.10
9.0 8.5 8.0 7.5 7.0 6.5 6.0 5.5 5.0 4.5 4.0 3.5 3.0 2.5 2.0 1.5 1.0 0.5 0.0
f1 (ppm)

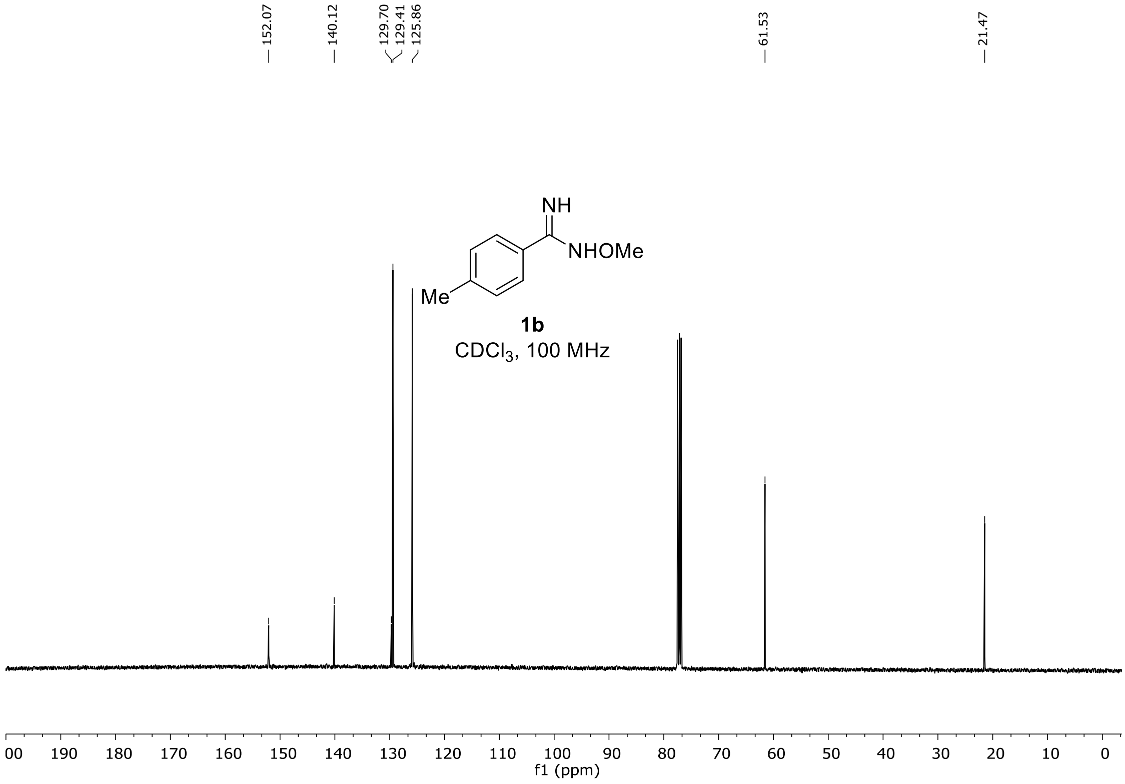
152.07
140.12
129.70
129.41
125.86
61.53
21.47
NH
NHOMe
Me
1b
CDCl3, 100 MHz
00 190 180 170 160 150 140 130 120 110 100 90 80 70 60 50 40 30 20 10 0
f1 (ppm)

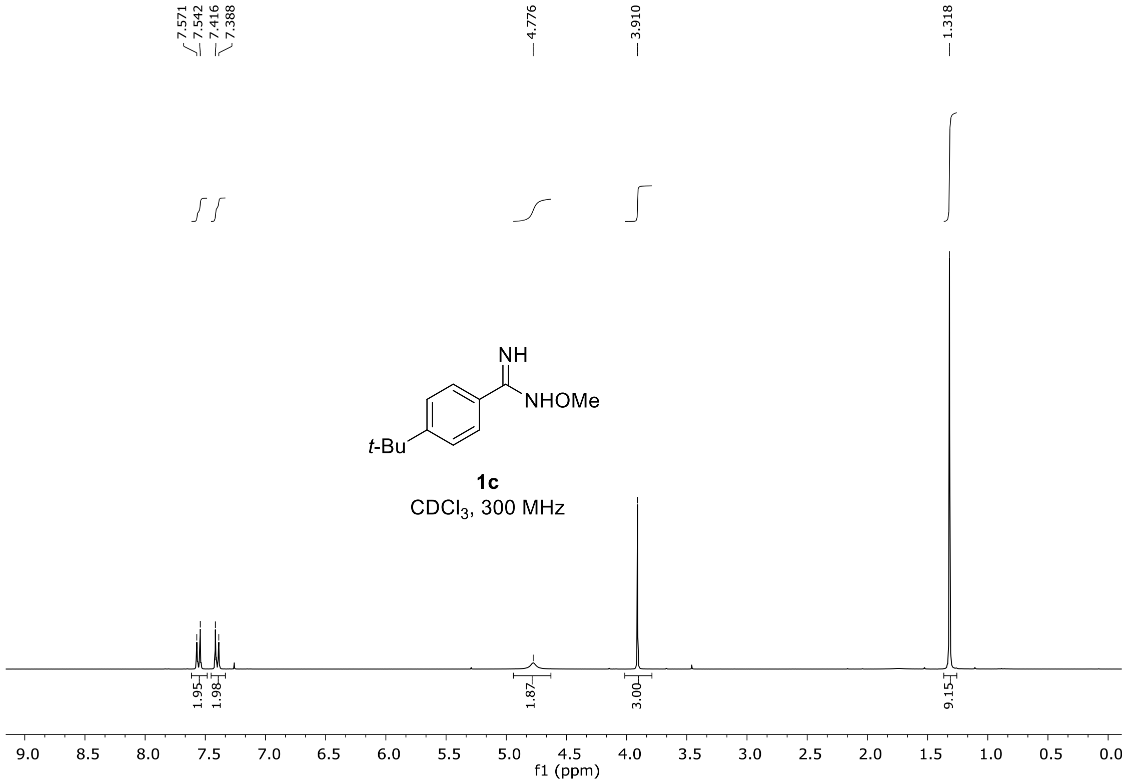
7.571
7.542
7.416
7.388
4.776
3.910
1.318
NH
NHOMe
t-Bu
1c
CDCl3, 300 MHz
1.95
1.98
1.87
3.00
9.15
9.0 8.5 8.0 7.5 7.0 6.5 6.0 5.5 5.0 4.5 4.0 3.5 3.0 2.5 2.0 1.5 1.0 0.5 0.0
f1 (ppm)

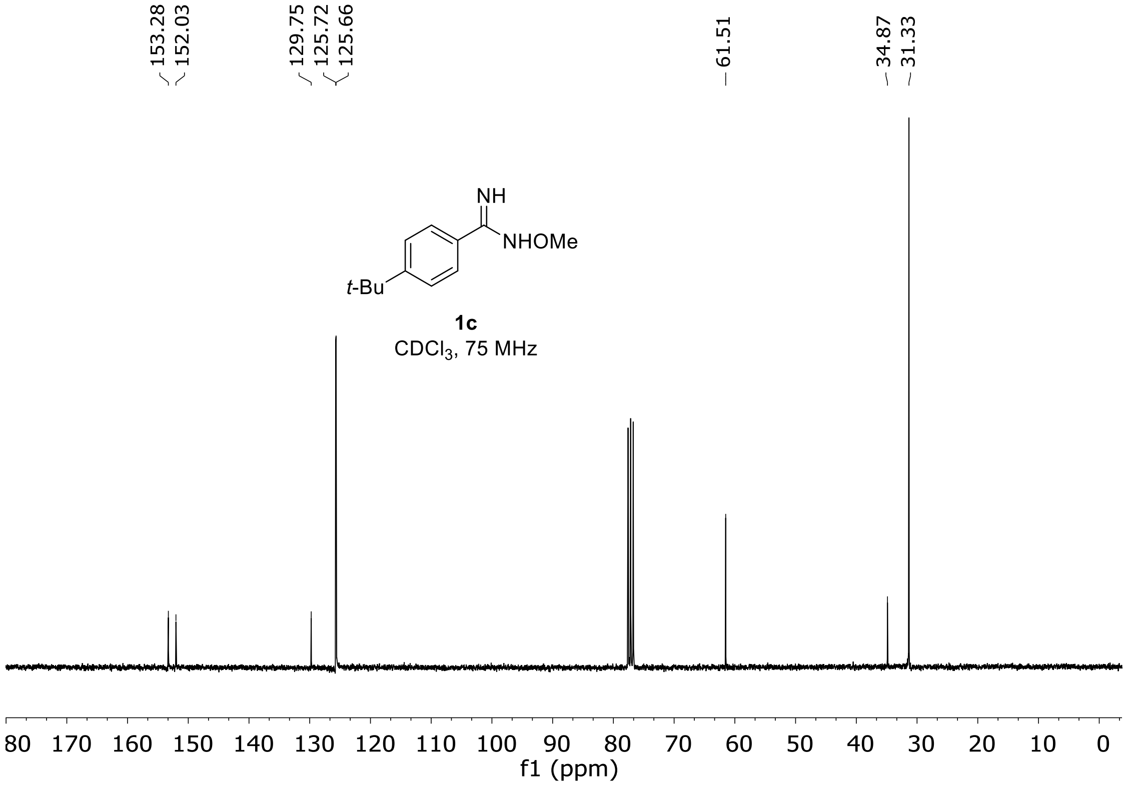
153.28
152.03
129.75
125.72
125.66
61.51
34.87
31.33
NH
NHOMe
t-Bu
1c
CDCl3, 75 MHz
80 170 160 150 140 130 120 110 100 90 80 70 60 50 40 30 20 10 0
f1 (ppm)

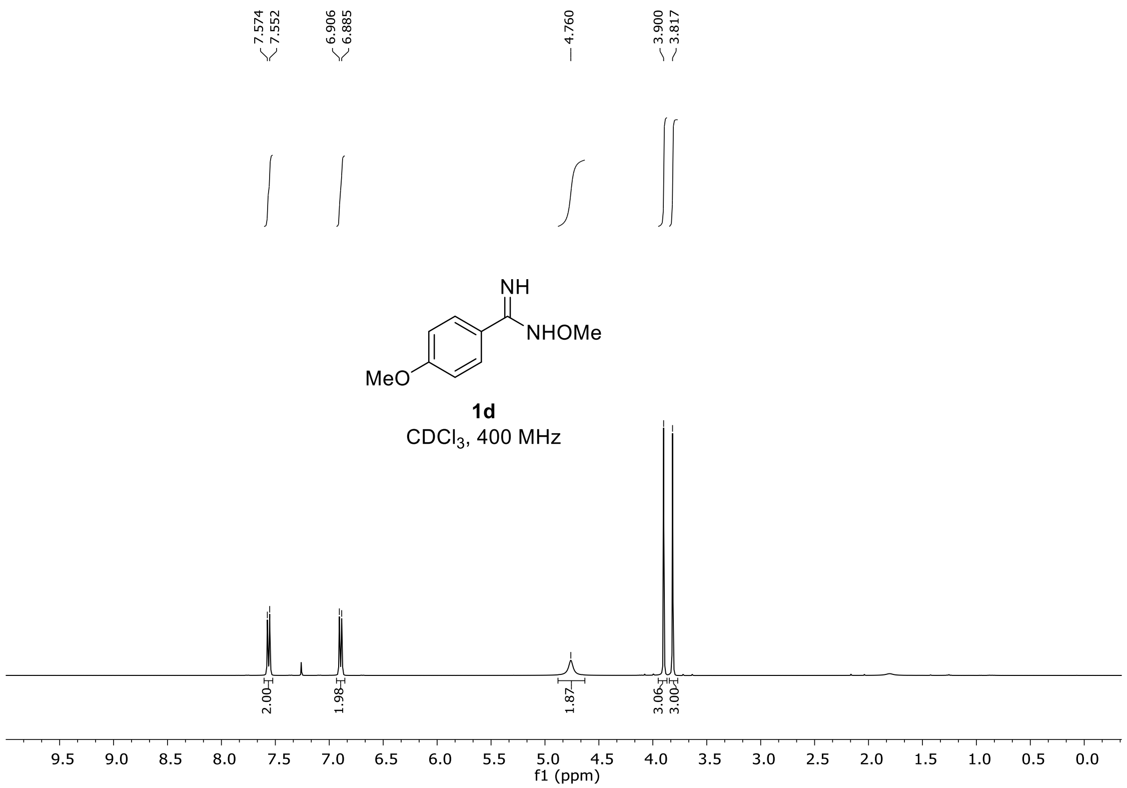
7.574
7.552
6.906
6.885
4.760
3.900
3.817
NH
NHOMe
MeO
1d
CDCl3, 400 MHz
2.00
1.98
1.87
3.06
3.00
9.5 9.0 8.5 8.0 7.5 7.0 6.5 6.0 5.5 5.0 4.5 4.0 3.5 3.0 2.5 2.0 1.5 1.0 0.5 0.0
f1 (ppm)

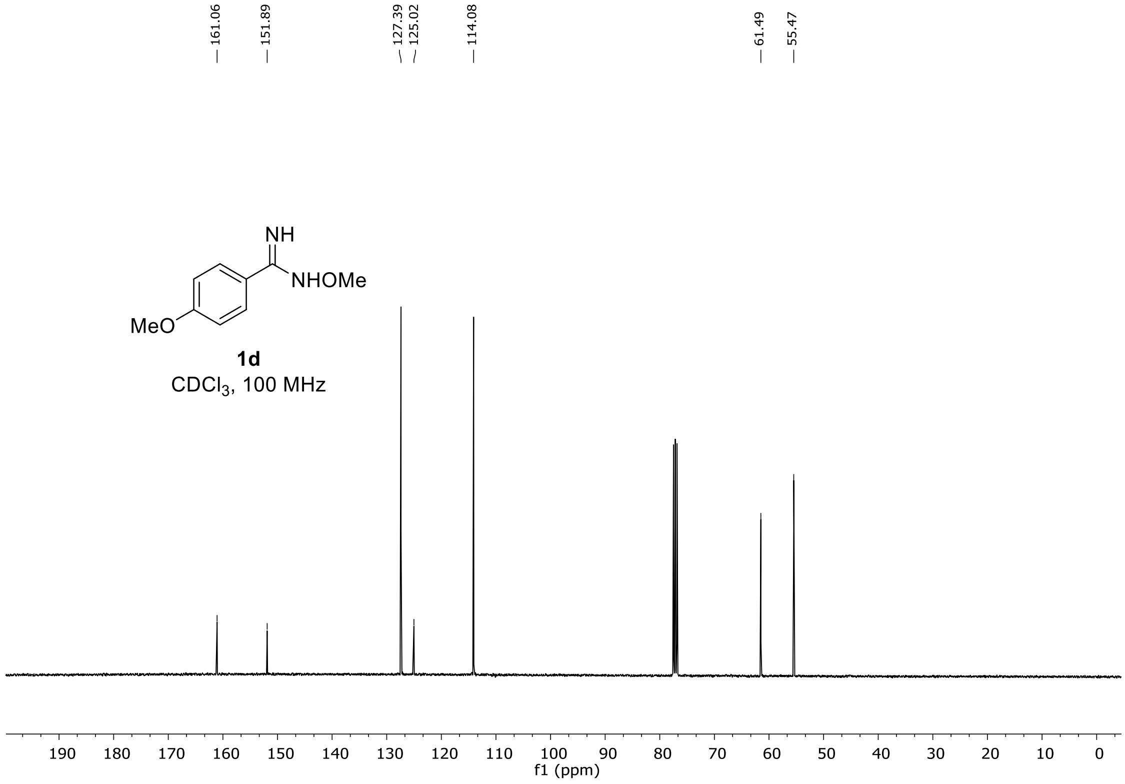
161.06
151.89
127.39
125.02
114.08
61.49
55.47
NH
NHOMe
MeO
1d
CDCl3, 100 MHz
190 180 170 160 150 140 130 120 110 100 90 80 70 60 50 40 30 20 10 0
f1 (ppm)

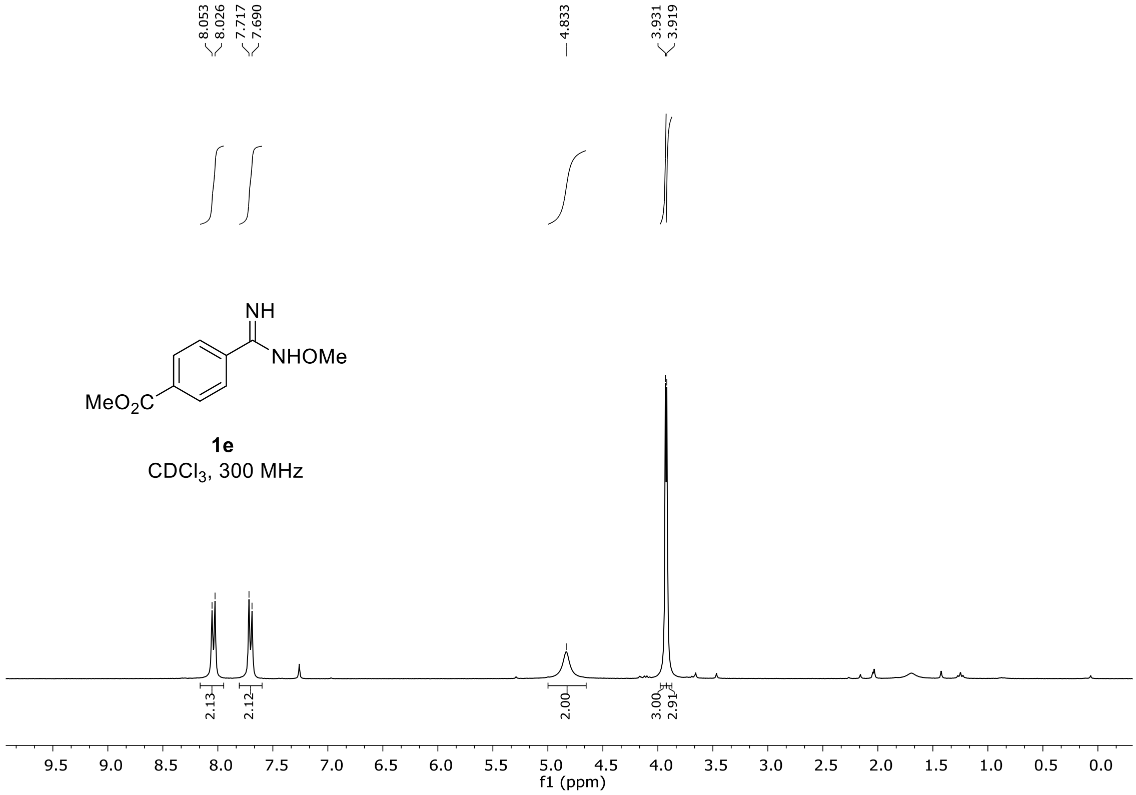
8.053
8.026
7.717
7.690
4.833
3.931
3.919
NH
NHOMe
MeO_2C
1e
$CDCl_3$, 300 MHz
2.13
2.12
2.00
3.00
2.91
9.5 9.0 8.5 8.0 7.5 7.0 6.5 6.0 5.5 5.0 4.5 4.0 3.5 3.0 2.5 2.0 1.5 1.0 0.5 0.0
f1 (ppm)

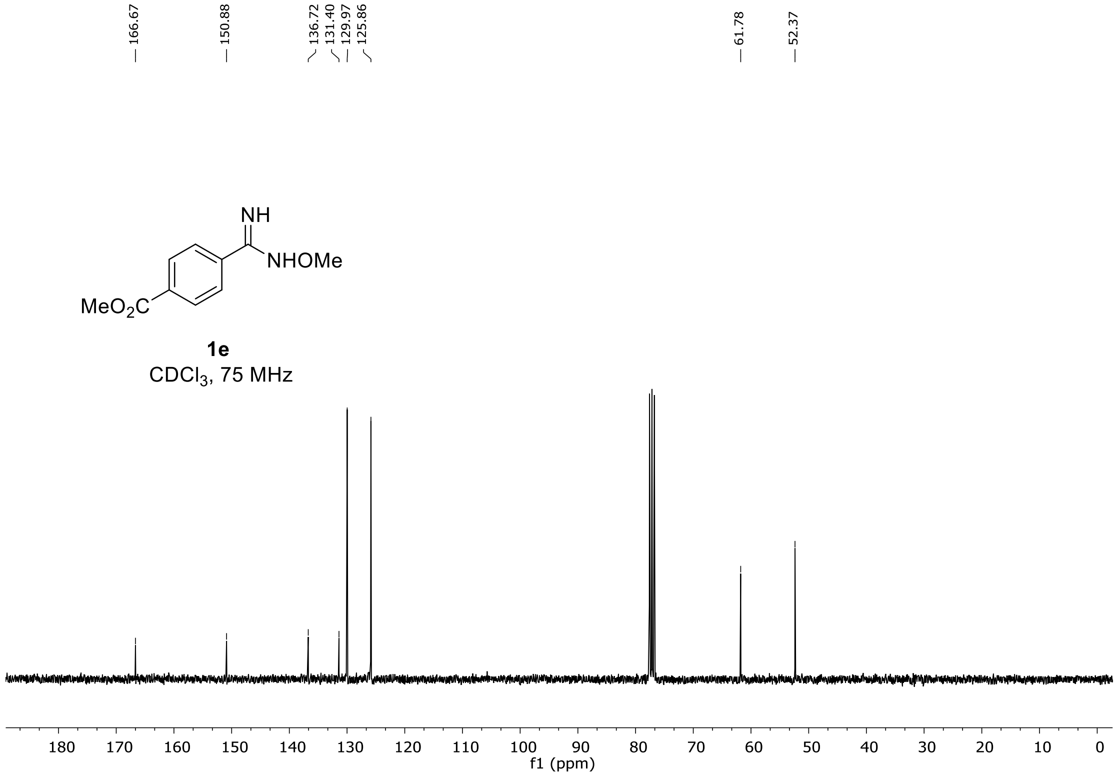
166.67
150.88
136.72
131.40
129.97
125.86
61.78
52.37
NH
NHOMe
MeO_2C
1e
$CDCl_3$, 75 MHz
180 170 160 150 140 130 120 110 100 90 80 70 60 50 40 30 20 10 0
f1 (ppm)

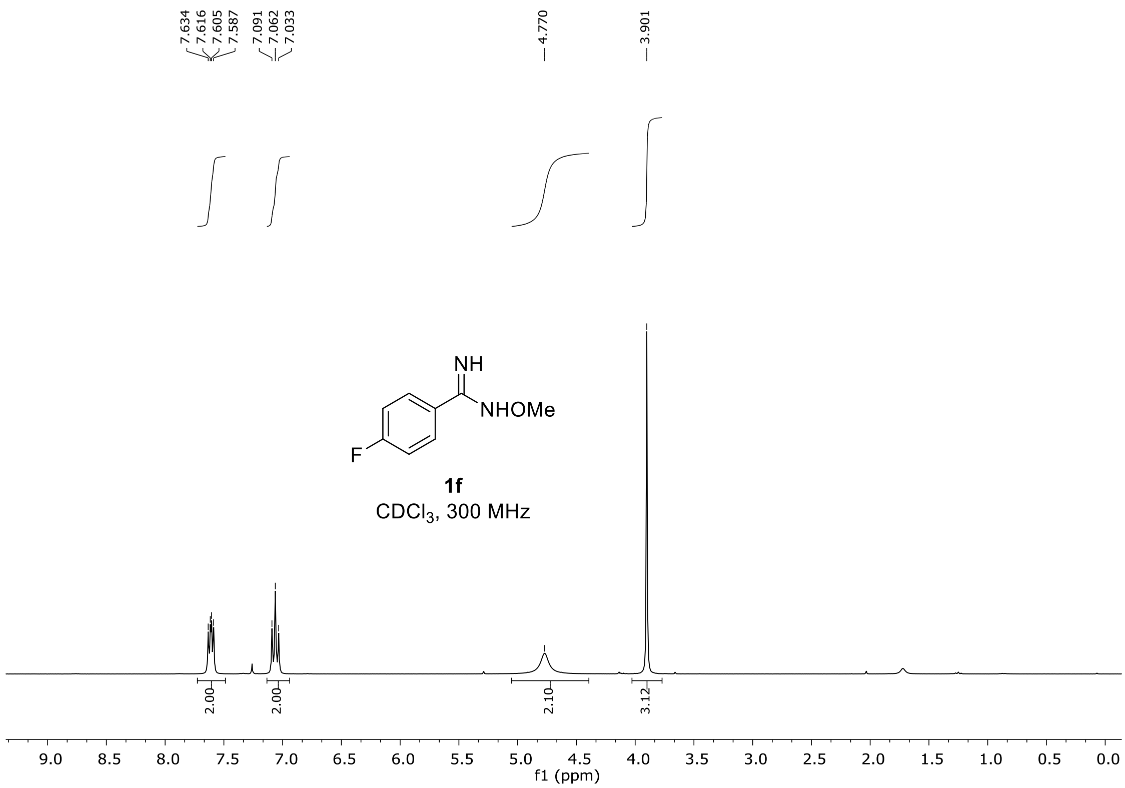
7.634
7.616
7.605
7.587
7.091
7.062
7.033
4.770
3.901
NH
NHOMe
F
1f
CDCl$_3$, 300 MHz
2.00
2.00
2.10
3.12
f1 (ppm)

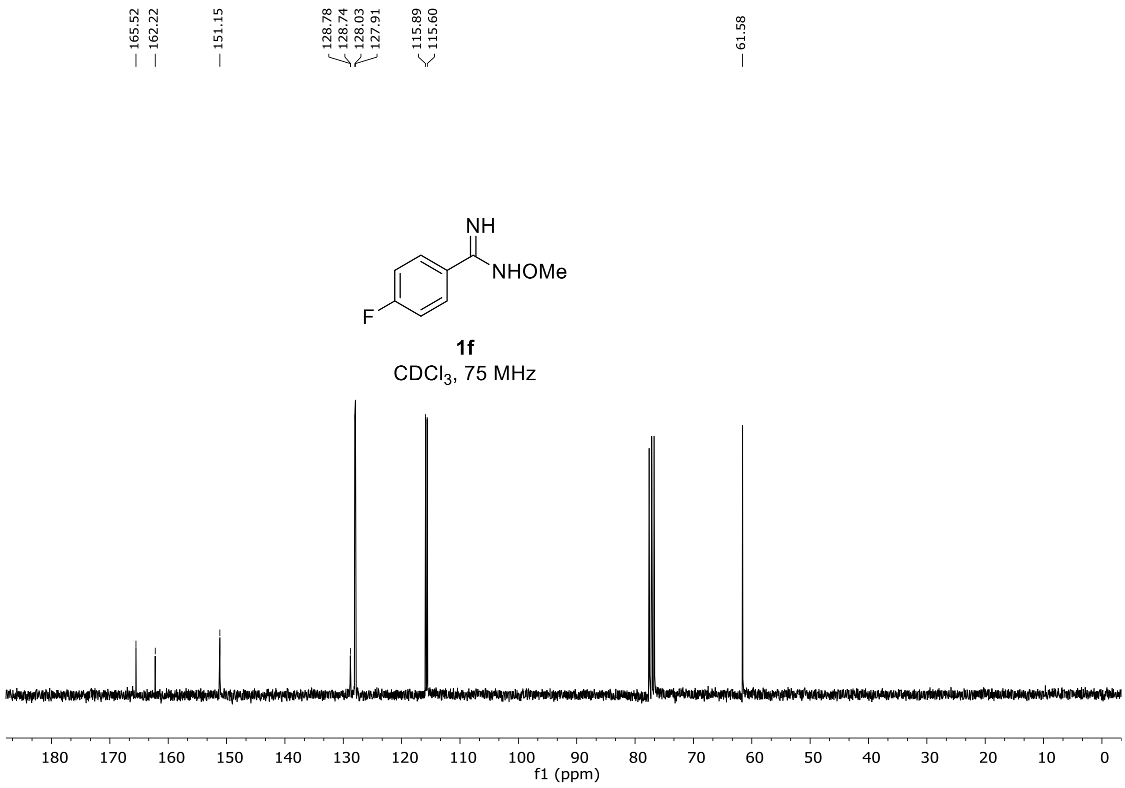
165.52
162.22
151.15
128.78
128.74
128.03
127.91
115.89
115.60
61.58
NH
NHOMe
F
1f
CDCl$_3$, 75 MHz
f1 (ppm)

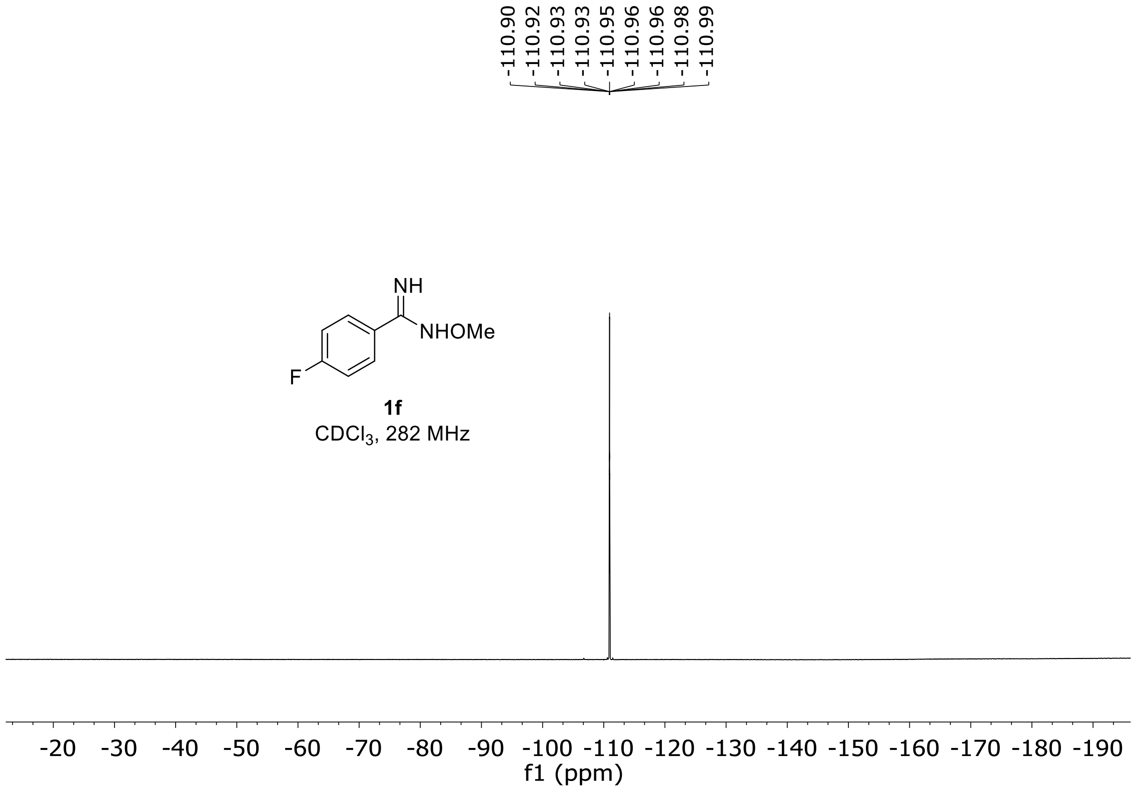

-110.90
-110.92
-110.93
-110.93
-110.95
-110.96
-110.96
-110.98
-110.99
NH
NHOMe
F
1f
CDCl3, 282 MHz
-20 -30 -40 -50 -60 -70 -80 -90 -100 -110 -120 -130 -140 -150 -160 -170 -180 -190
f1 (ppm)

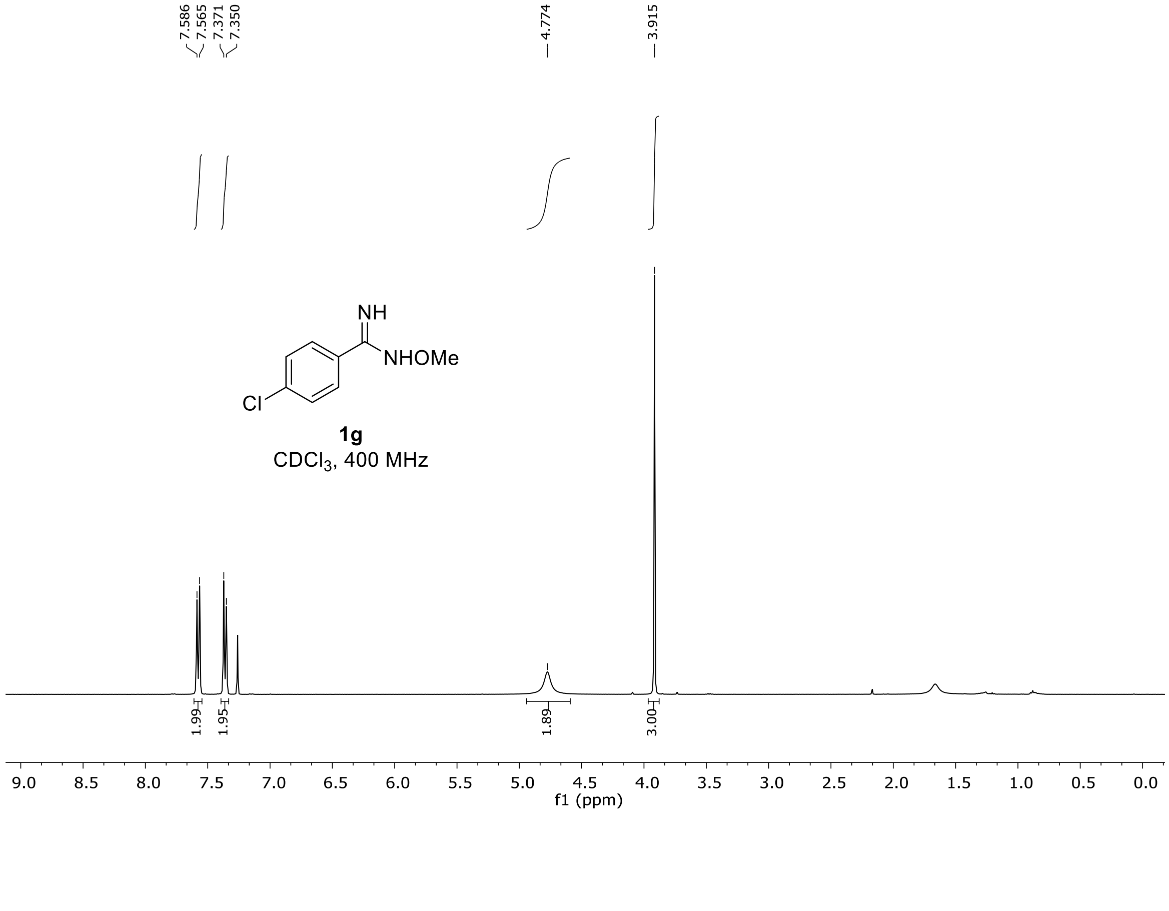

7.586
7.565
7.371
7.350
4.774
3.915
NH
NHOMe
Cl
1g
CDCl3, 400 MHz
1.99
1.95
1.89
3.00
9.0 8.5 8.0 7.5 7.0 6.5 6.0 5.5 5.0 4.5 4.0 3.5 3.0 2.5 2.0 1.5 1.0 0.5 0.0
f1 (ppm)

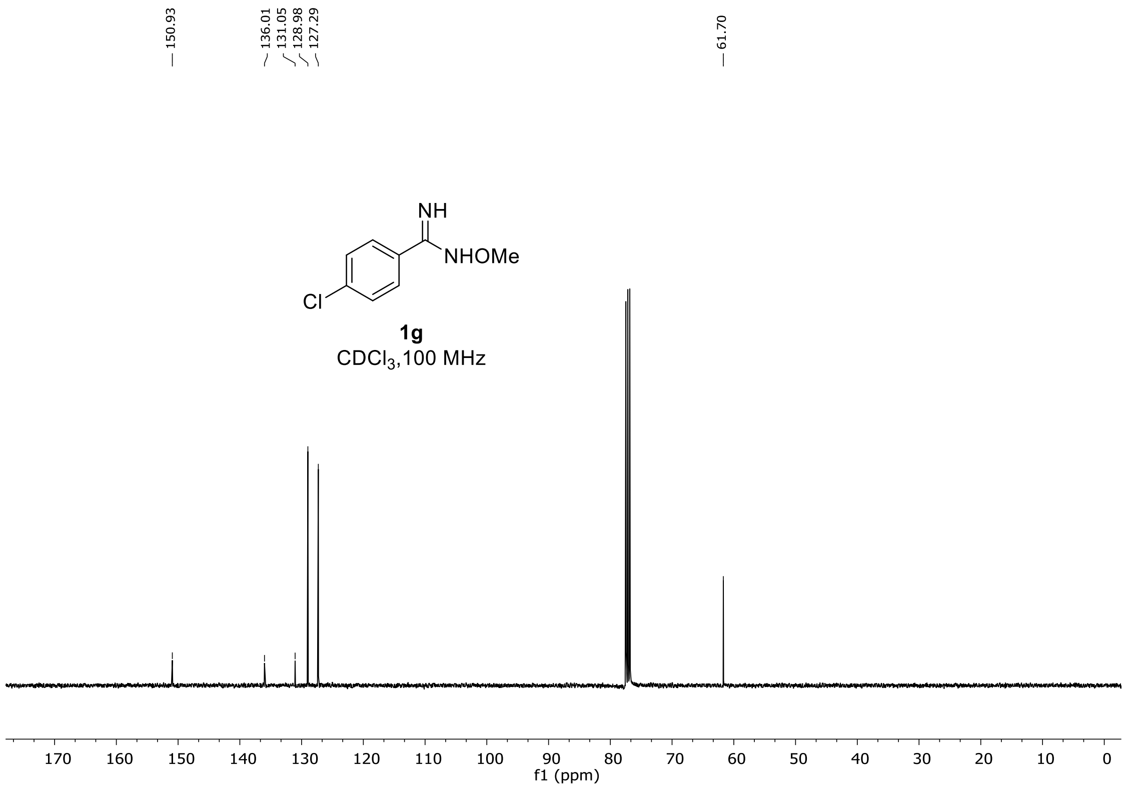
150.93
136.01
131.05
128.98
127.29
61.70
NH
NHOMe
Cl
1g
CDCl3,100 MHz
170 160 150 140 130 120 110 100 90 80 70 60 50 40 30 20 10 0
f1 (ppm)

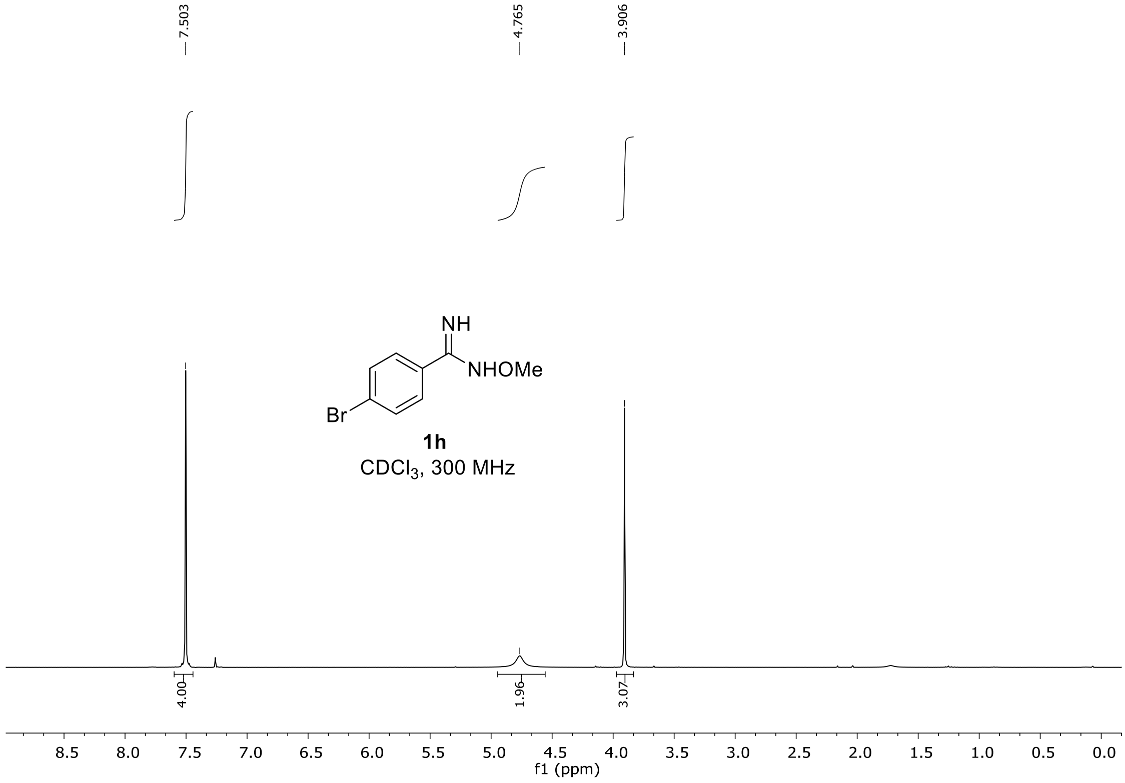
7.503
4.765
3.906
NH
NHOMe
Br
1h
CDCl3, 300 MHz
4.00
1.96
3.07
8.5 8.0 7.5 7.0 6.5 6.0 5.5 5.0 4.5 4.0 3.5 3.0 2.5 2.0 1.5 1.0 0.5 0.0
f1 (ppm)

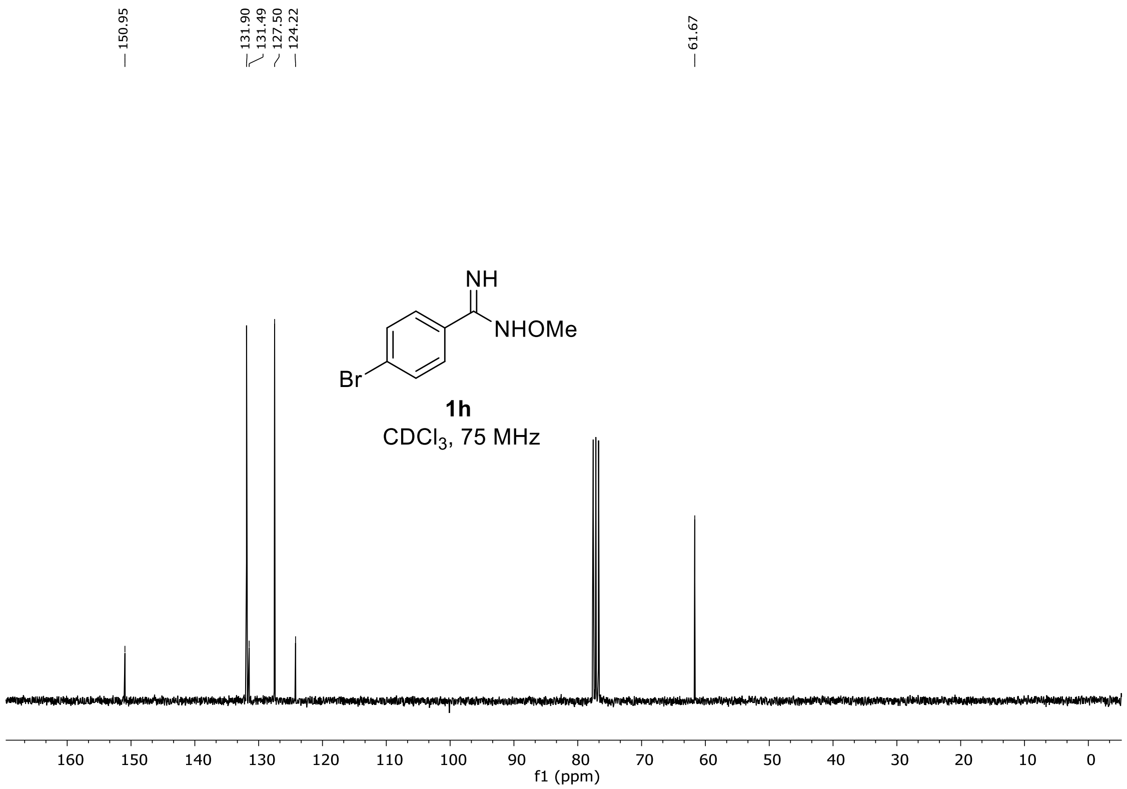
150.95
131.90
131.49
127.50
124.22
61.67
NH
NHOMe
Br
1h
CDCl3, 75 MHz
f1 (ppm)

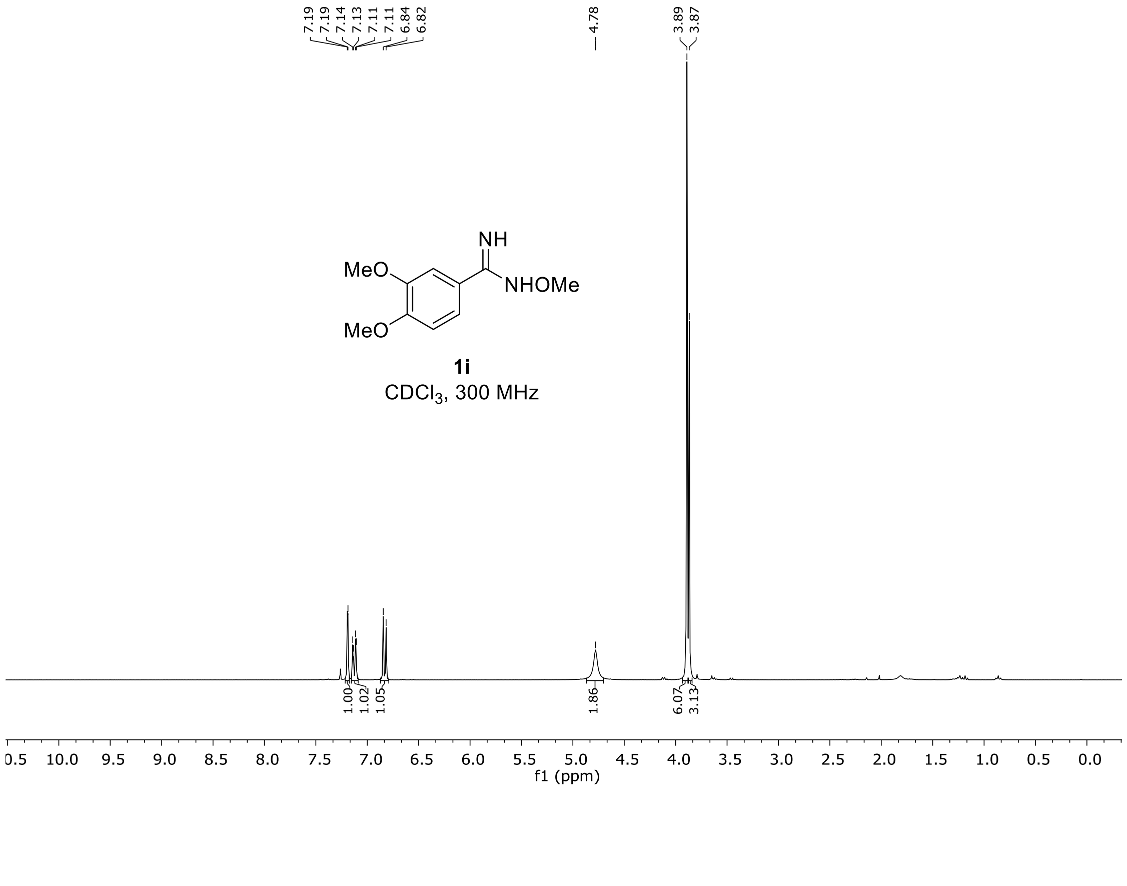
7.19
7.19
7.14
7.13
7.11
7.11
6.84
6.82
4.78
3.89
3.87
NH
MeO
NHOMe
MeO
1i
CDCl3, 300 MHz
1.00
1.02
1.05
1.86
6.07
3.13
f1 (ppm)

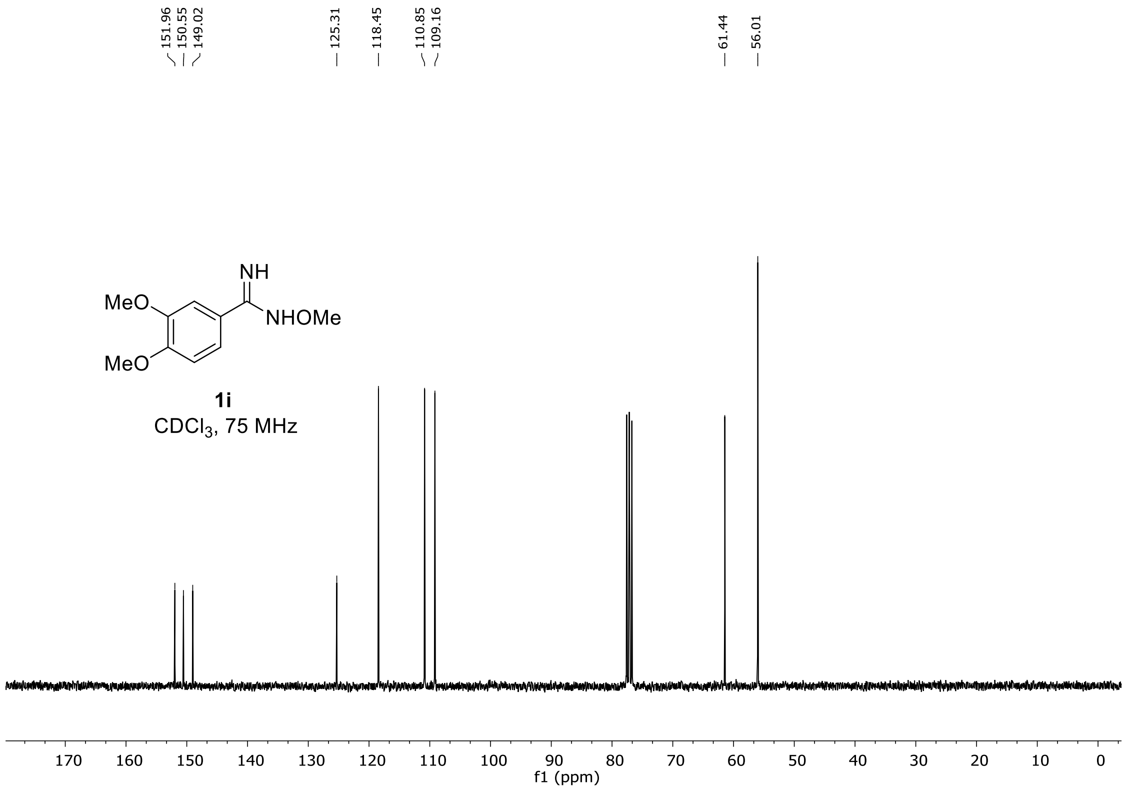
151.96
150.55
149.02
125.31
118.45
110.85
109.16
61.44
56.01
NH
MeO
NHOMe
MeO
1i
CDCl3, 75 MHz
170 160 150 140 130 120 110 100 90 80 70 60 50 40 30 20 10 0
f1 (ppm)

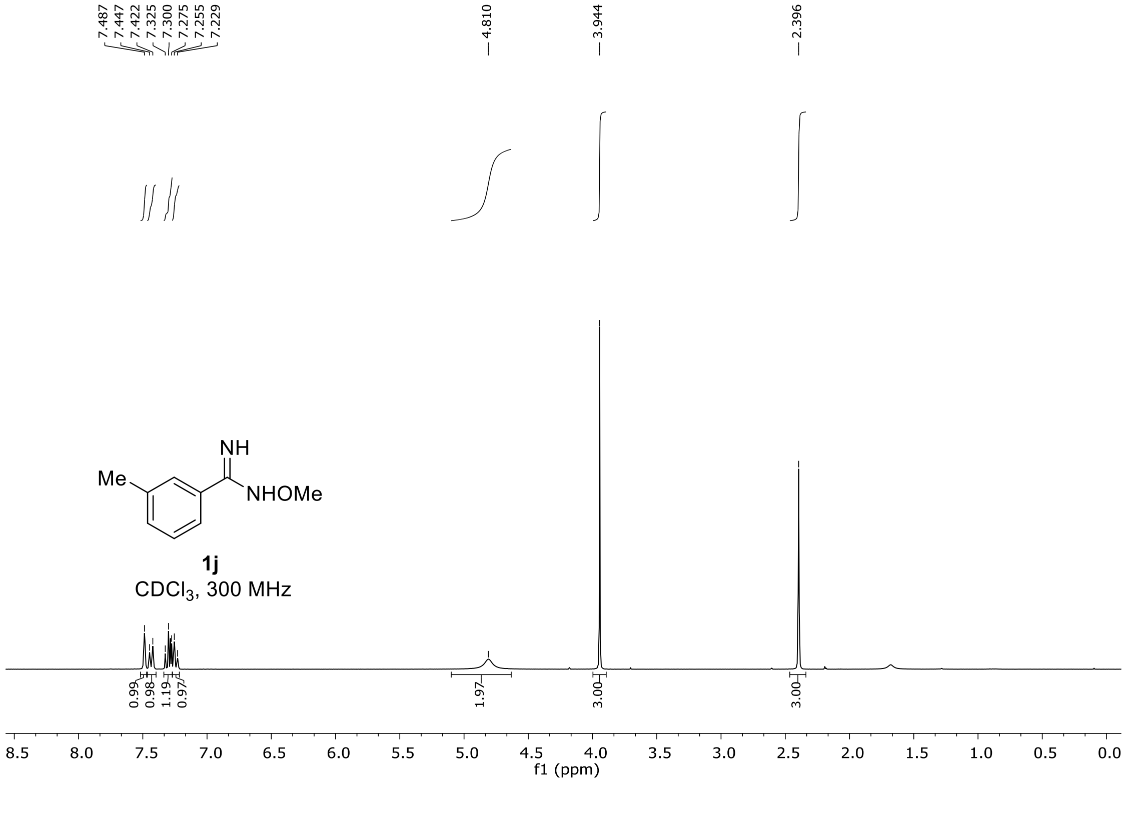
7.487
7.447
7.422
7.325
7.300
7.275
7.255
7.229
4.810
3.944
2.396
NH
Me
NHOMe
1j
CDCl3, 300 MHz
0.99
0.98
1.19
0.97
1.97
3.00
3.00
8.5 8.0 7.5 7.0 6.5 6.0 5.5 5.0 4.5 4.0 3.5 3.0 2.5 2.0 1.5 1.0 0.5 0.0
f1 (ppm)

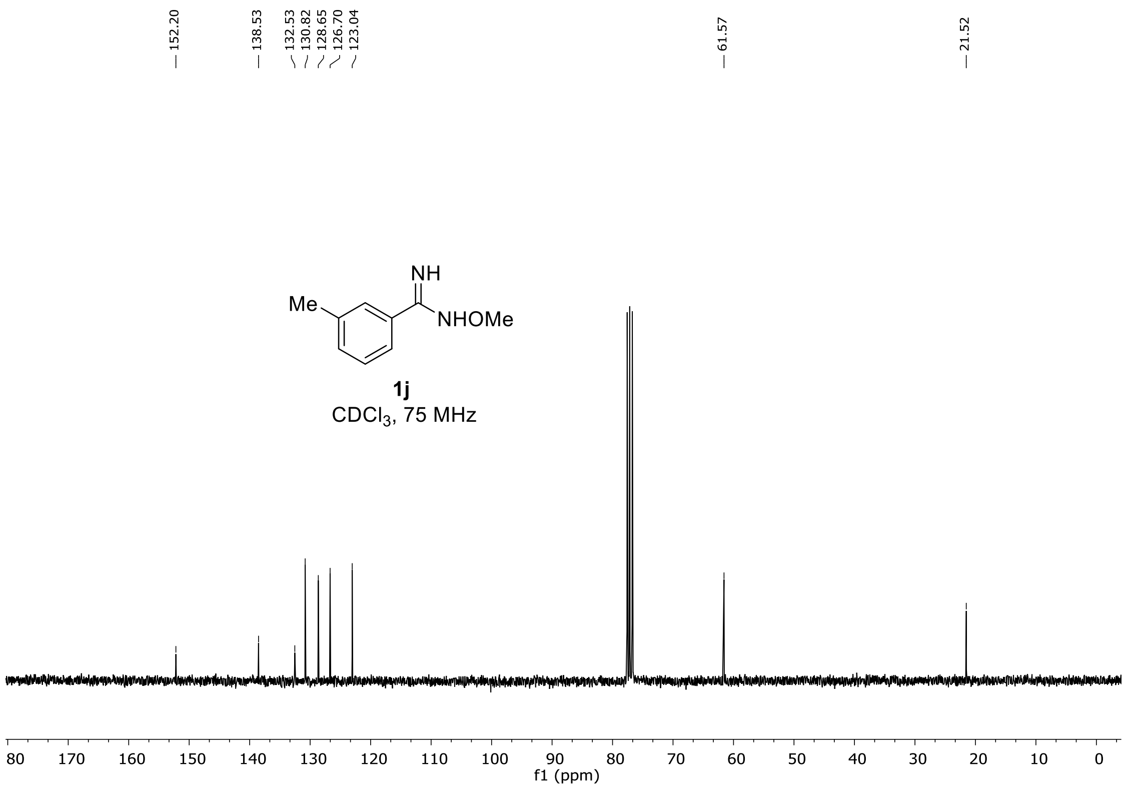
152.20
138.53
132.53
130.82
128.65
126.70
123.04
61.57
21.52
NH
Me
NHOMe
1j
CDCl3, 75 MHz
80 170 160 150 140 130 120 110 100 90 80 70 60 50 40 30 20 10 0
f1 (ppm)

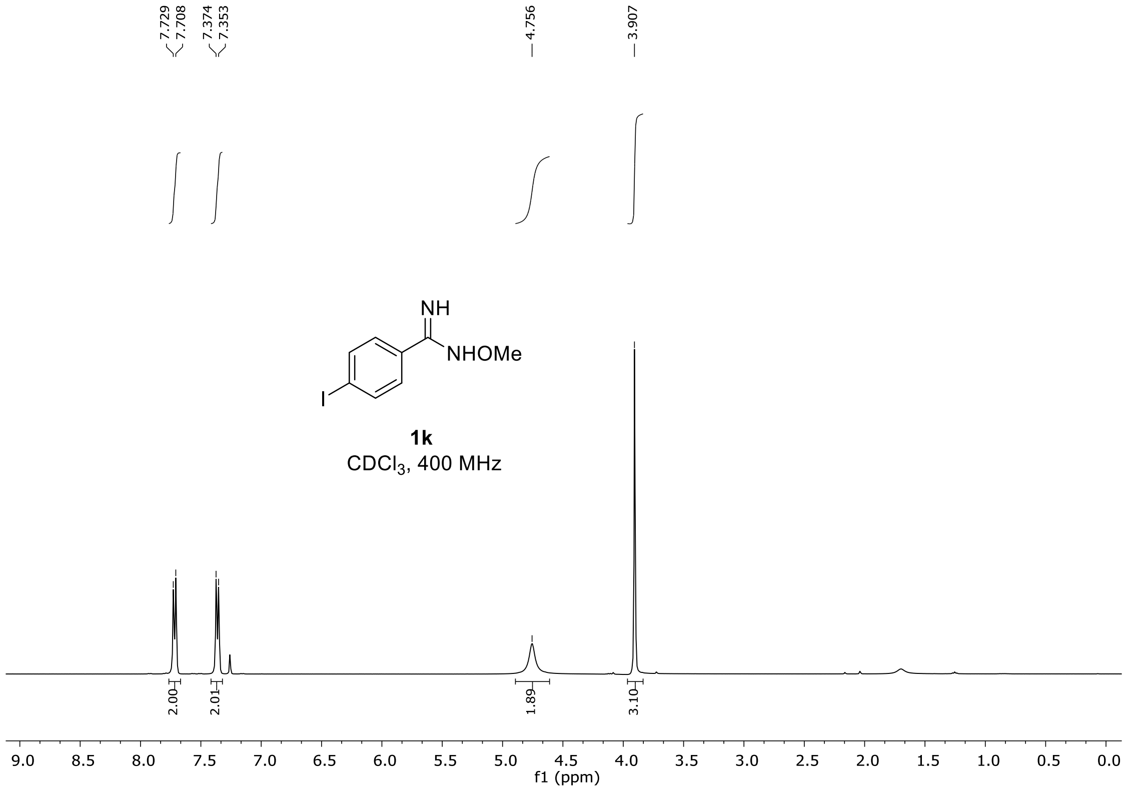
7.729
7.708
7.374
7.353
4.756
3.907
NH
NHOMe
I
1k
CDCl3, 400 MHz
2.00
2.01
1.89
3.10
9.0 8.5 8.0 7.5 7.0 6.5 6.0 5.5 5.0 4.5 4.0 3.5 3.0 2.5 2.0 1.5 1.0 0.5 0.0
f1 (ppm)

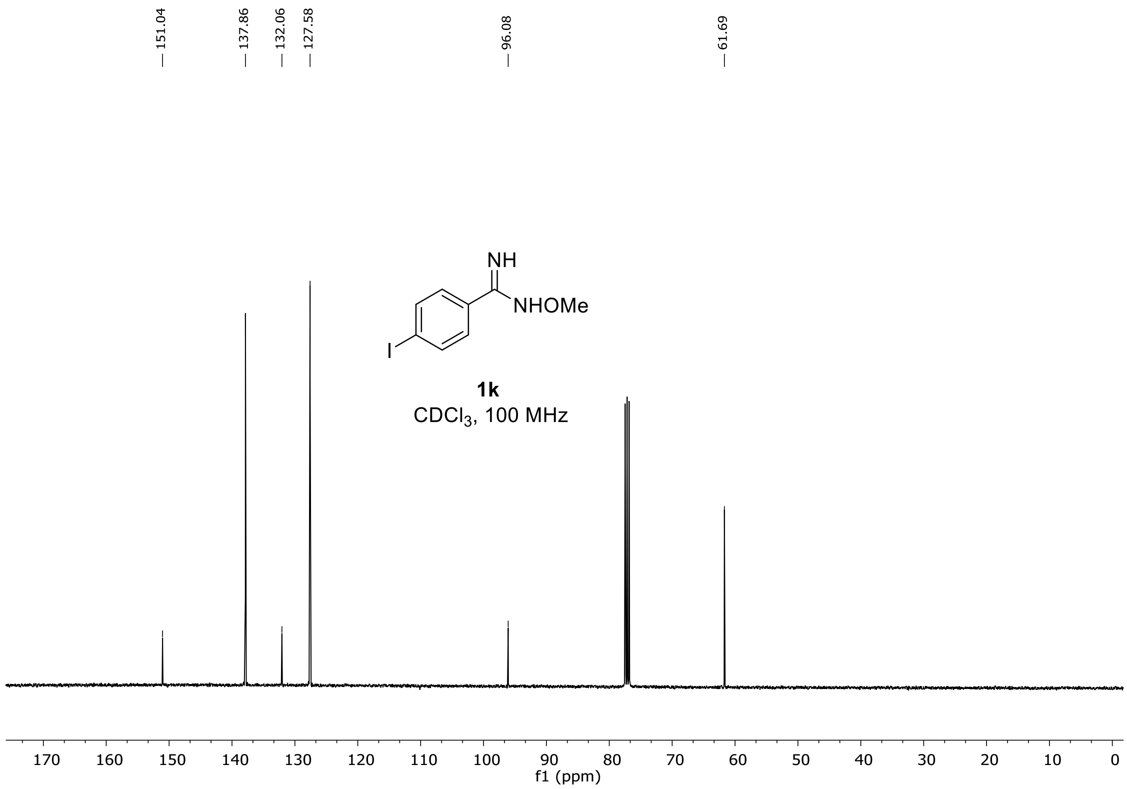

151.04
137.86
132.06
127.58
96.08
61.69
NH
NHOMe
I
1k
CDCl3, 100 MHz
170 160 150 140 130 120 110 100 90 80 70 60 50 40 30 20 10 0
f1 (ppm)

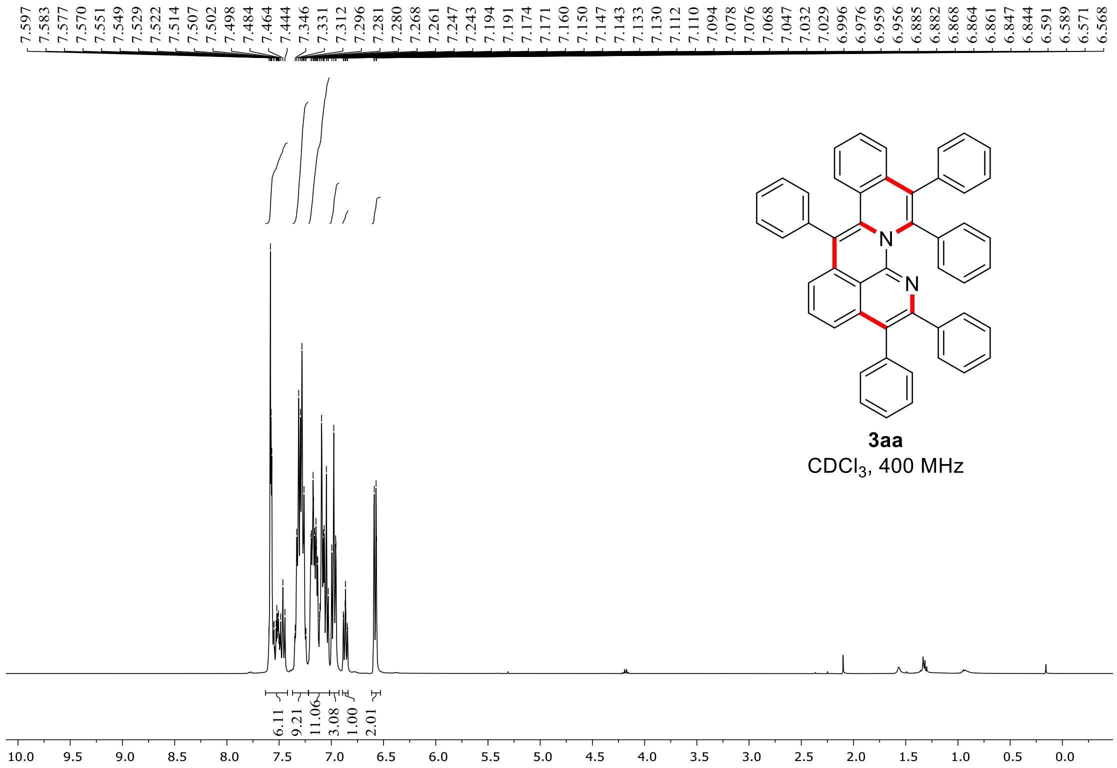

7.597 7.583 7.577 7.570 7.551 7.549 7.529 7.522 7.514 7.507 7.502 7.498 7.484 7.464 7.444 7.346 7.331 7.312 7.296 7.281 7.280 7.268 7.261 7.247 7.243 7.194 7.191 7.174 7.171 7.160 7.150 7.147 7.143 7.133 7.130 7.112 7.110 7.094 7.078 7.076 7.068 7.047 7.032 7.029 6.996 6.976 6.959 6.956 6.885 6.882 6.868 6.864 6.861 6.847 6.844 6.591 6.589 6.571 6.568
N
N
3aa
CDCl3, 400 MHz
6.11
9.21
11.06
3.08
1.00
2.01
10.0 9.5 9.0 8.5 8.0 7.5 7.0 6.5 6.0 5.5 5.0 4.5 4.0 3.5 3.0 2.5 2.0 1.5 1.0 0.5 0.0

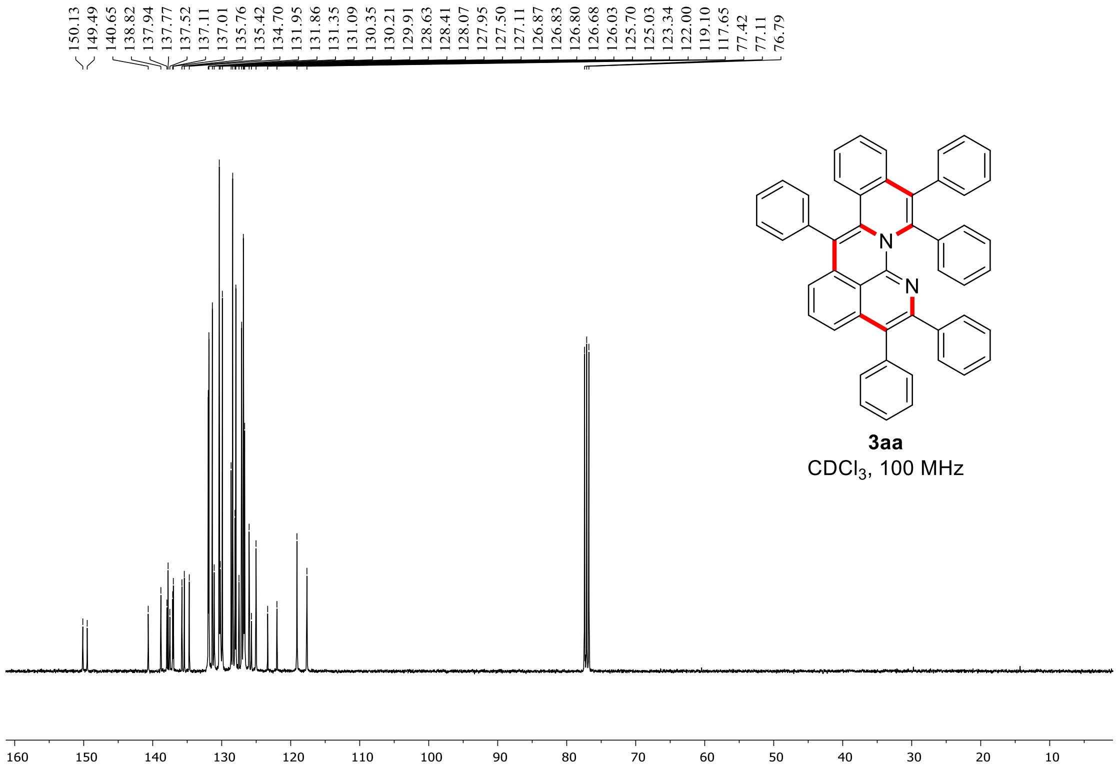
150.13
149.49
140.65
138.82
137.94
137.77
137.52
137.11
137.01
135.76
135.42
134.70
131.95
131.86
131.35
131.09
130.35
130.21
129.91
128.63
128.41
128.07
127.95
127.50
127.11
126.87
126.83
126.80
126.68
126.03
125.70
125.03
123.34
122.00
119.10
117.65
77.42
77.11
76.79
N
N
3aa
CDCl3, 100 MHz
160 150 140 130 120 110 100 90 80 70 60 50 40 30 20 10

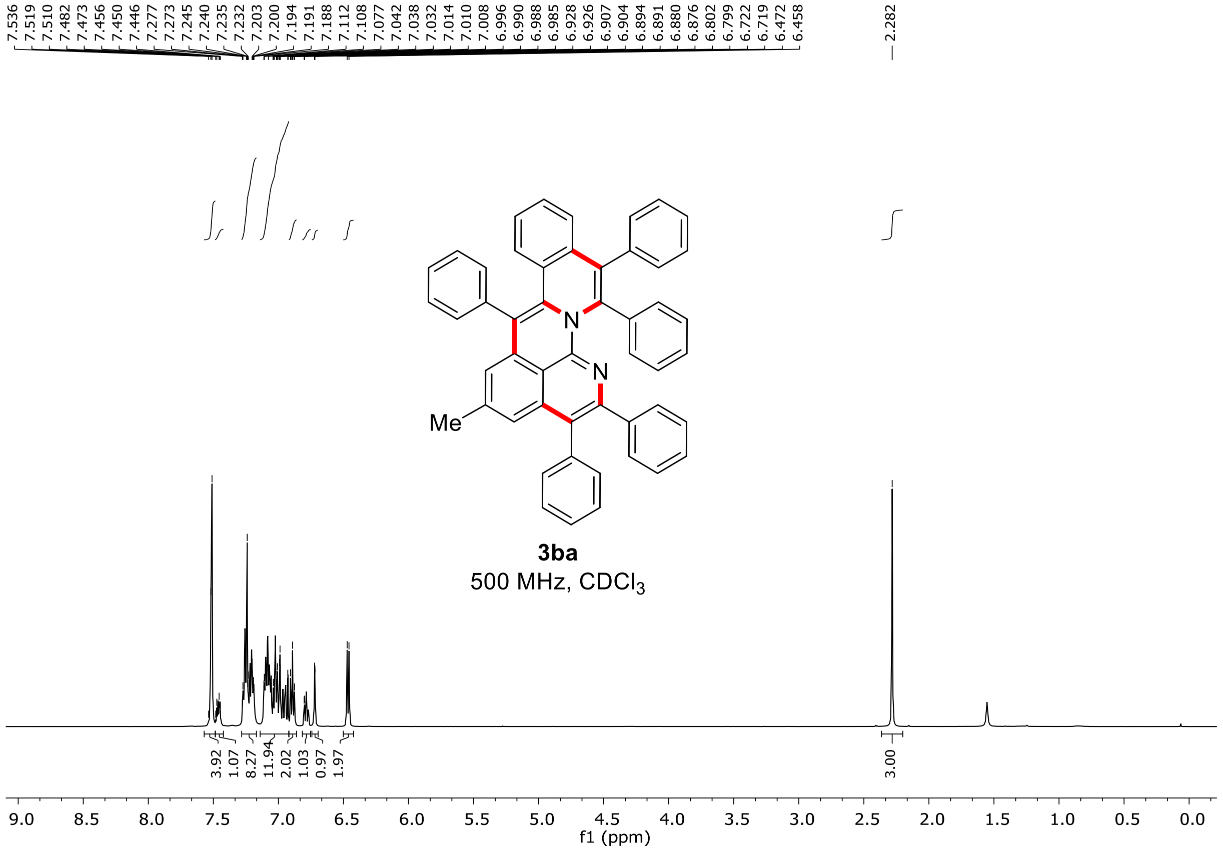
7.536
7.519
7.510
7.482
7.473
7.456
7.450
7.446
7.277
7.273
7.245
7.240
7.235
7.232
7.203
7.200
7.194
7.191
7.188
7.112
7.108
7.077
7.042
7.038
7.032
7.014
7.010
7.008
6.996
6.990
6.988
6.985
6.928
6.926
6.907
6.904
6.894
6.891
6.880
6.876
6.802
6.799
6.722
6.719
6.472
6.458
2.282
N
N
Me
3ba
500 MHz, CDCl3
3.92
1.07
8.27
11.94
2.02
1.03
0.97
1.97
3.00
9.0 8.5 8.0 7.5 7.0 6.5 6.0 5.5 5.0 4.5 4.0 3.5 3.0 2.5 2.0 1.5 1.0 0.5 0.0
f1 (ppm)

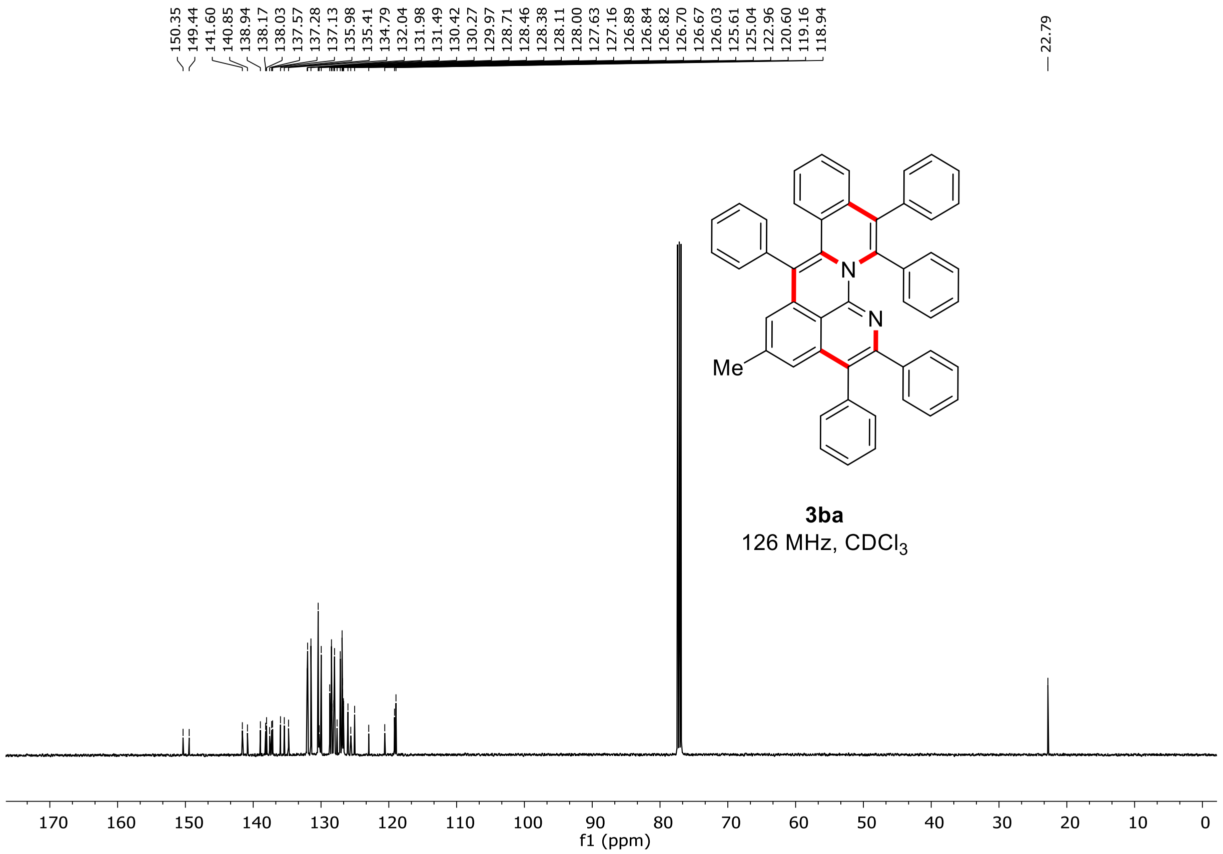
150.35
149.44
141.60
140.85
138.94
138.17
138.03
137.57
137.28
137.13
135.98
135.41
134.79
132.04
131.98
131.49
130.42
130.27
129.97
128.71
128.46
128.38
128.11
128.00
127.63
127.16
126.89
126.84
126.82
126.70
126.67
126.03
125.61
125.04
122.96
120.60
119.16
118.94
22.79
N
N
Me
3ba
126 MHz, CDCl3
170 160 150 140 130 120 110 100 90 80 70 60 50 40 30 20 10 0
f1 (ppm)

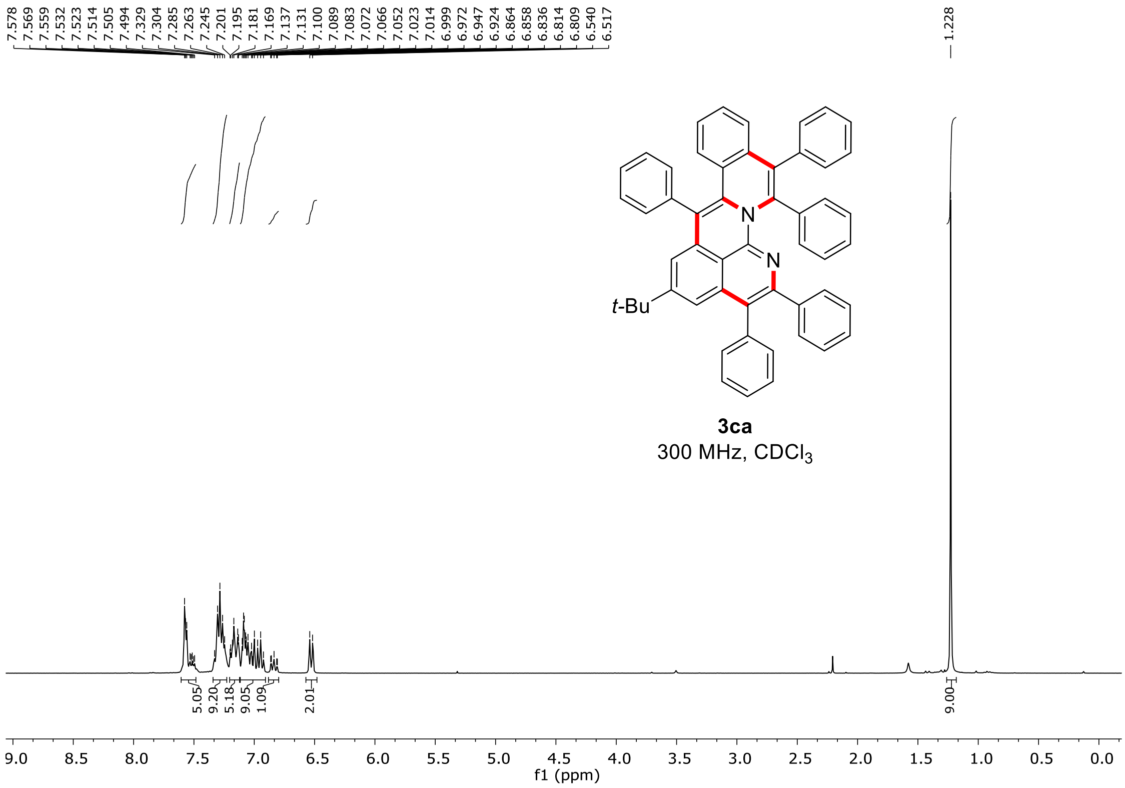
7.578
7.569
7.559
7.532
7.523
7.514
7.505
7.494
7.329
7.304
7.285
7.263
7.245
7.201
7.195
7.181
7.169
7.137
7.131
7.100
7.089
7.083
7.072
7.066
7.052
7.023
7.014
6.999
6.972
6.947
6.924
6.864
6.858
6.836
6.814
6.809
6.540
6.517
1.228
N
N
t-Bu
3ca
300 MHz, CDCl3
5.05
9.20
5.18
9.05
1.09
2.01
9.00
9.0 8.5 8.0 7.5 7.0 6.5 6.0 5.5 5.0 4.5 4.0 3.5 3.0 2.5 2.0 1.5 1.0 0.5 0.0
f1 (ppm)

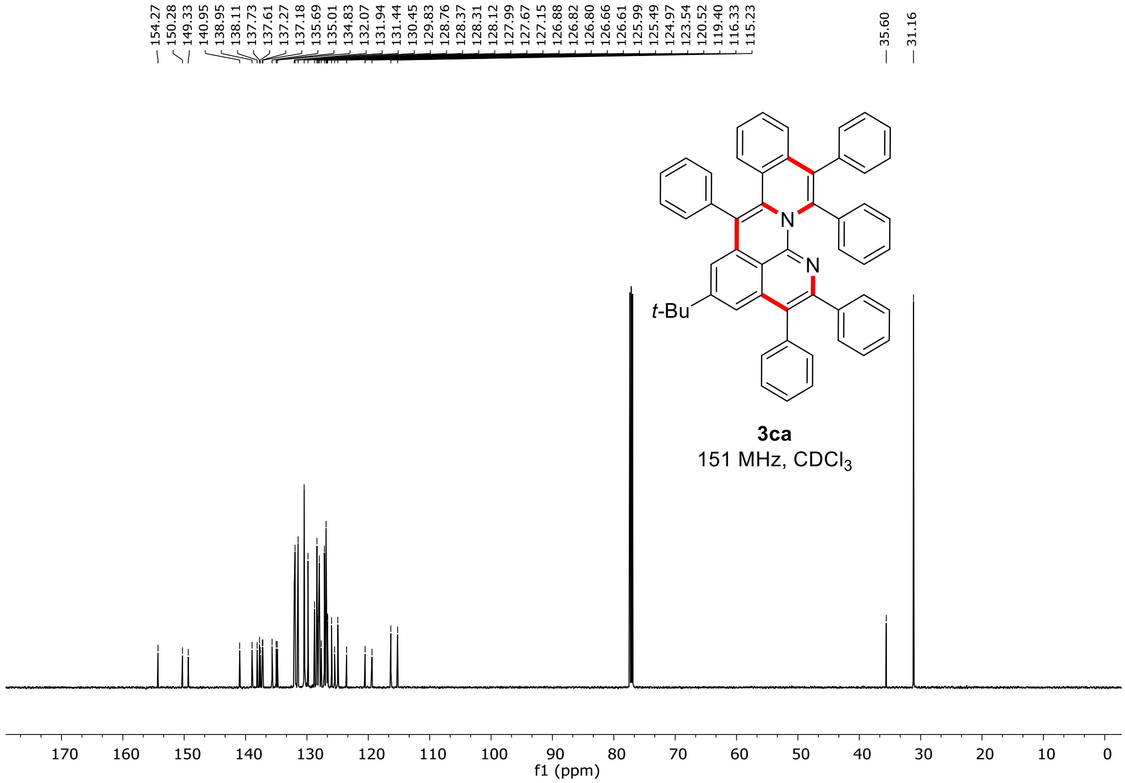
154.27
150.28
149.33
140.95
138.95
138.11
137.73
137.61
137.27
137.18
135.69
135.01
134.83
132.07
131.94
131.44
130.45
129.83
128.76
128.37
128.31
128.12
127.99
127.67
127.15
126.88
126.82
126.80
126.66
126.61
125.99
125.49
124.97
123.54
120.52
119.40
116.33
115.23
35.60
31.16
N
N
t-Bu
3ca
151 MHz, $CDCl_3$
170 160 150 140 130 120 110 100 90 80 70 60 50 40 30 20 10 0
f1 (ppm)

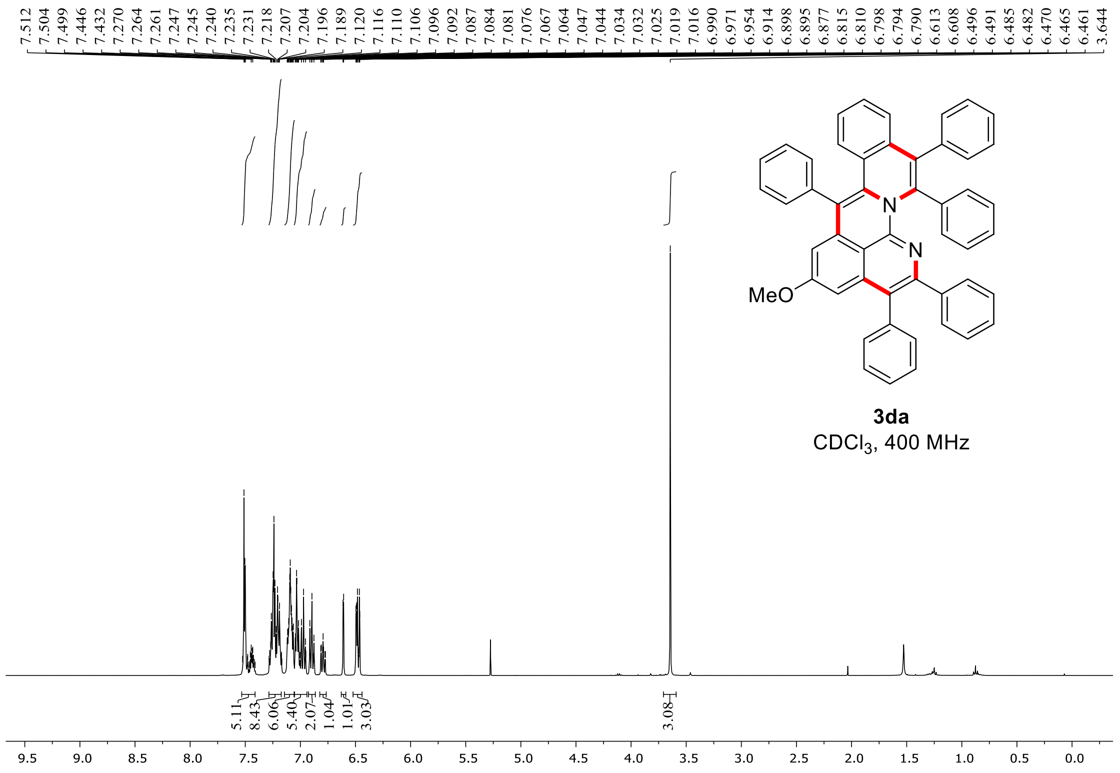
7.512
7.504
7.499
7.446
7.432
7.270
7.264
7.261
7.247
7.245
7.240
7.235
7.231
7.218
7.207
7.204
7.196
7.189
7.120
7.116
7.110
7.106
7.096
7.092
7.087
7.084
7.081
7.076
7.067
7.064
7.047
7.044
7.034
7.032
7.025
7.019
7.016
6.990
6.971
6.954
6.914
6.898
6.895
6.877
6.815
6.810
6.798
6.794
6.790
6.613
6.608
6.496
6.491
6.485
6.482
6.470
6.465
6.461
3.644
N
N
MeO
3da
$CDCl_3$, 400 MHz
5.11
8.43
6.06
5.40
2.07
1.04
1.01
3.03
3.08
9.5 9.0 8.5 8.0 7.5 7.0 6.5 6.0 5.5 5.0 4.5 4.0 3.5 3.0 2.5 2.0 1.5 1.0 0.5 0.0

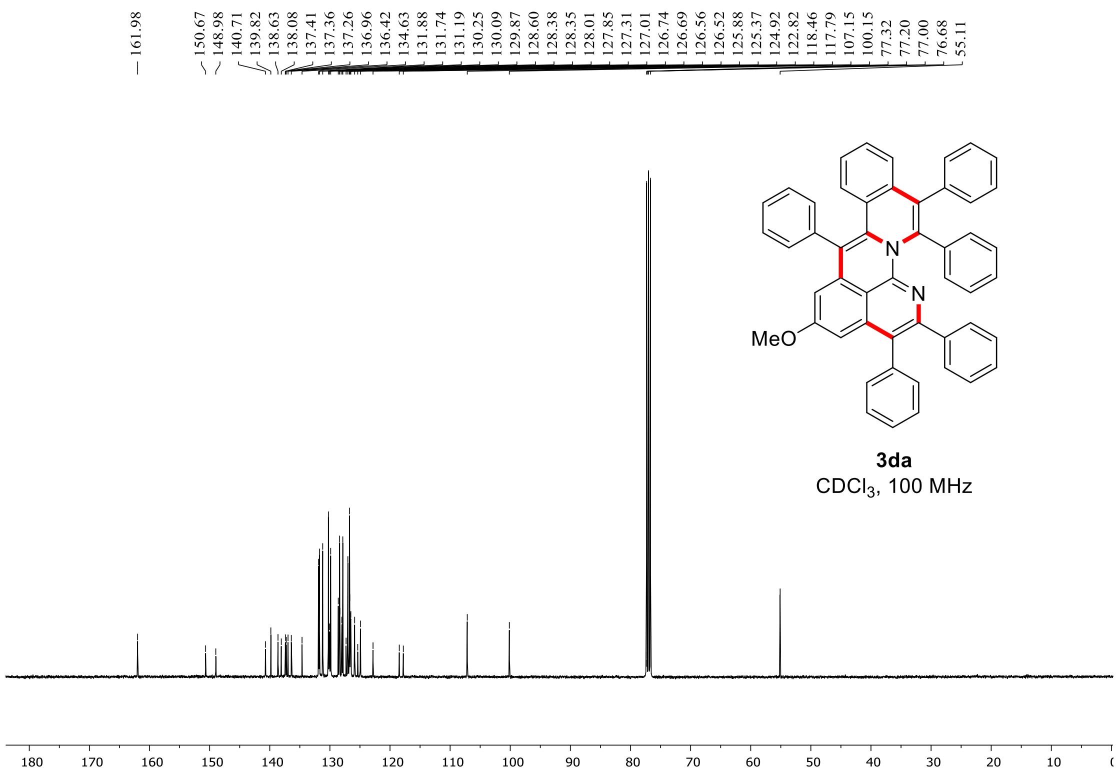
161.98
150.67
148.98
140.71
139.82
138.63
138.08
137.41
137.36
137.26
136.96
136.42
134.63
131.88
131.74
131.19
130.25
130.09
129.87
128.60
128.38
128.35
128.01
127.85
127.31
127.01
126.74
126.69
126.56
126.52
125.88
125.37
124.92
122.82
118.46
117.79
107.15
100.15
77.32
77.20
77.00
76.68
55.11
MeO
N
N
3da
CDCl3, 100 MHz
180 170 160 150 140 130 120 110 100 90 80 70 60 50 40 30 20 10

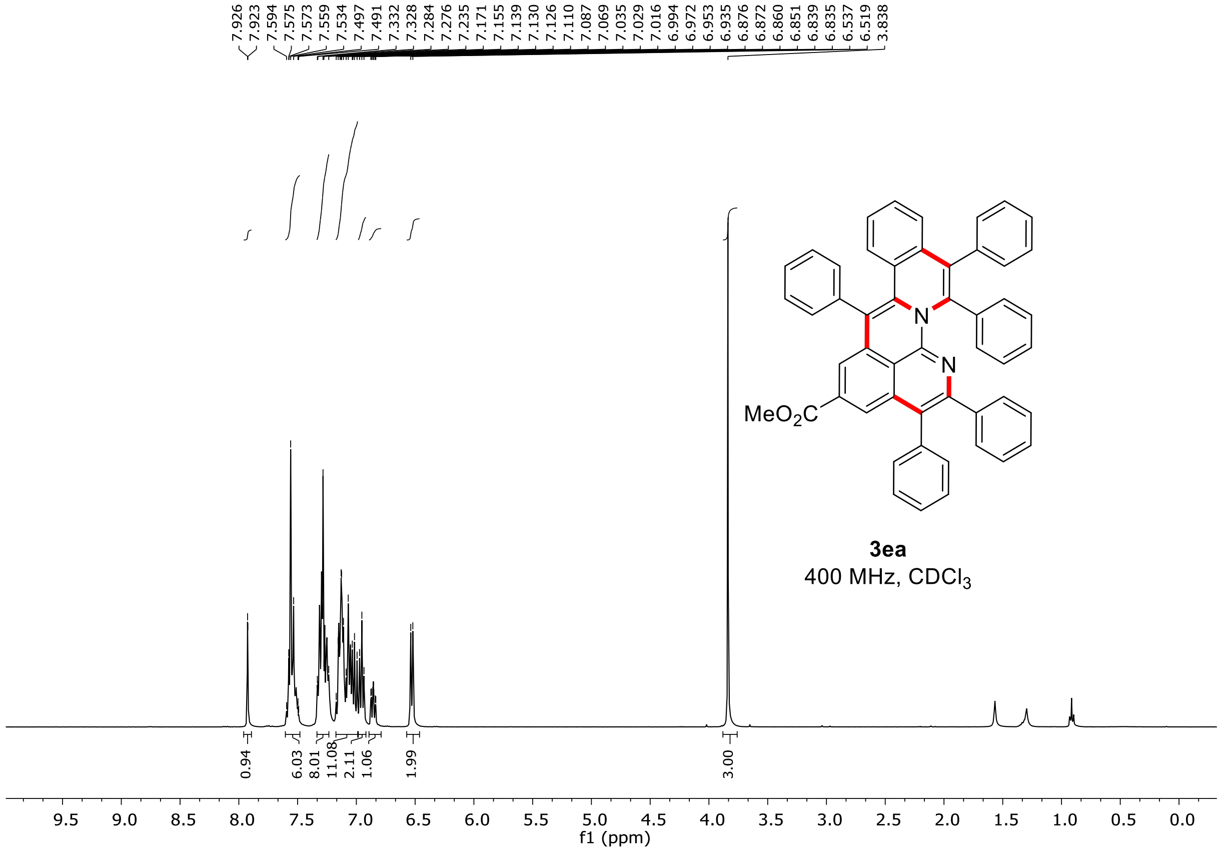
7.926
7.923
7.594
7.575
7.573
7.559
7.534
7.497
7.491
7.332
7.328
7.284
7.276
7.235
7.171
7.155
7.139
7.130
7.126
7.110
7.087
7.069
7.035
7.029
7.016
6.994
6.972
6.953
6.935
6.876
6.872
6.860
6.851
6.839
6.835
6.537
6.519
3.838
MeO2C
N
N
3ea
400 MHz, CDCl3
0.94
6.03
8.01
11.08
2.11
1.06
1.99
3.00
9.5 9.0 8.5 8.0 7.5 7.0 6.5 6.0 5.5 5.0 4.5 4.0 3.5 3.0 2.5 2.0 1.5 1.0 0.5 0.0
f1 (ppm)

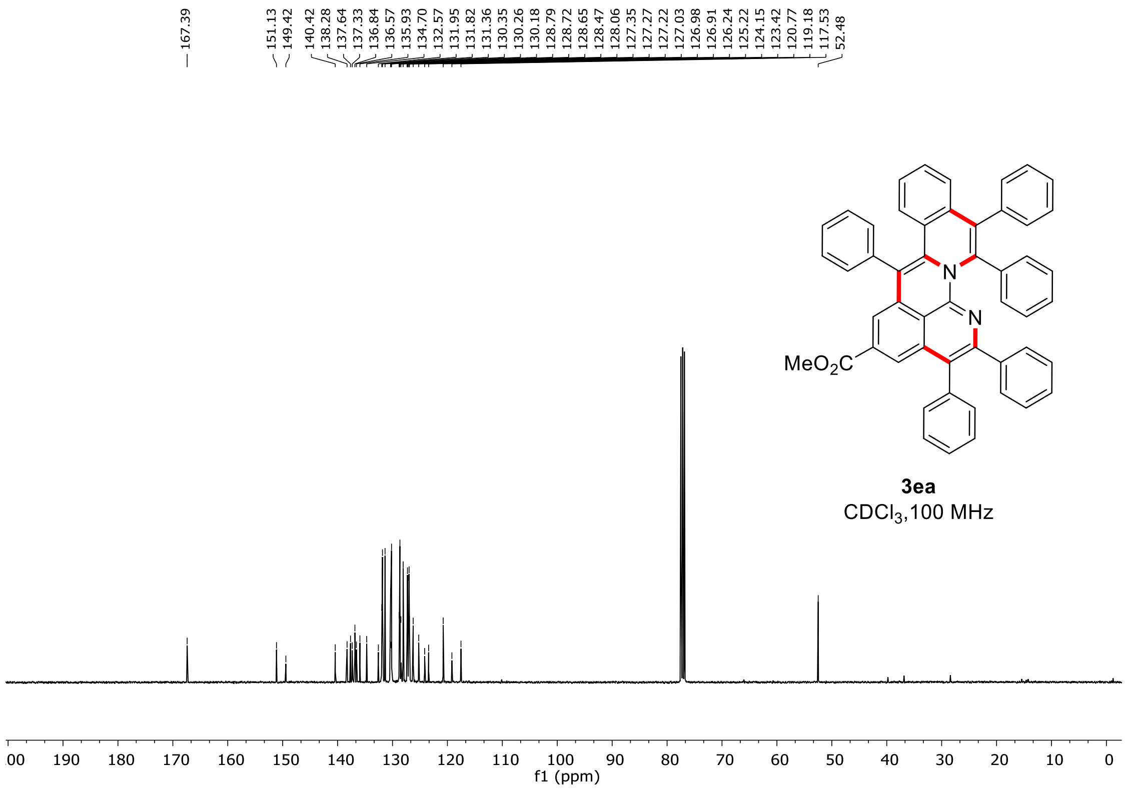
167.39
151.13
149.42
140.42
138.28
137.64
137.33
136.84
136.57
135.93
134.70
132.57
131.95
131.82
131.36
130.35
130.26
130.18
128.79
128.72
128.65
128.47
128.06
127.35
127.27
127.22
127.03
126.98
126.91
126.24
125.22
124.15
123.42
120.77
119.18
117.53
52.48
N
N
MeO2C
3ea
CDCl3,100 MHz
f1 (ppm)

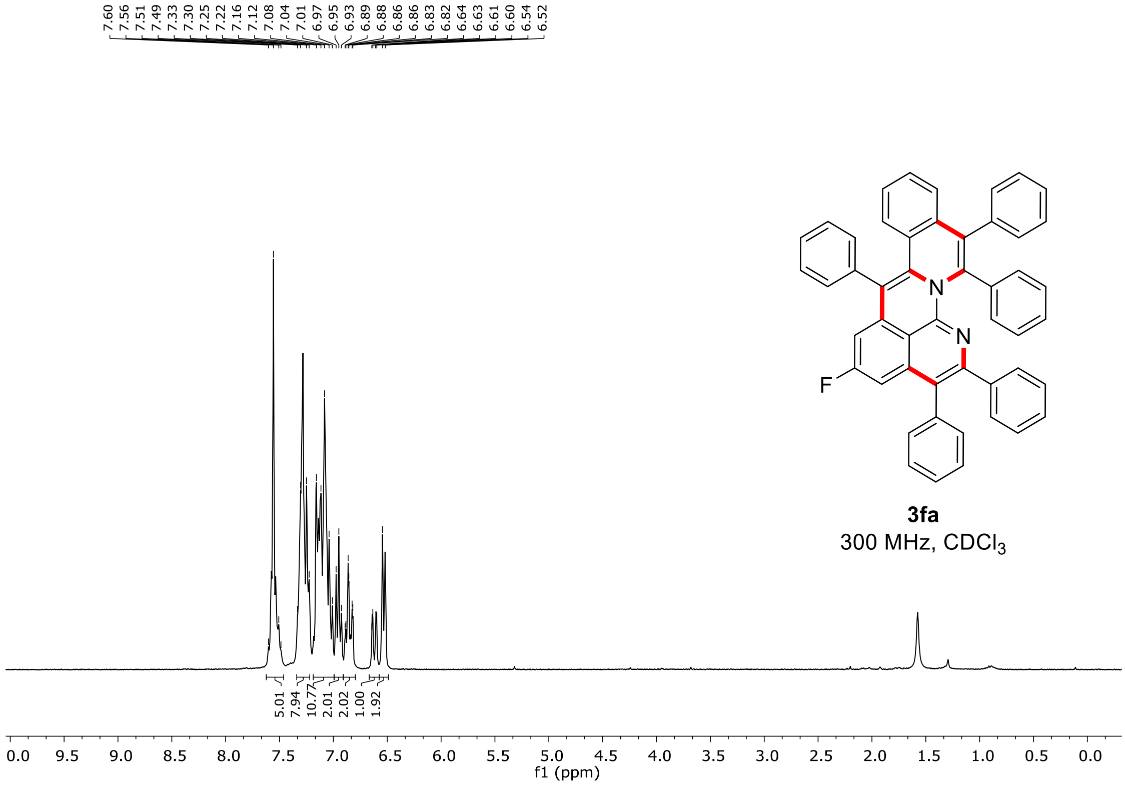
7.60
7.56
7.51
7.49
7.33
7.30
7.25
7.22
7.16
7.12
7.08
7.04
7.01
6.97
6.95
6.93
6.89
6.88
6.86
6.86
6.83
6.82
6.64
6.63
6.61
6.60
6.54
6.52
N
N
F
3fa
300 MHz, CDCl3
5.01
7.94
10.77
2.01
2.02
1.00
1.92
f1 (ppm)

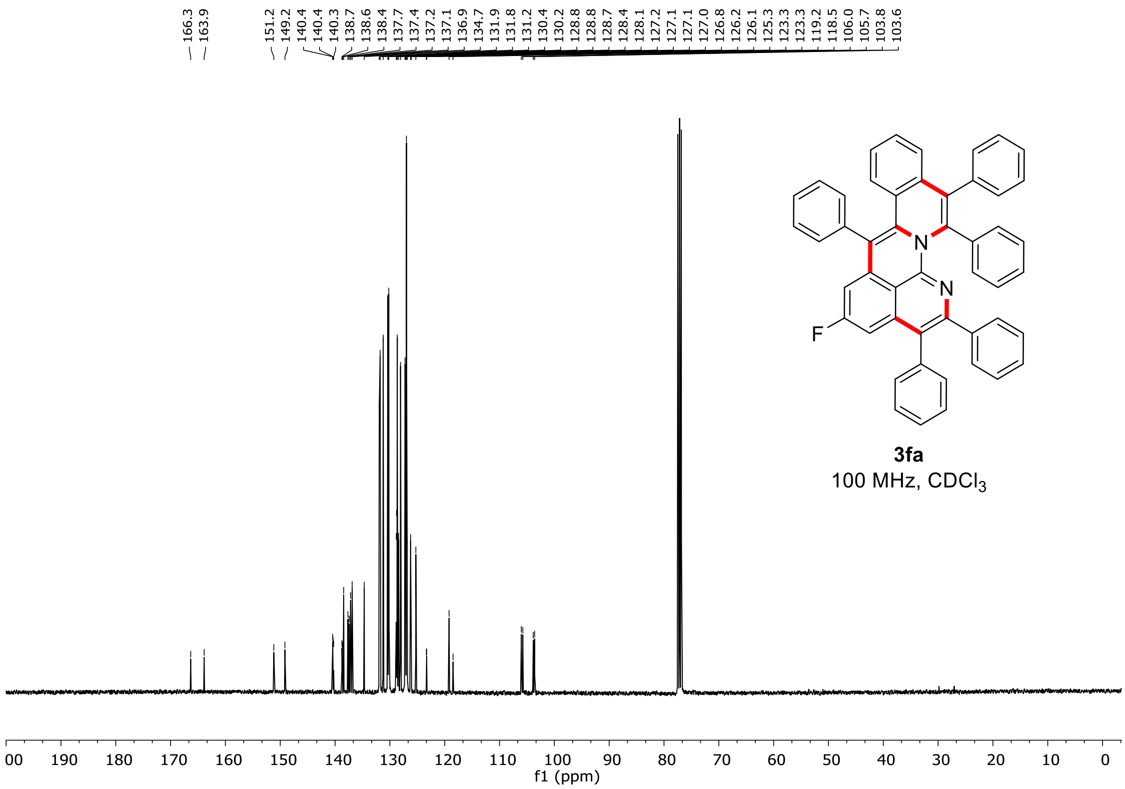

166.3
163.9
151.2
149.2
140.4
140.4
140.3
138.7
138.6
138.4
137.7
137.4
137.2
137.1
136.9
134.7
131.9
131.8
131.2
130.4
130.2
128.8
128.8
128.7
128.4
128.1
127.2
127.1
127.0
126.8
126.2
126.1
125.3
123.3
123.3
119.2
118.5
106.0
105.7
103.8
103.6
N
N
F
3fa
100 MHz, CDCl3
00 190 180 170 160 150 140 130 120 110 100 90 80 70 60 50 40 30 20 10 0
f1 (ppm)

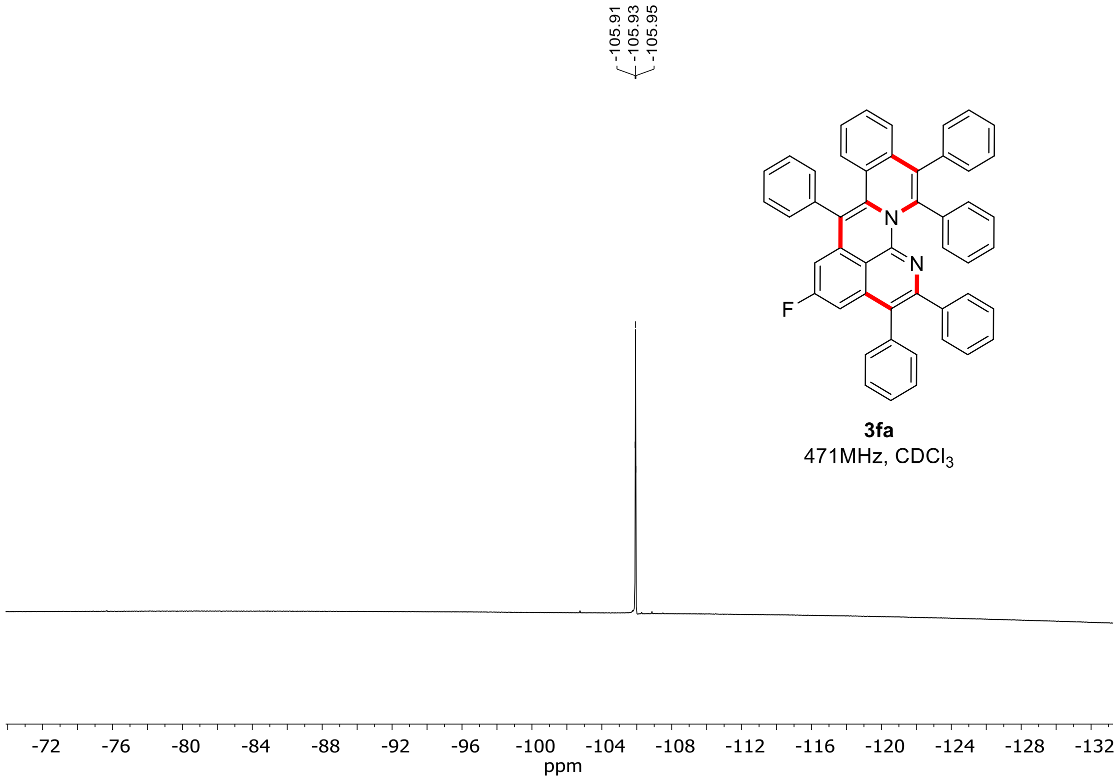

-105.91
-105.93
-105.95
N
N
F
3fa
471MHz, CDCl3
-72 -76 -80 -84 -88 -92 -96 -100 -104 -108 -112 -116 -120 -124 -128 -132
ppm

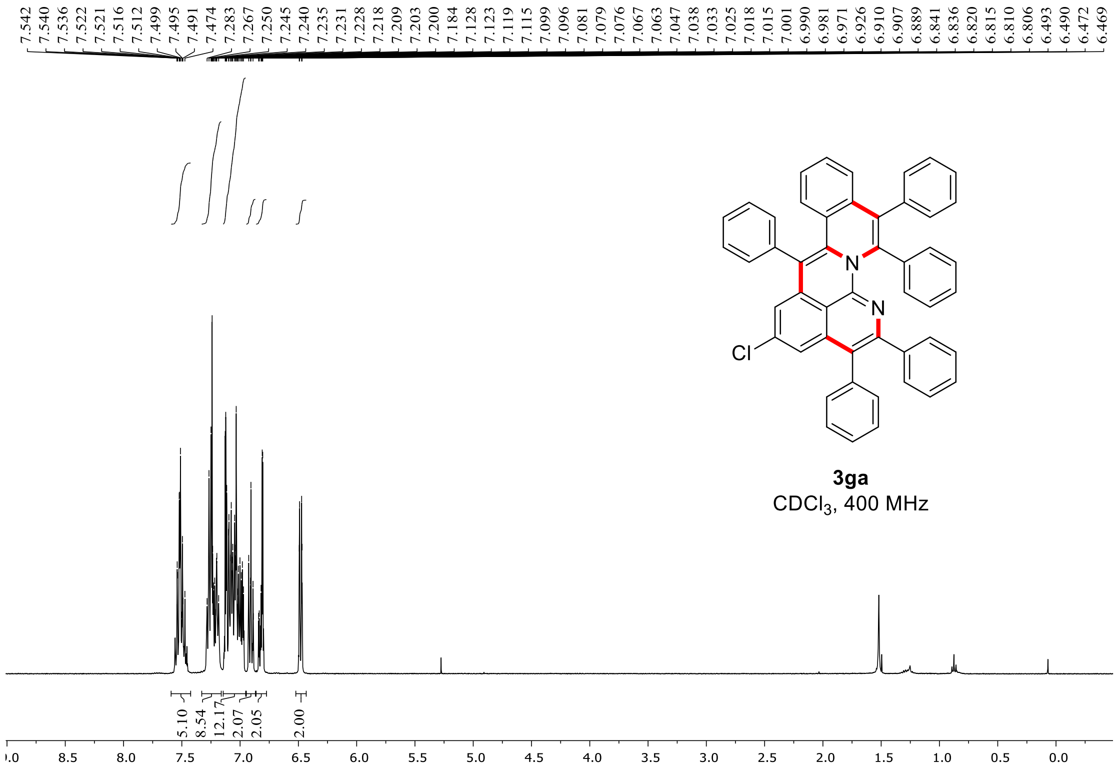
7.542
7.540
7.536
7.522
7.521
7.516
7.512
7.499
7.495
7.491
7.474
7.283
7.267
7.250
7.245
7.240
7.235
7.231
7.228
7.218
7.209
7.203
7.200
7.184
7.128
7.123
7.119
7.115
7.099
7.096
7.081
7.079
7.076
7.067
7.063
7.047
7.038
7.033
7.025
7.018
7.015
7.001
6.990
6.981
6.971
6.926
6.910
6.907
6.889
6.841
6.836
6.820
6.815
6.810
6.806
6.493
6.490
6.472
6.469
N
N
Cl
3ga
CDCl3, 400 MHz
5.10
8.54
12.17
2.07
2.05
2.00
8.5
8.0
7.5
7.0
6.5
6.0
5.5
5.0
4.5
4.0
3.5
3.0
2.5
2.0
1.5
1.0
0.5
0.0

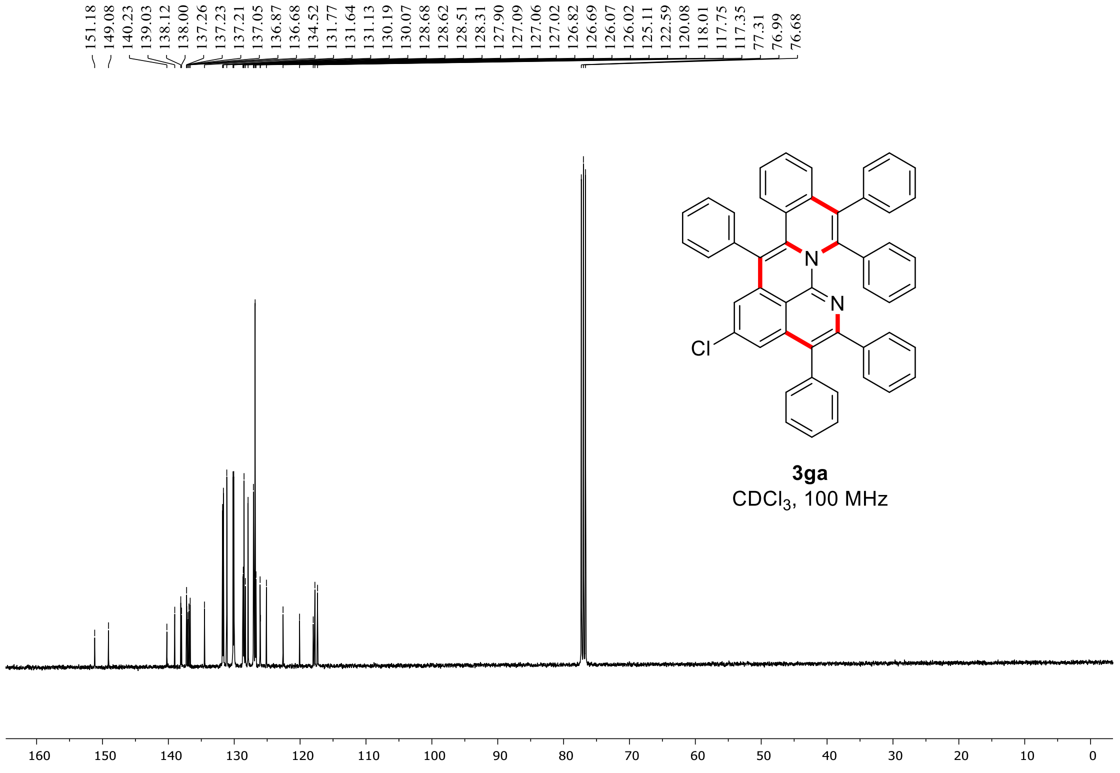
151.18
149.08
140.23
139.03
138.12
138.00
137.26
137.23
137.21
137.05
136.87
136.68
134.52
131.77
131.64
131.13
130.19
130.07
128.68
128.62
128.51
128.31
127.90
127.09
127.06
127.02
126.82
126.69
126.07
126.02
125.11
122.59
120.08
118.01
117.75
117.35
77.31
76.99
76.68
N
N
Cl
3ga
CDCl3, 100 MHz
160
150
140
130
120
110
100
90
80
70
60
50
40
30
20
10
0

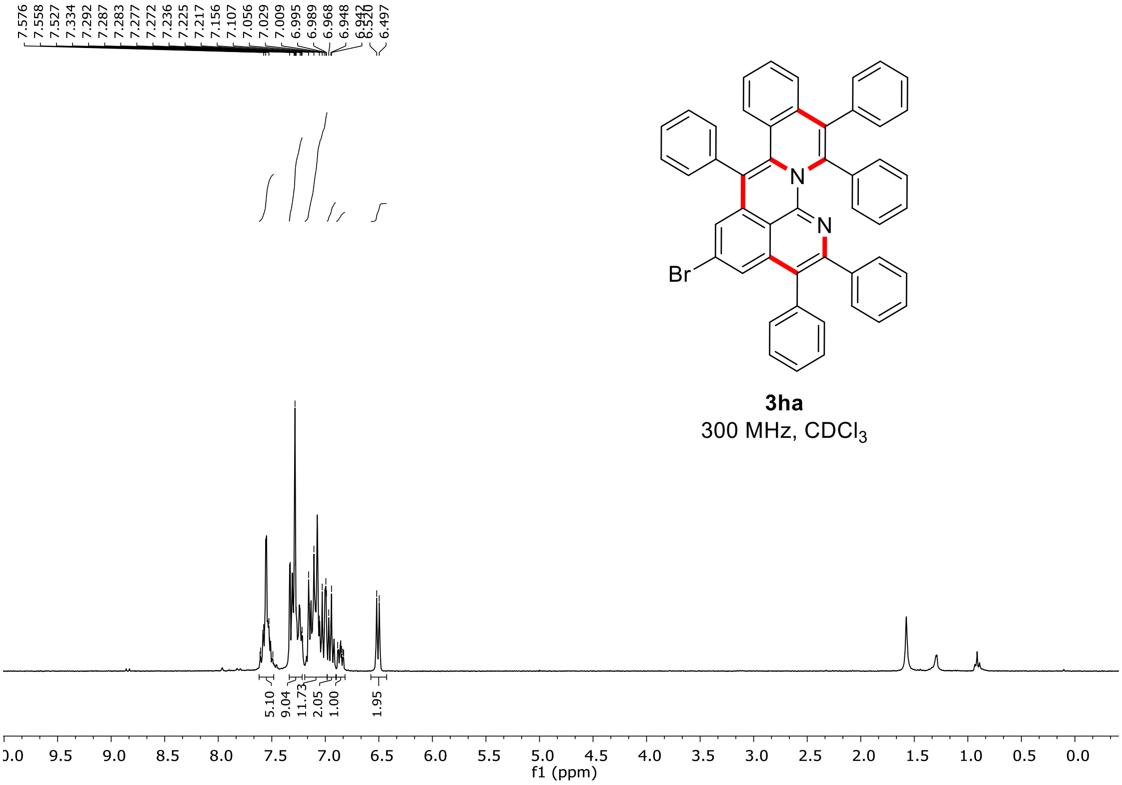
7.576
7.558
7.527
7.334
7.292
7.287
7.283
7.277
7.272
7.236
7.225
7.217
7.156
7.107
7.056
7.029
7.009
6.995
6.989
6.968
6.948
6.942
6.520
6.497
Br
N
N
3ha
300 MHz, $CDCl_3$
5.10
9.04
11.73
2.05
1.00
1.95
f1 (ppm)

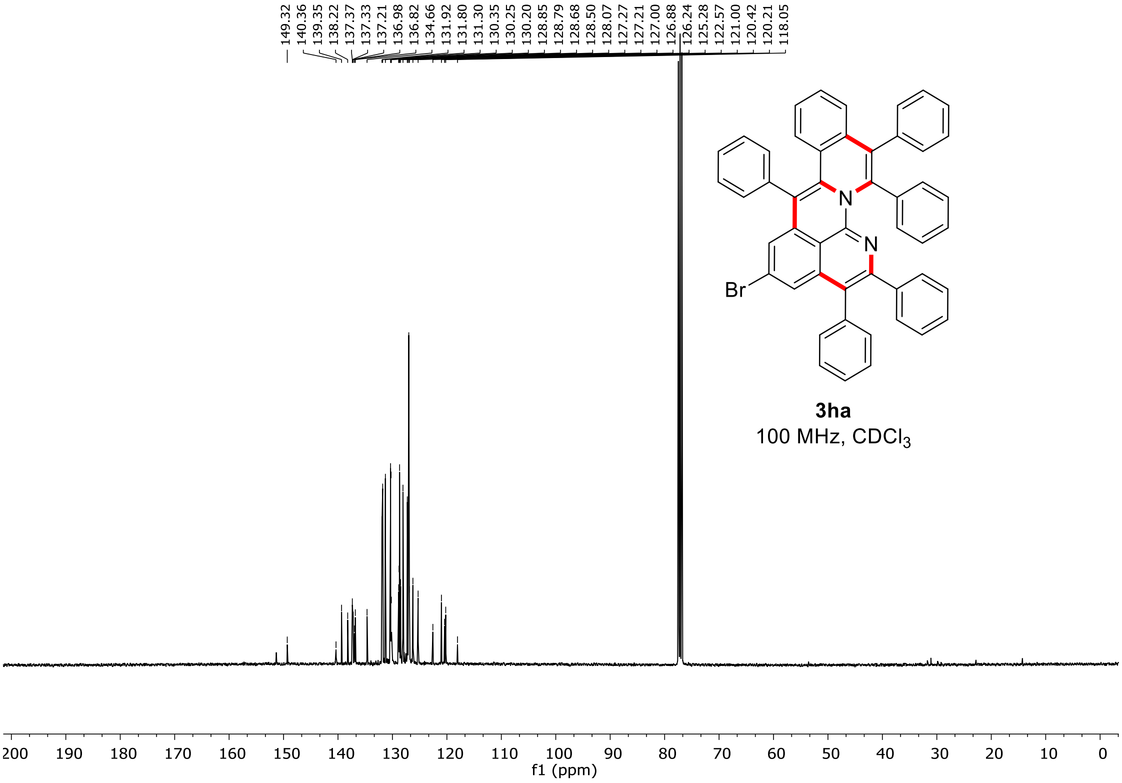
149.32
140.36
139.35
138.22
137.37
137.33
137.21
136.98
136.82
134.66
131.92
131.80
131.30
130.35
130.25
130.20
128.85
128.79
128.68
128.50
128.07
127.27
127.21
127.00
126.88
126.24
125.28
122.57
121.00
120.42
120.21
118.05
Br
N
N
3ha
100 MHz, $CDCl_3$
f1 (ppm)

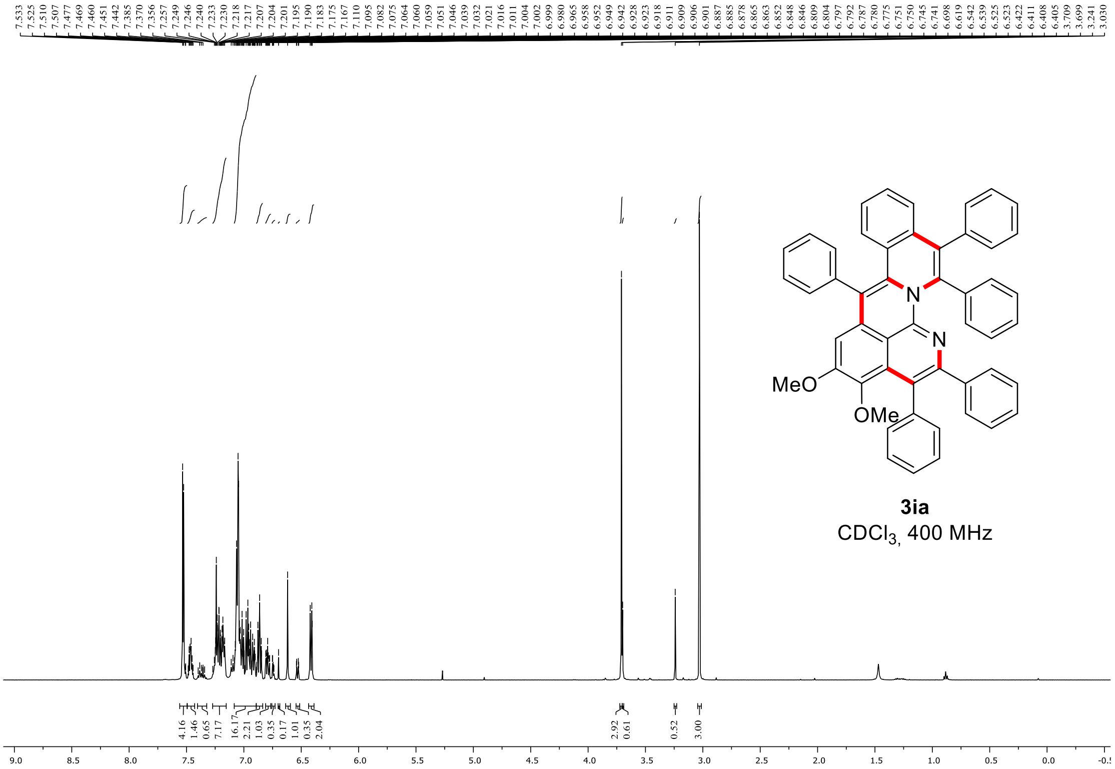
7.533 7.525 7.510 7.507 7.477 7.469 7.460 7.451 7.442 7.385 7.370 7.356 7.257 7.249 7.246 7.240 7.233 7.230 7.218 7.217 7.207 7.204 7.201 7.195 7.190 7.183 7.175 7.167 7.110 7.095 7.082 7.075 7.064 7.060 7.059 7.051 7.046 7.039 7.032 7.021 7.016 7.011 7.004 7.002 6.999 6.980 6.965 6.958 6.952 6.949 6.942 6.928 6.923 6.918 6.911 6.909 6.906 6.901 6.887 6.885 6.878 6.865 6.863 6.852 6.848 6.846 6.809 6.804 6.797 6.792 6.787 6.780 6.775 6.751 6.750 6.745 6.741 6.698 6.619 6.542 6.539 6.525 6.523 6.422 6.411 6.408 6.405 3.709 3.699 3.241 3.030
MeO
OMe
N
3ia
CDCl3, 400 MHz
4.16 1.46 0.65 7.17 16.17 2.21 1.03 0.35 0.17 1.01 0.35 2.04 2.92 0.61 0.52 3.00
9.0 8.5 8.0 7.5 7.0 6.5 6.0 5.5 5.0 4.5 4.0 3.5 3.0 2.5 2.0 1.5 1.0 0.5 0.0

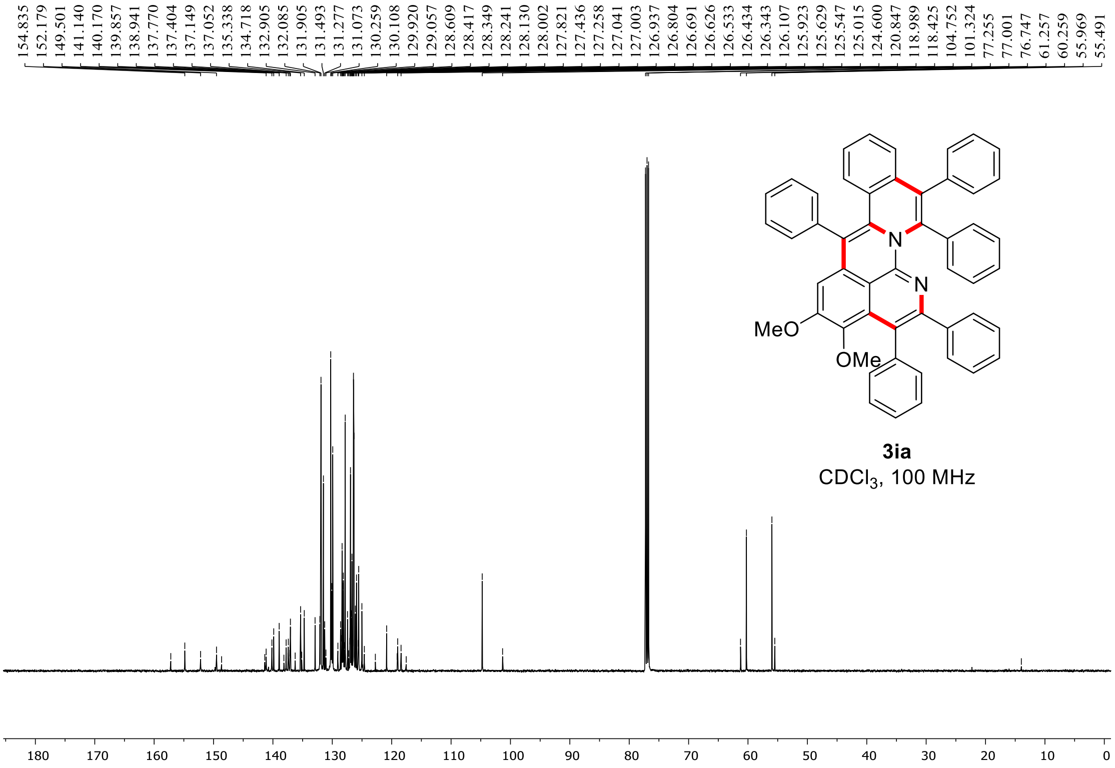
154.835 152.179 149.501 141.140 140.170 139.857 138.941 137.770 137.404 137.149 137.052 135.338 134.718 132.905 132.085 131.905 131.493 131.277 131.073 130.259 130.108 129.920 129.057 128.609 128.417 128.349 128.241 128.130 128.002 127.821 127.436 127.258 127.041 127.003 126.937 126.804 126.691 126.626 126.533 126.434 126.343 126.107 125.923 125.629 125.547 125.015 124.600 120.847 118.989 118.425 104.752 101.324 77.255 77.001 76.747 61.257 60.259 55.969 55.491
MeO
OMe
N
3ia
CDCl3, 100 MHz
180 170 160 150 140 130 120 110 100 90 80 70 60 50 40 30 20 10 0

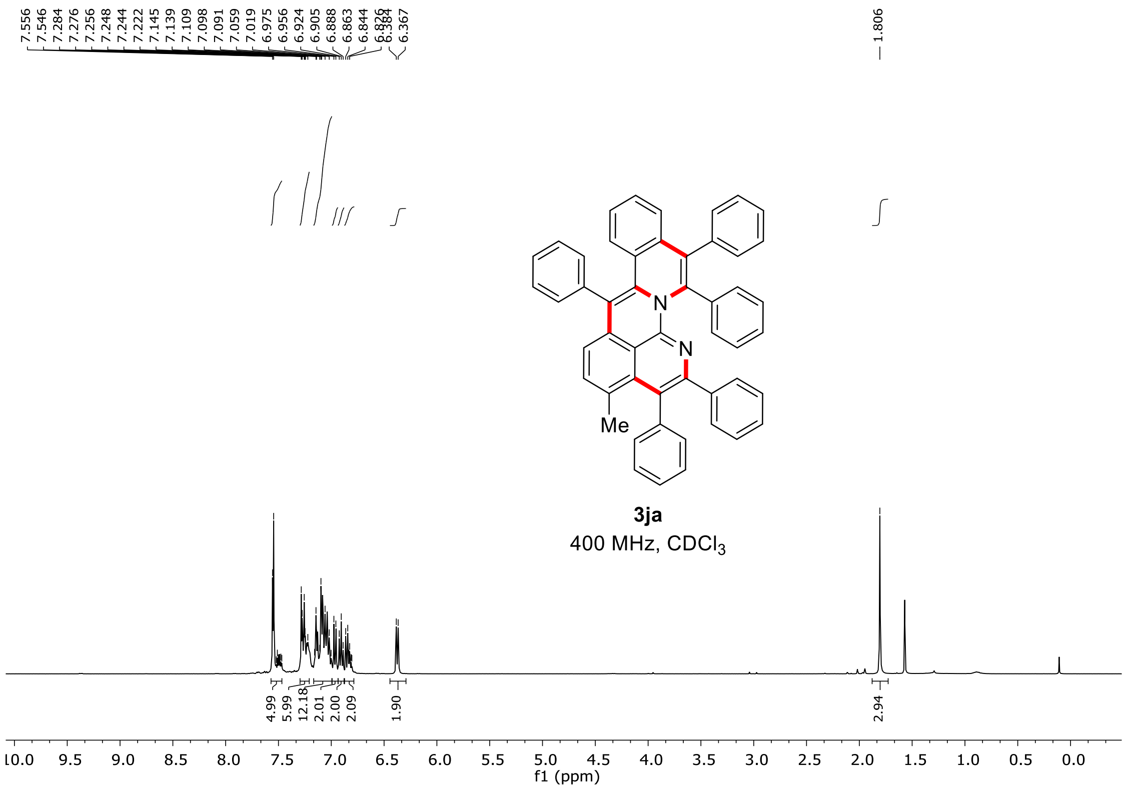
7.556
7.546
7.284
7.276
7.256
7.248
7.244
7.222
7.145
7.139
7.109
7.098
7.091
7.059
7.019
6.975
6.956
6.924
6.905
6.888
6.863
6.844
6.826
6.384
6.367
1.806
Me
N
N
3ja
400 MHz, CDCl3
4.99
5.99
12.18
2.01
2.00
2.09
1.90
2.94
f1 (ppm)

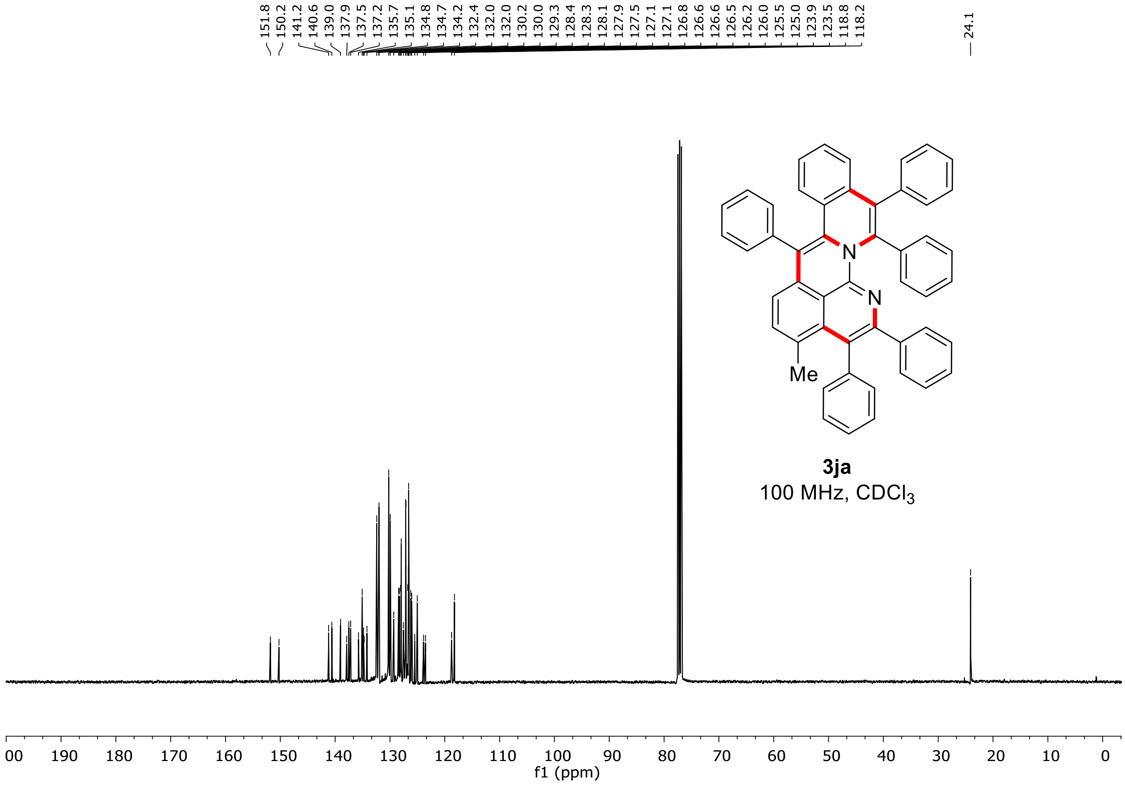
151.8
150.2
141.2
140.6
139.0
137.9
137.5
137.2
135.7
135.1
134.8
134.7
134.2
132.4
132.0
132.0
130.2
130.0
129.3
128.4
128.3
128.1
127.9
127.5
127.1
127.1
126.8
126.6
126.6
126.5
126.2
126.0
125.5
125.0
123.9
123.5
118.8
118.2
24.1
Me
N
N
3ja
100 MHz, CDCl3
f1 (ppm)

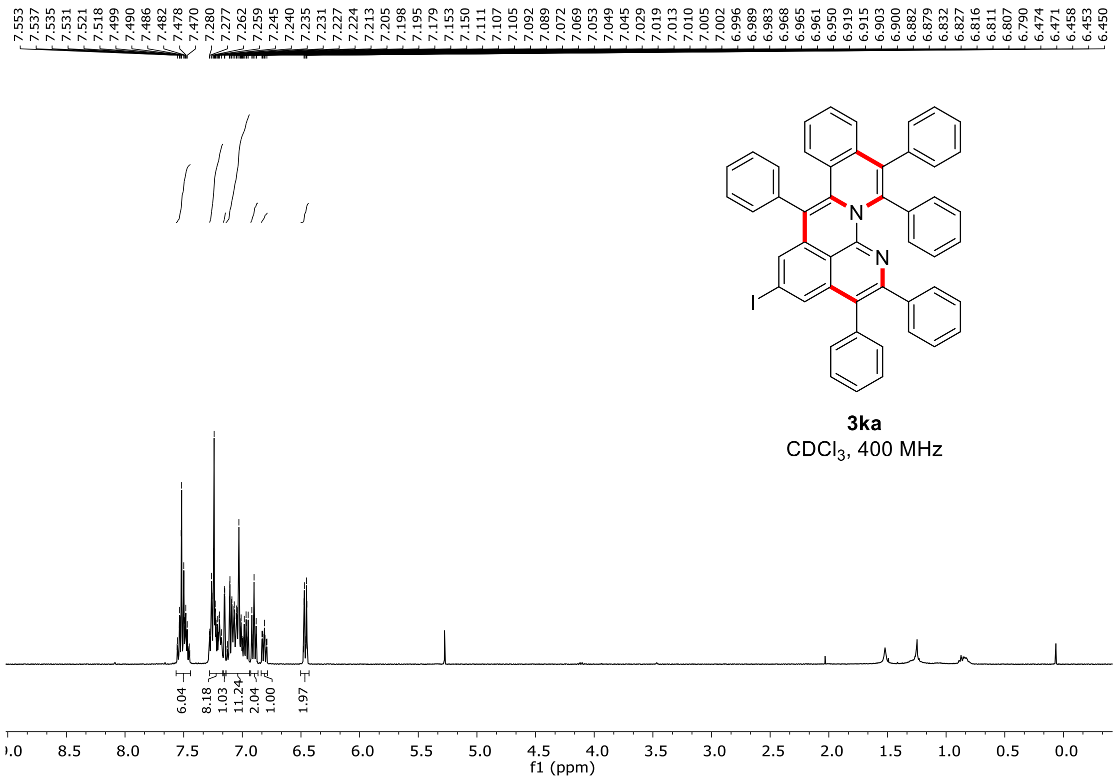
7.553 7.537 7.535 7.531 7.521 7.518 7.499 7.490 7.486 7.482 7.478 7.470 7.280 7.277 7.262 7.259 7.245 7.240 7.235 7.231 7.227 7.224 7.213 7.205 7.198 7.195 7.179 7.153 7.150 7.111 7.107 7.105 7.092 7.089 7.072 7.069 7.053 7.049 7.045 7.029 7.019 7.013 7.010 7.005 7.002 6.996 6.989 6.983 6.968 6.965 6.961 6.950 6.919 6.915 6.903 6.900 6.882 6.879 6.832 6.827 6.816 6.811 6.807 6.790 6.474 6.471 6.458 6.453 6.450
N
N
I
3ka
$CDCl_3$, 400 MHz
6.04
8.18
1.03
11.24
2.04
1.00
1.97
f1 (ppm)

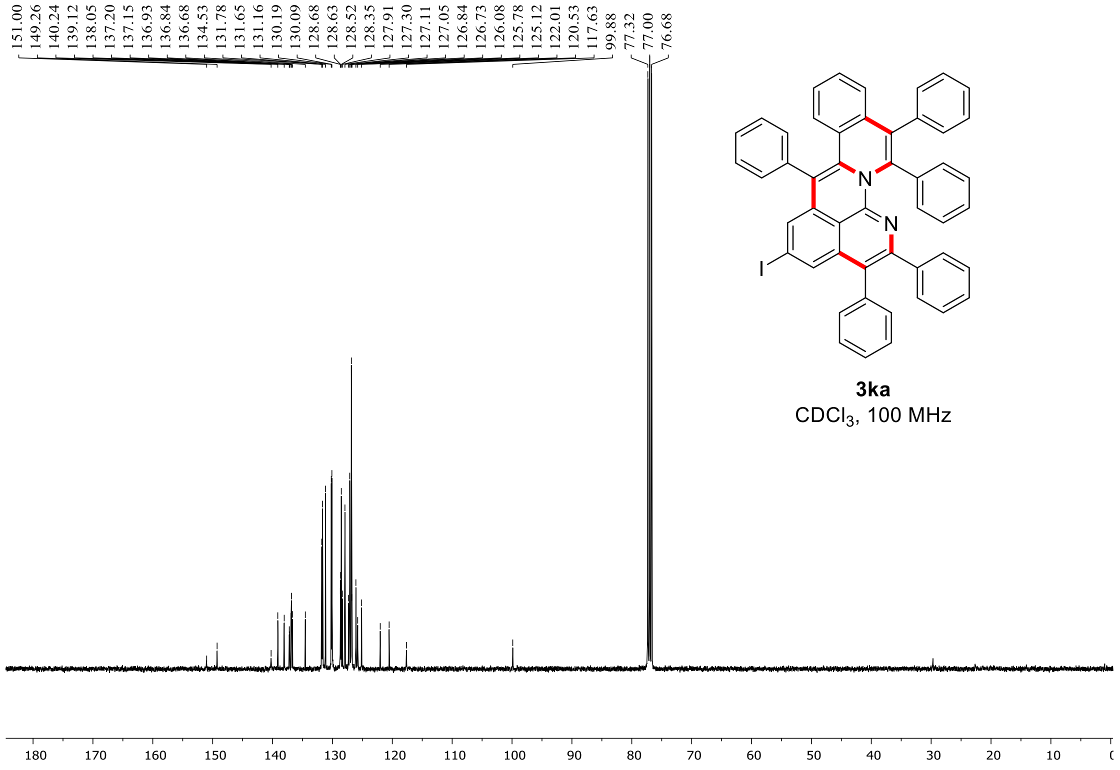
151.00 149.26 140.24 139.12 138.05 137.20 137.15 136.93 136.84 136.68 134.53 131.78 131.65 131.16 130.19 130.09 128.68 128.63 128.52 128.35 127.91 127.30 127.11 127.05 126.84 126.73 126.08 125.78 125.12 122.01 120.53 117.63 99.88 77.32 77.00 76.68
N
N
I
3ka
$CDCl_3$, 100 MHz

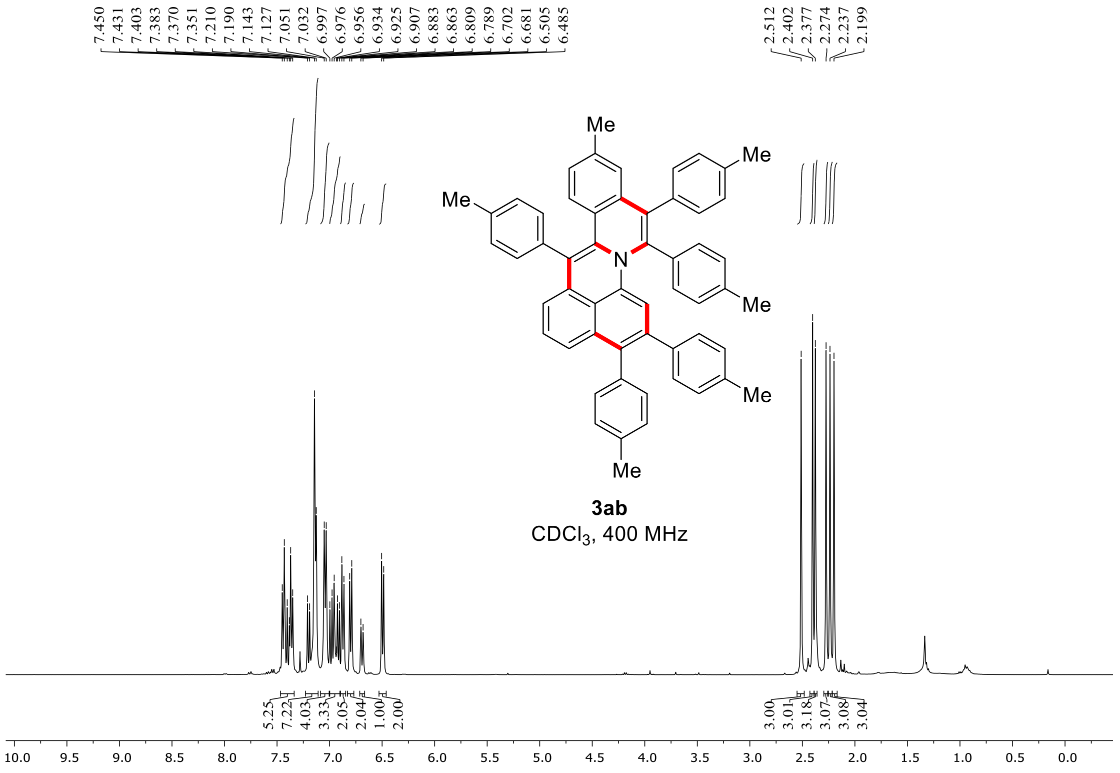
7.450 7.431 7.403 7.383 7.370 7.351 7.210 7.190 7.143 7.127 7.051 7.032 6.997 6.976 6.956 6.934 6.925 6.907 6.883 6.863 6.809 6.789 6.702 6.681 6.505 6.485
2.512 2.402 2.377 2.274 2.237 2.199
Me
Me
Me
N
Me
Me
Me
3ab
$CDCl_3$, 400 MHz
5.25 7.22 4.03 3.33 2.05 2.04 1.00 2.00
3.00 3.01 3.18 3.07 3.08 3.04
10.0 9.5 9.0 8.5 8.0 7.5 7.0 6.5 6.0 5.5 5.0 4.5 4.0 3.5 3.0 2.5 2.0 1.5 1.0 0.5 0.0

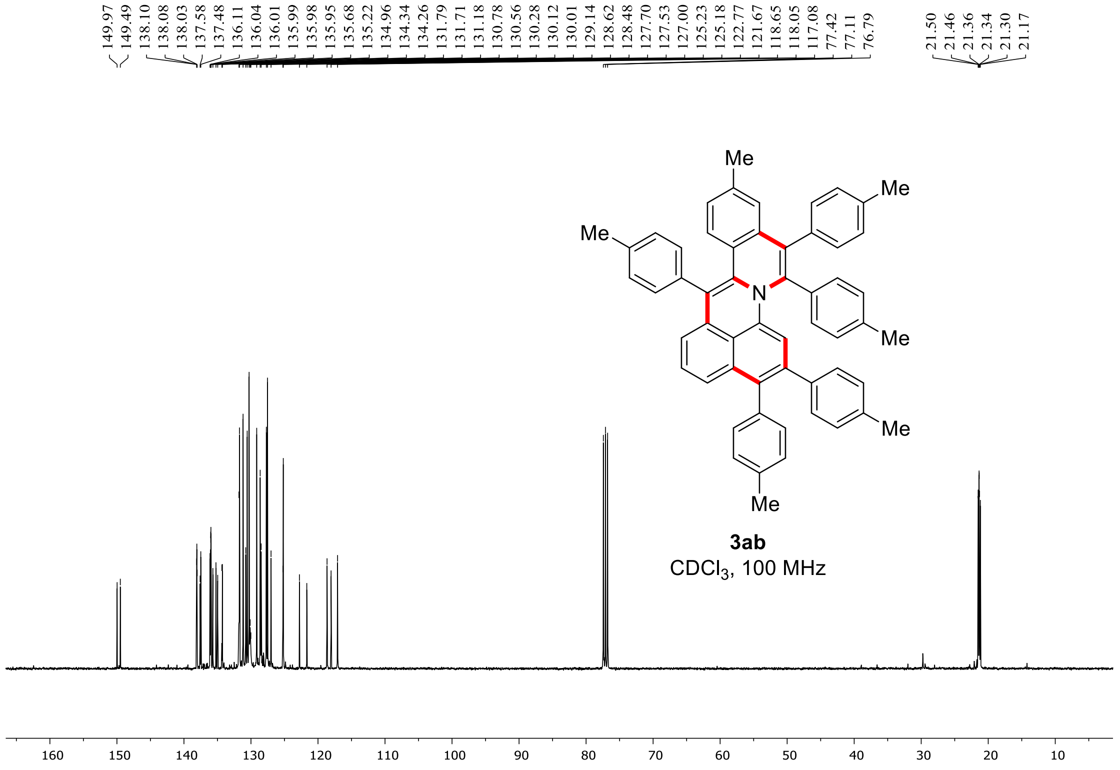
149.97 149.49 138.10 138.08 138.03 137.58 137.48 136.11 136.04 136.01 135.99 135.98 135.95 135.68 135.22 134.96 134.34 134.26 131.79 131.71 131.18 130.78 130.56 130.28 130.12 130.01 129.14 128.62 128.48 127.70 127.53 127.00 125.23 125.18 122.77 121.67 118.65 118.05 117.08 77.42 77.11 76.79
21.50 21.46 21.36 21.34 21.30 21.17
Me
Me
Me
N
Me
Me
Me
3ab
$CDCl_3$, 100 MHz
160 150 140 130 120 110 100 90 80 70 60 50 40 30 20 10

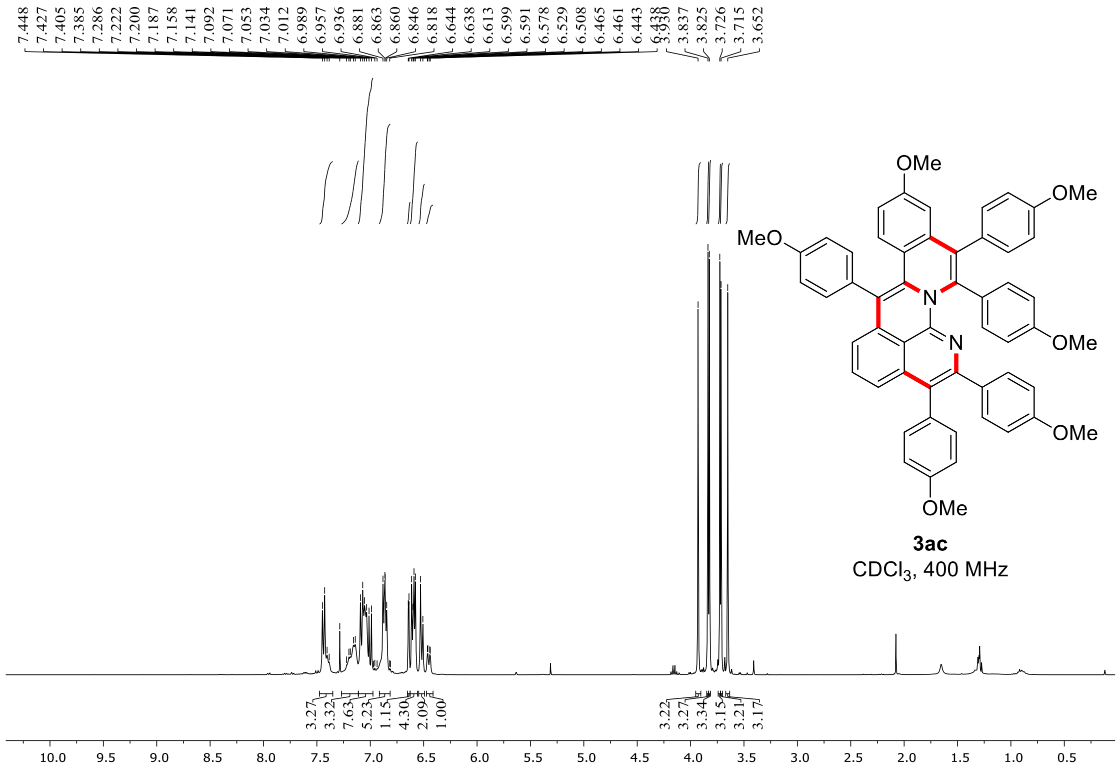
7.448
7.427
7.405
7.385
7.286
7.222
7.200
7.187
7.158
7.141
7.092
7.071
7.053
7.034
7.012
6.989
6.957
6.936
6.881
6.863
6.860
6.846
6.818
6.644
6.638
6.613
6.599
6.591
6.578
6.529
6.508
6.465
6.461
6.443
6.438
3.930
3.837
3.825
3.726
3.715
3.652
3.27
3.32
7.63
5.23
1.15
4.30
2.09
1.00
3.22
3.27
3.34
3.15
3.21
3.17
10.0 9.5 9.0 8.5 8.0 7.5 7.0 6.5 6.0 5.5 5.0 4.5 4.0 3.5 3.0 2.5 2.0 1.5 1.0 0.5
OMe
OMe
MeO
N
OMe
N
OMe
OMe
3ac
CDCl3, 400 MHz

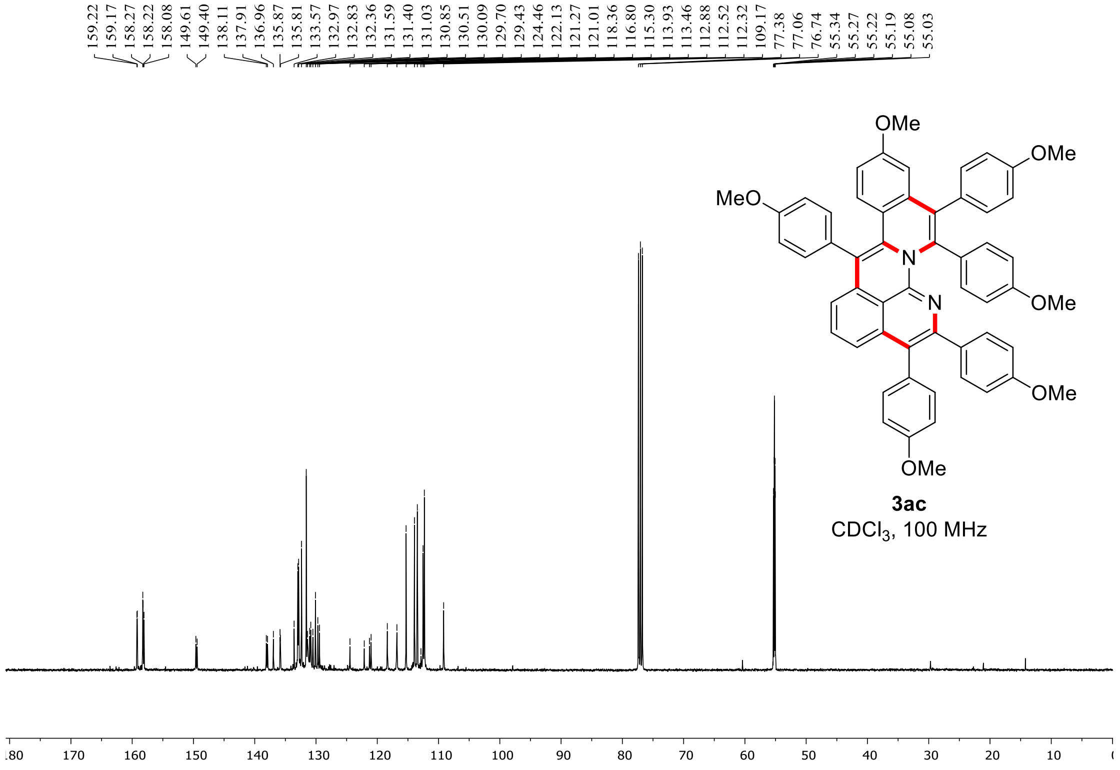
159.22
159.17
158.27
158.22
158.08
149.61
149.40
138.11
137.91
136.96
135.87
135.81
133.57
132.97
132.83
132.36
131.59
131.40
131.03
130.85
130.51
130.09
129.70
129.43
124.46
122.13
121.27
121.01
118.36
116.80
115.30
113.93
113.46
112.88
112.52
112.32
109.17
77.38
77.06
76.74
55.34
55.27
55.22
55.19
55.08
55.03
180 170 160 150 140 130 120 110 100 90 80 70 60 50 40 30 20 10 0
OMe
OMe
MeO
N
OMe
N
OMe
OMe
3ac
CDCl3, 100 MHz

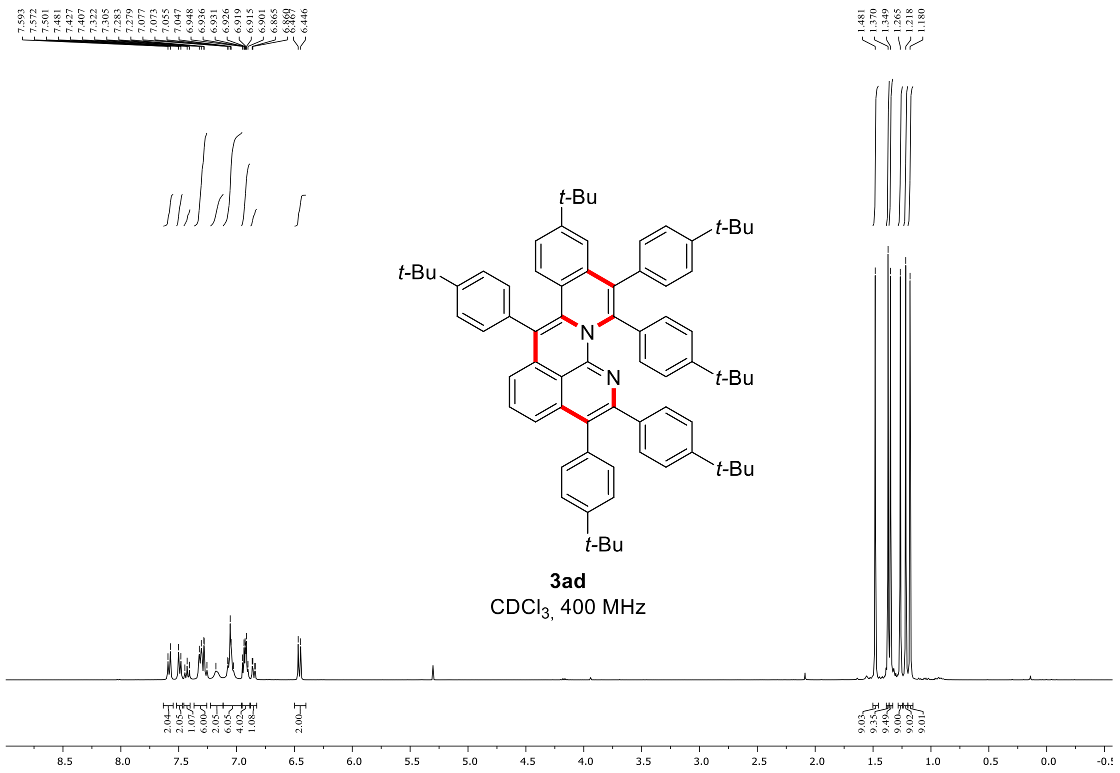
7.593
7.572
7.501
7.481
7.427
7.407
7.322
7.305
7.283
7.279
7.077
7.073
7.055
7.047
6.948
6.936
6.931
6.926
6.919
6.915
6.901
6.865
6.446
1.481
1.370
1.349
1.265
1.218
1.180
t-Bu
N
3ad
CDCl3, 400 MHz
2.04
2.05
1.07
6.00
2.05
6.05
4.02
1.08
2.00
9.03
9.35
9.49
9.00
9.02
9.01
8.5
8.0
7.5
7.0
6.5
6.0
5.5
5.0
4.5
4.0
3.5
3.0
2.5
2.0
1.5
1.0
0.5
0.0

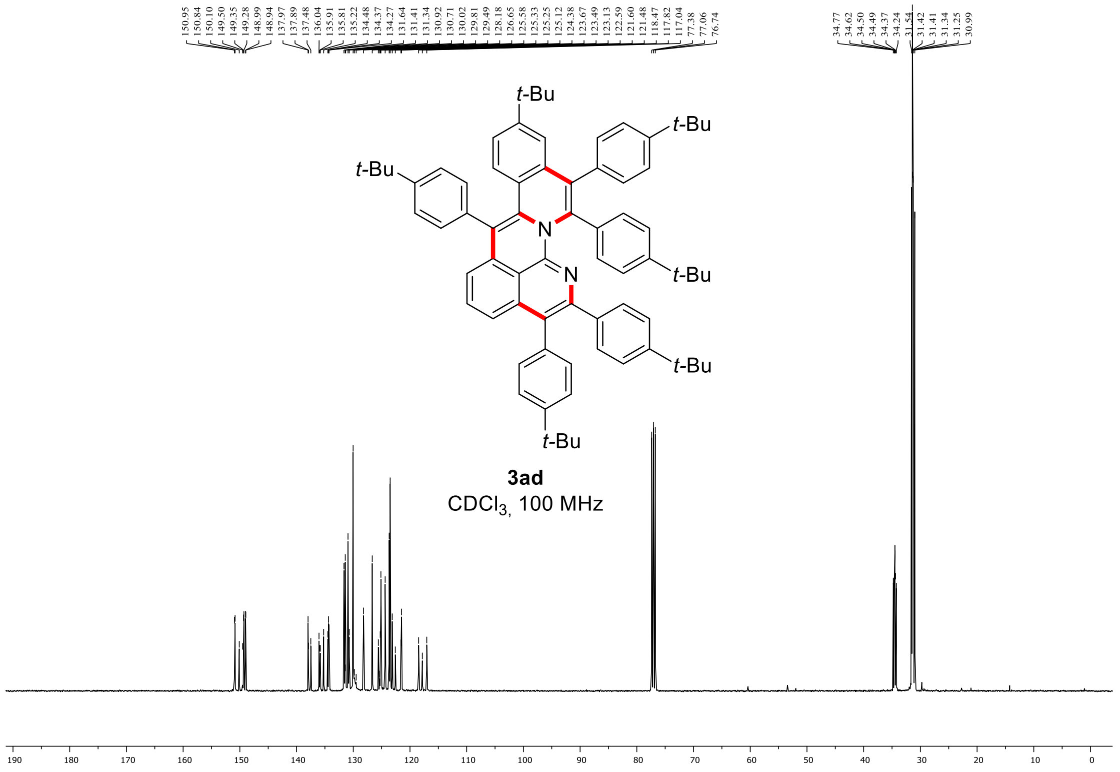
150.95
150.84
150.10
149.50
149.35
149.28
148.99
148.94
137.97
137.89
137.48
136.04
135.91
135.81
135.22
134.48
134.37
134.27
131.64
131.41
131.34
130.92
130.71
130.02
129.81
129.49
128.18
126.65
125.58
125.33
125.25
125.12
124.38
123.67
123.49
123.13
122.59
121.60
121.48
118.47
117.82
117.04
77.38
77.06
76.74
34.77
34.62
34.50
34.49
34.37
34.24
31.54
31.42
31.41
31.34
31.25
30.99
t-Bu
N
3ad
CDCl3, 100 MHz
190
180
170
160
150
140
130
120
110
100
90
80
70
60
50
40
30
20
10
0

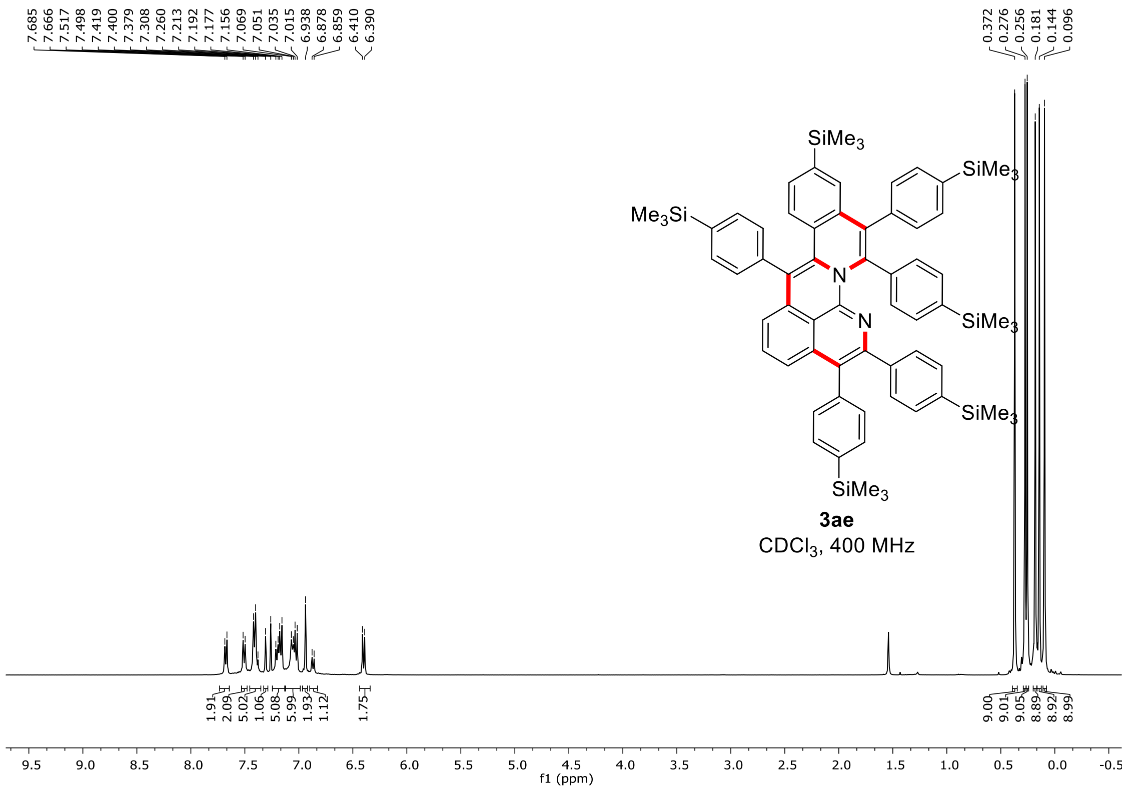

SiMe3
Me3Si
SiMe3
SiMe3
SiMe3
SiMe3
N
N
3ae
CDCl3, 400 MHz
f1 (ppm)

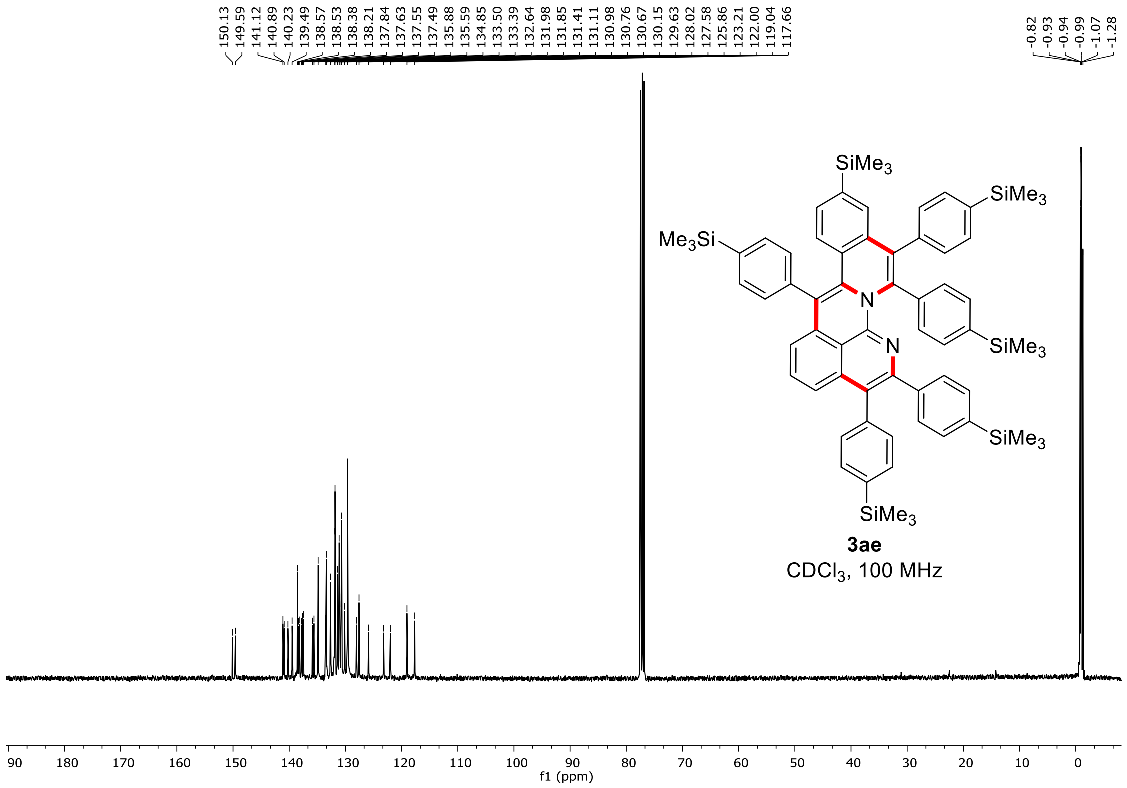

SiMe3
Me3Si
SiMe3
SiMe3
SiMe3
SiMe3
N
N
3ae
CDCl3, 100 MHz
f1 (ppm)

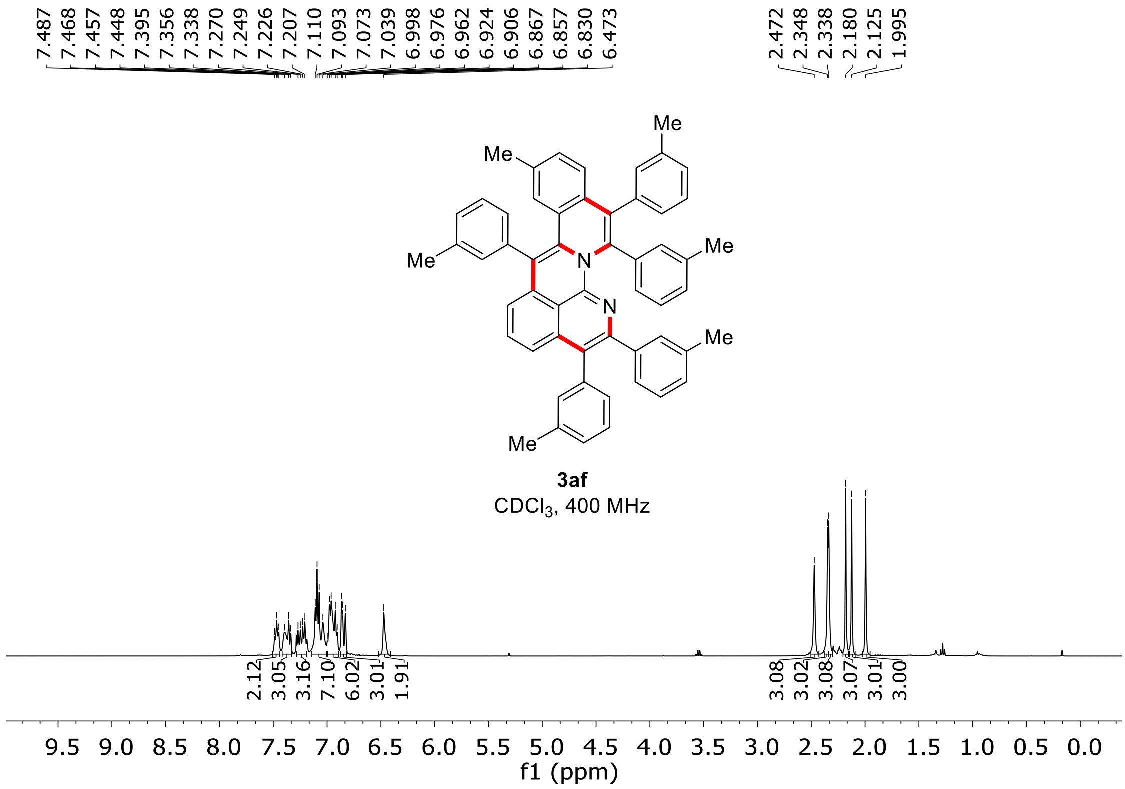
7.487 7.468 7.457 7.448 7.395 7.356 7.338 7.270 7.249 7.226 7.207 7.110 7.093 7.073 7.039 6.998 6.976 6.962 6.924 6.906 6.867 6.857 6.830 6.473
2.472 2.348 2.338 2.180 2.125 1.995
Me
N
3af
CDCl3, 400 MHz
2.12 3.05 3.16 7.10 6.02 3.01 1.91
3.08 3.02 3.08 3.07 3.01 3.00
9.5 9.0 8.5 8.0 7.5 7.0 6.5 6.0 5.5 5.0 4.5 4.0 3.5 3.0 2.5 2.0 1.5 1.0 0.5 0.0
f1 (ppm)

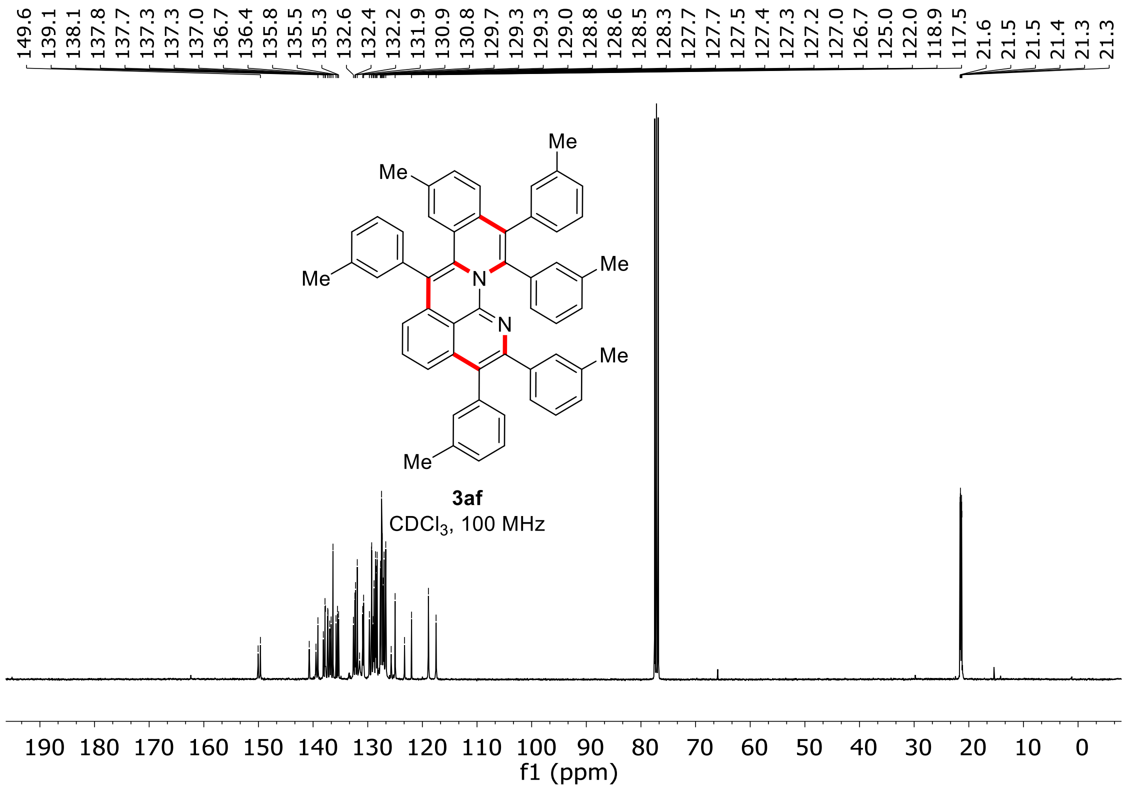
149.6 139.1 138.1 137.8 137.7 137.3 137.3 137.0 136.7 136.4 135.8 135.5 135.3 132.6 132.4 132.2 131.9 130.9 130.8 129.7 129.3 129.3 129.0 128.8 128.6 128.5 128.3 127.7 127.7 127.5 127.4 127.3 127.2 127.0 126.7 125.0 122.0 118.9 117.5 21.6 21.5 21.5 21.4 21.3 21.3
Me
N
3af
CDCl3, 100 MHz
190 180 170 160 150 140 130 120 110 100 90 80 70 60 50 40 30 20 10 0
f1 (ppm)

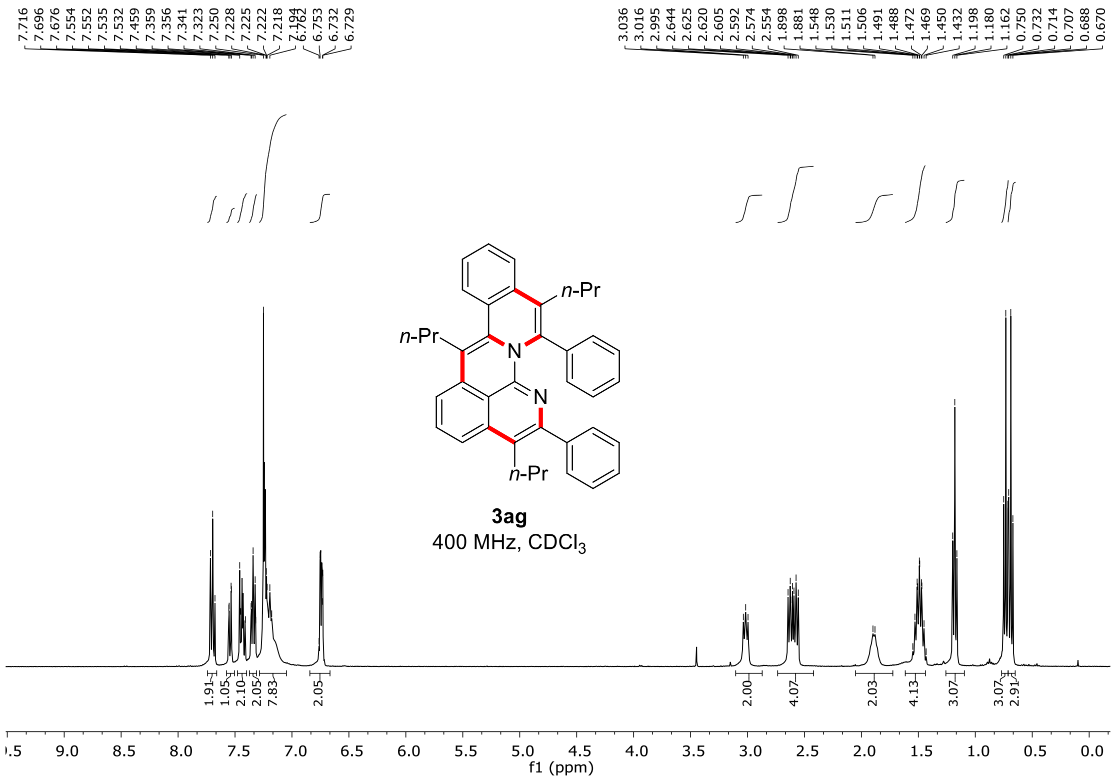
n-Pr
n-Pr
N
N
n-Pr
3ag
400 MHz, CDCl3
f1 (ppm)

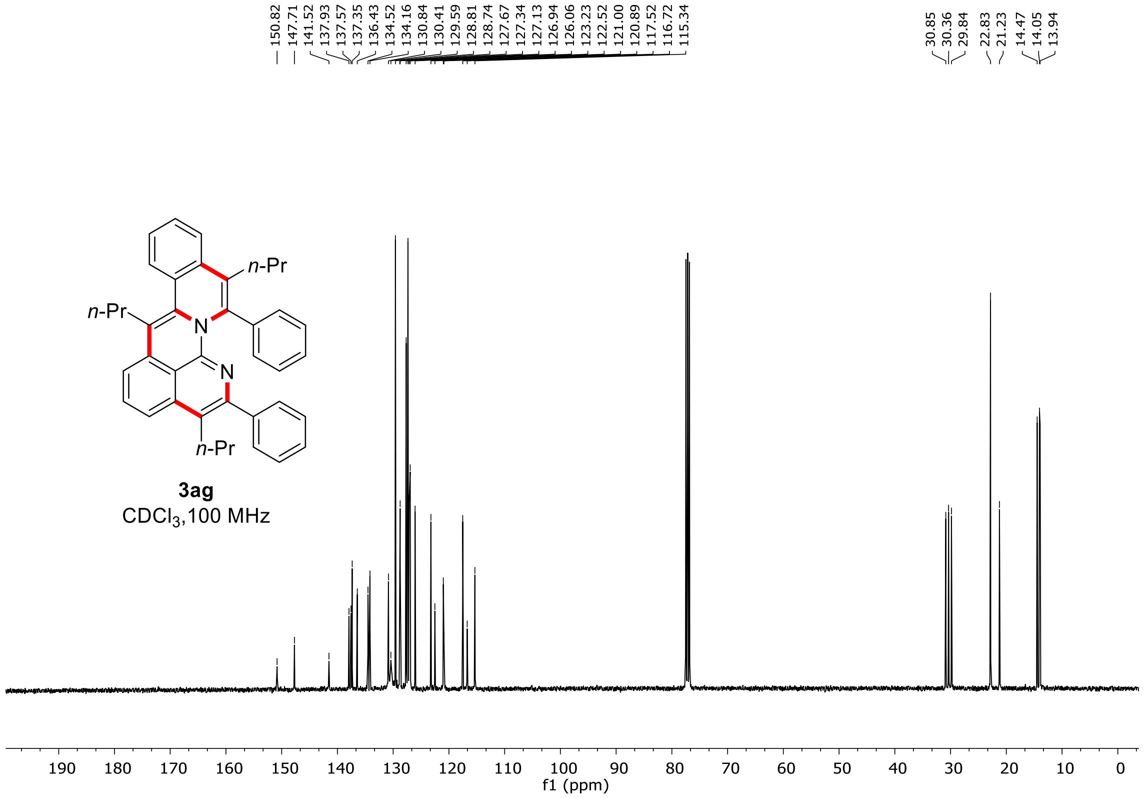
n-Pr
n-Pr
N
N
n-Pr
3ag
CDCl3,100 MHz
f1 (ppm)

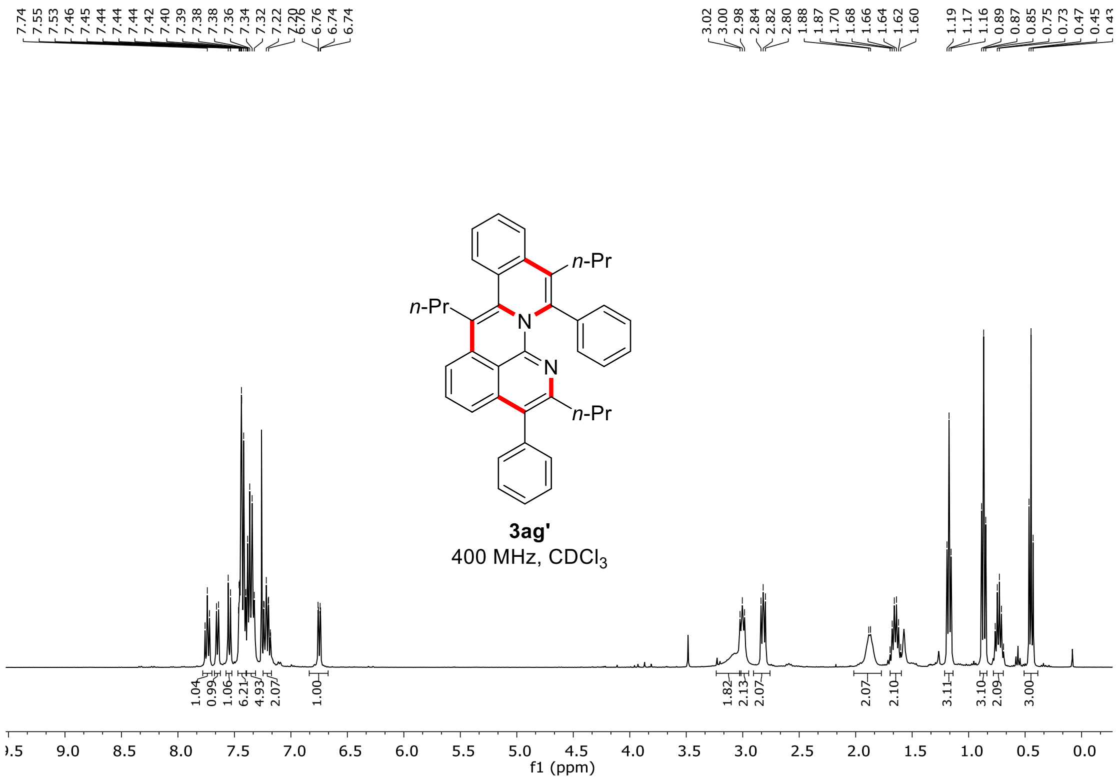
7.74 7.55 7.53 7.46 7.45 7.44 7.44 7.44 7.42 7.40 7.39 7.38 7.38 7.36 7.34 7.32 7.22 7.20 6.76 6.76 6.74 6.74
3.02 3.00 2.98 2.84 2.82 2.80 1.88 1.87 1.70 1.68 1.66 1.64 1.62 1.60 1.19 1.17 1.16 0.89 0.87 0.85 0.75 0.73 0.47 0.45
n-Pr
n-Pr
N
N
n-Pr
3ag'
400 MHz, CDCl3
1.04 0.99 1.06 6.21 4.93 2.07 1.00 1.82 2.13 2.07 2.07 2.10 3.11 3.10 2.09 3.00
f1 (ppm)

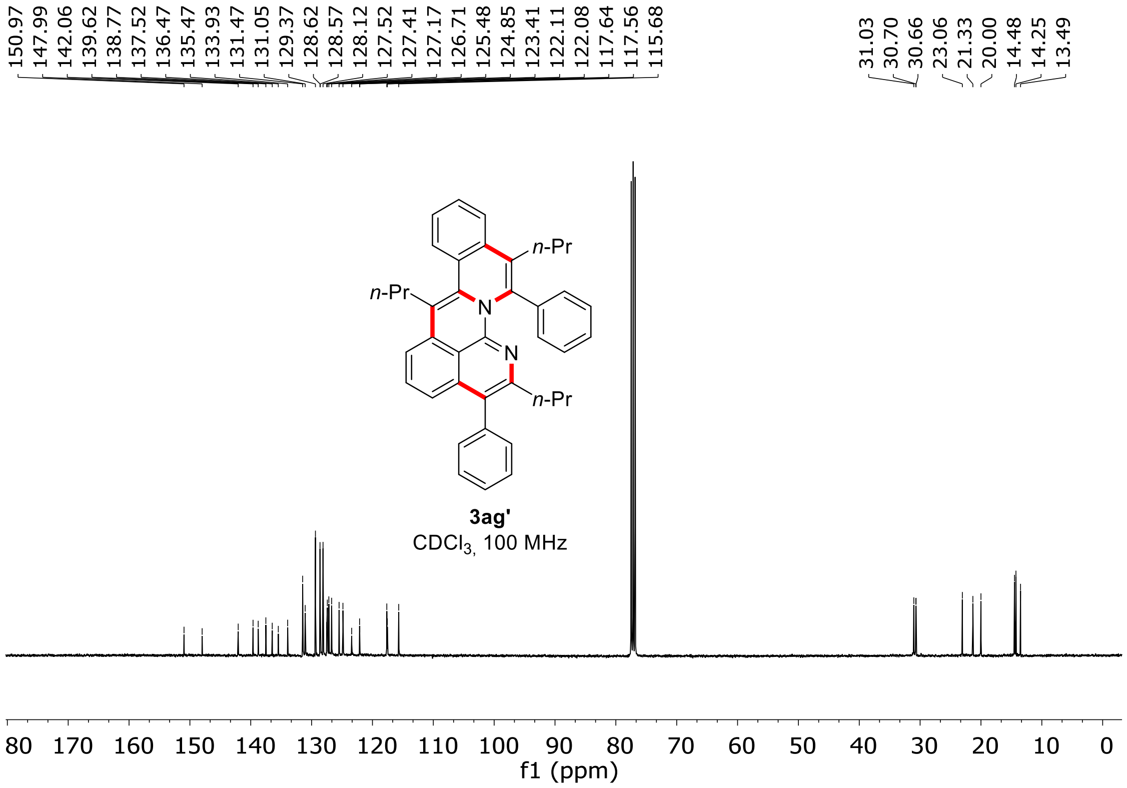
150.97 147.99 142.06 139.62 138.77 137.52 136.47 135.47 133.93 131.47 131.05 129.37 128.62 128.57 128.12 127.52 127.41 127.17 126.71 125.48 124.85 123.41 122.11 122.08 117.64 117.56 115.68
31.03 30.70 30.66 23.06 21.33 20.00 14.48 14.25 13.49
n-Pr
n-Pr
N
N
n-Pr
3ag'
CDCl3, 100 MHz
f1 (ppm)

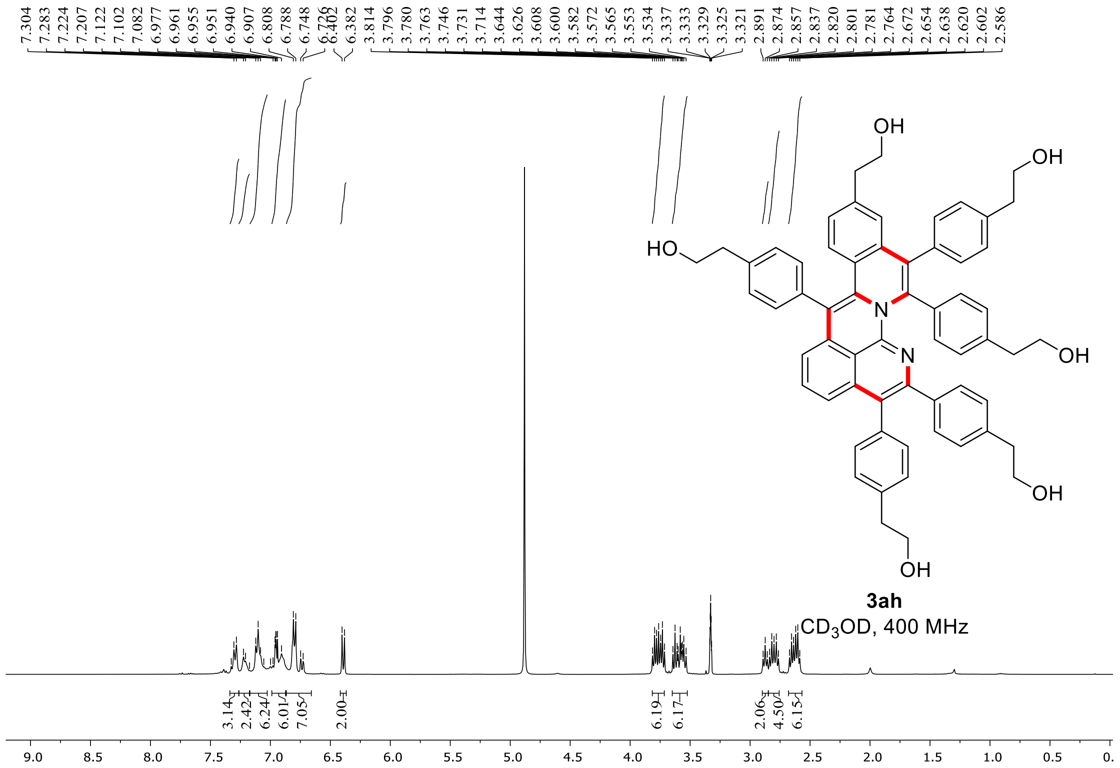
7.304 7.283 7.224 7.207 7.122 7.102 7.082 6.977 6.961 6.955 6.951 6.940 6.907 6.808 6.788 6.748 6.726 6.402 6.382 3.814 3.796 3.780 3.763 3.746 3.731 3.714 3.644 3.626 3.608 3.600 3.582 3.572 3.565 3.553 3.534 3.337 3.333 3.329 3.325 3.321 2.891 2.874 2.857 2.837 2.820 2.801 2.781 2.764 2.672 2.654 2.638 2.620 2.602 2.586
OH
OH
HO
N
N
OH
OH
OH
3ah
CD3OD, 400 MHz
3.14
2.42
6.24
6.01
7.05
2.00
6.19
6.17
2.06
4.50
6.15
9.0 8.5 8.0 7.5 7.0 6.5 6.0 5.5 5.0 4.5 4.0 3.5 3.0 2.5 2.0 1.5 1.0 0.5 0.

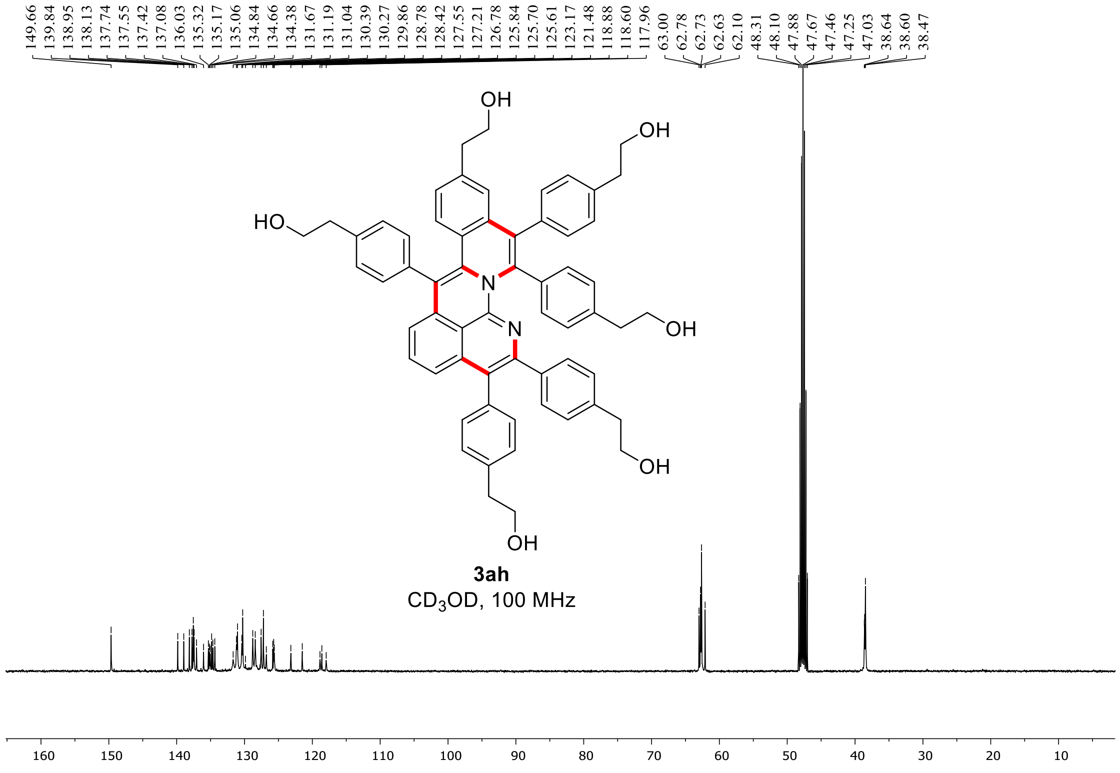
149.66 139.84 138.95 138.13 137.74 137.55 137.42 137.08 136.03 135.32 135.17 135.06 134.84 134.66 134.38 131.67 131.19 131.04 130.39 130.27 129.86 128.78 128.42 127.55 127.21 126.78 125.84 125.70 125.61 123.17 121.48 118.88 118.60 117.96 63.00 62.78 62.73 62.63 62.10 48.31 48.10 47.88 47.67 47.46 47.25 47.03 38.64 38.60 38.47
OH
OH
HO
N
N
OH
OH
OH
3ah
CD3OD, 100 MHz
160 150 140 130 120 110 100 90 80 70 60 50 40 30 20 10

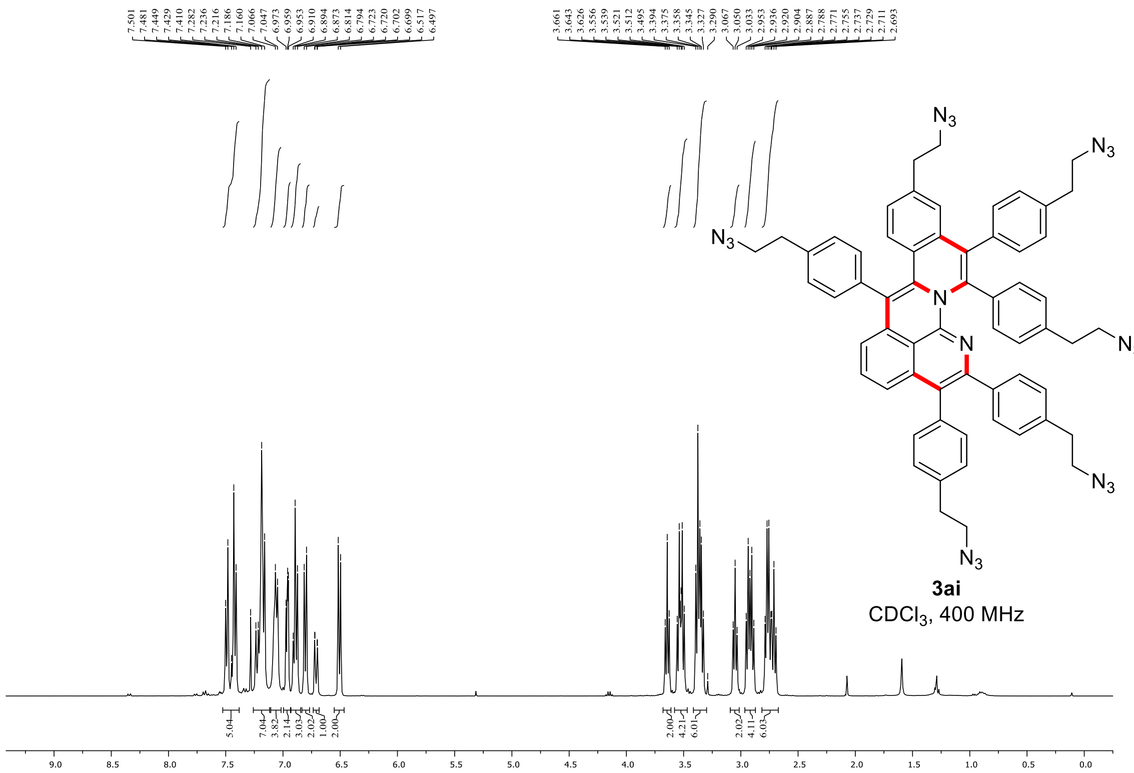
3ai
CDCl3, 400 MHz

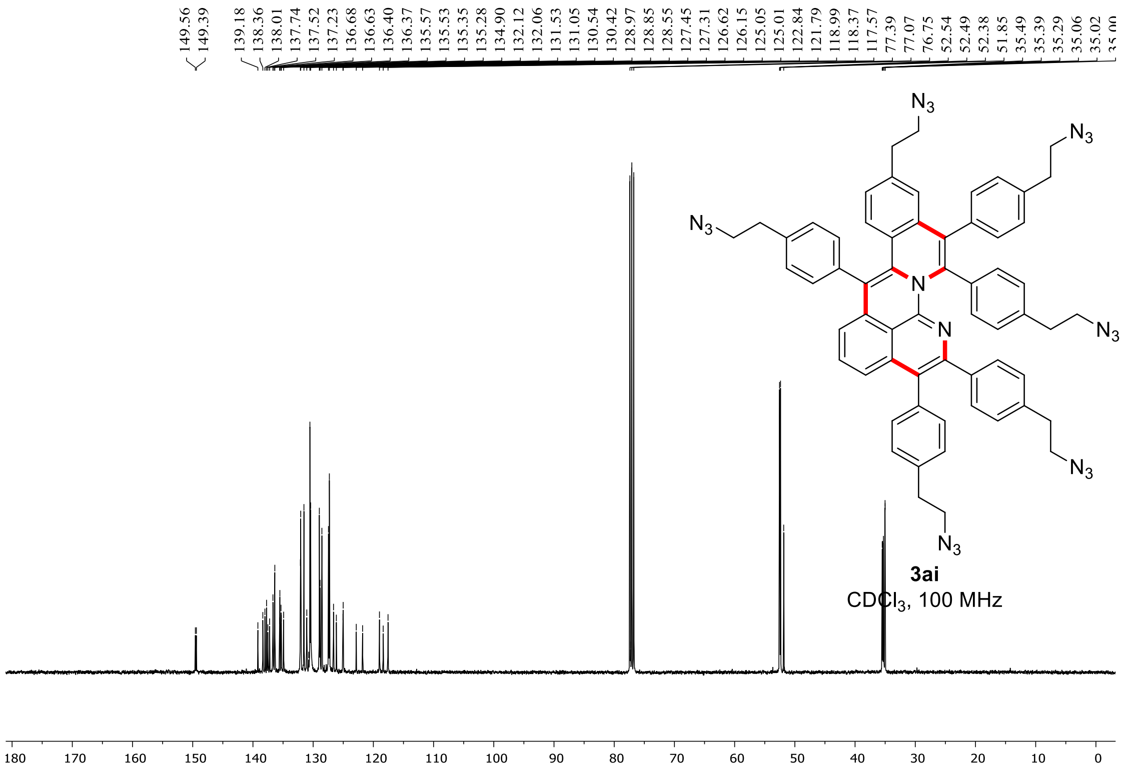
3ai
CDCl3, 100 MHz

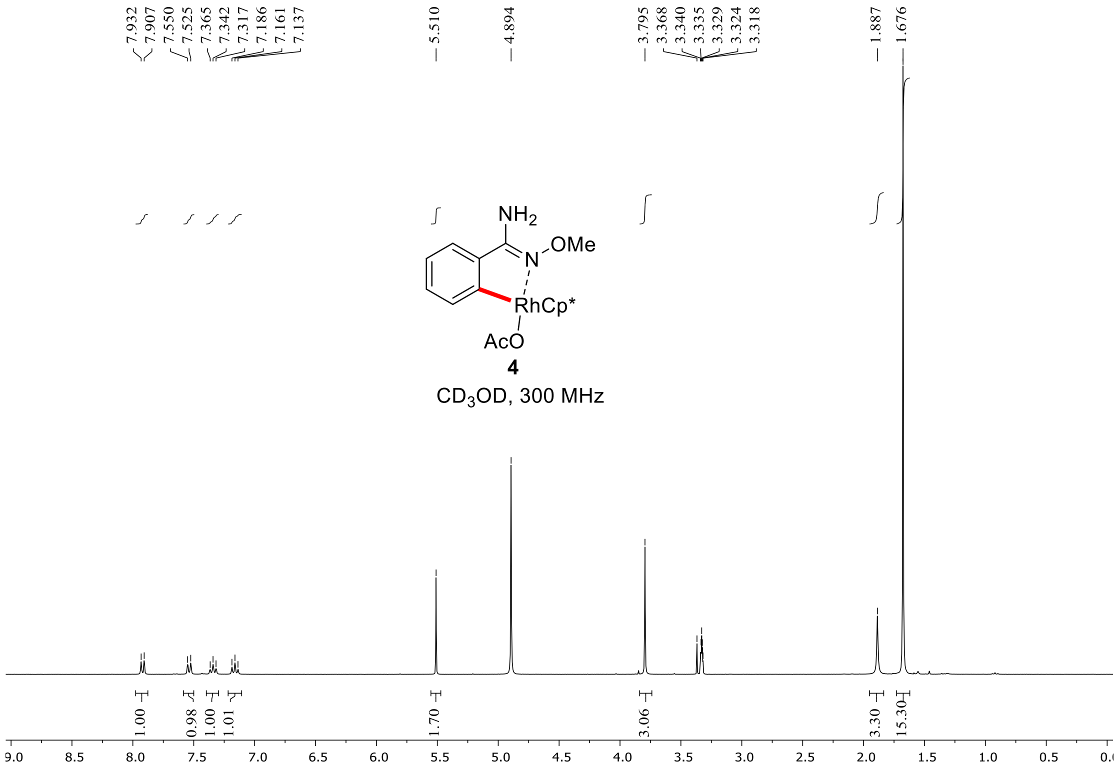

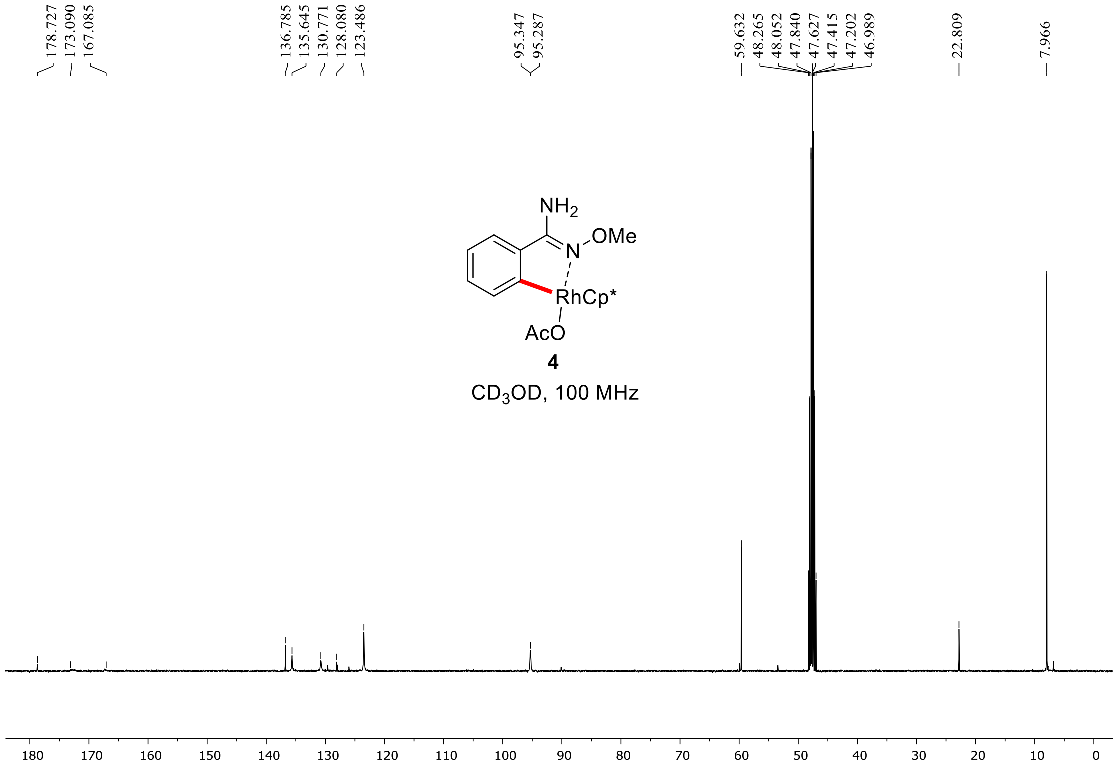

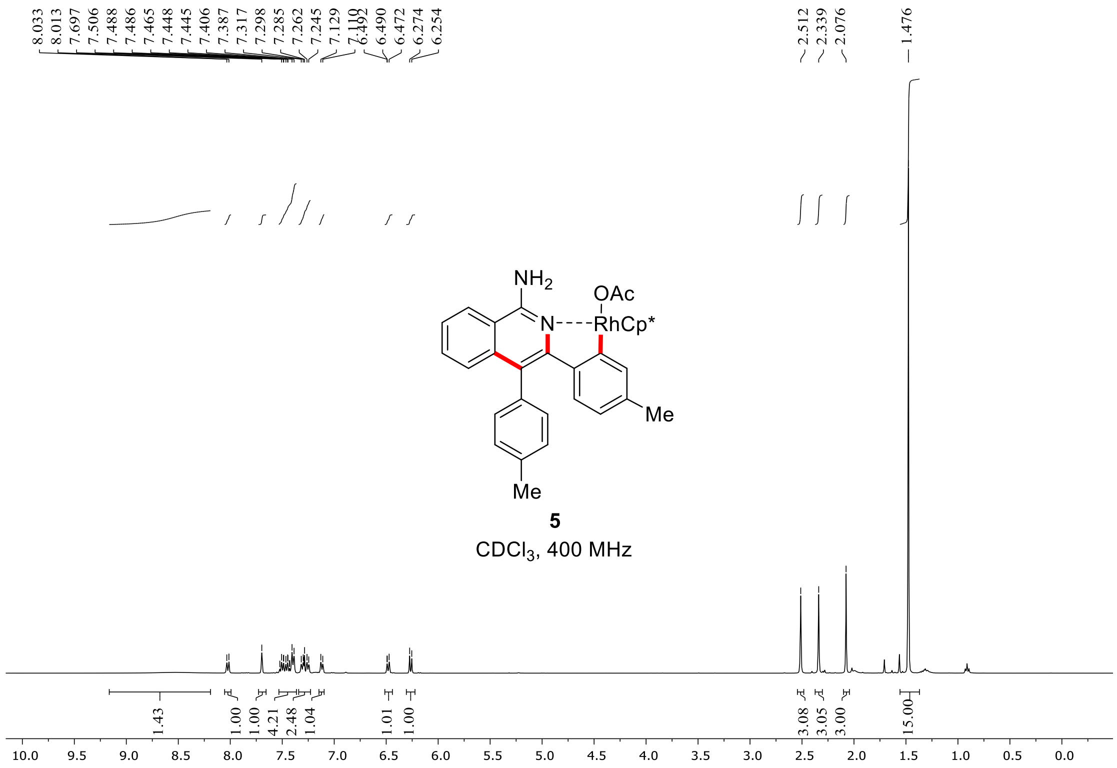
NH2
OAc
N
RhCp*
Me
Me
5
CDCl3, 400 MHz

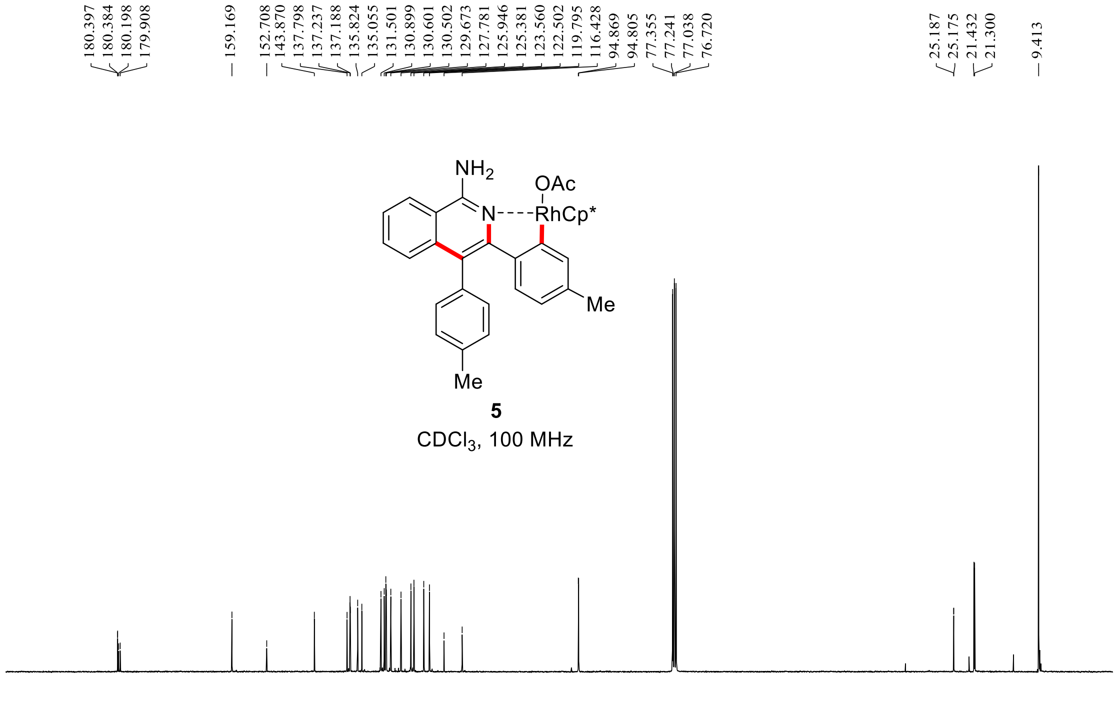
NH2
OAc
N
RhCp*
Me
Me
5
CDCl3, 100 MHz

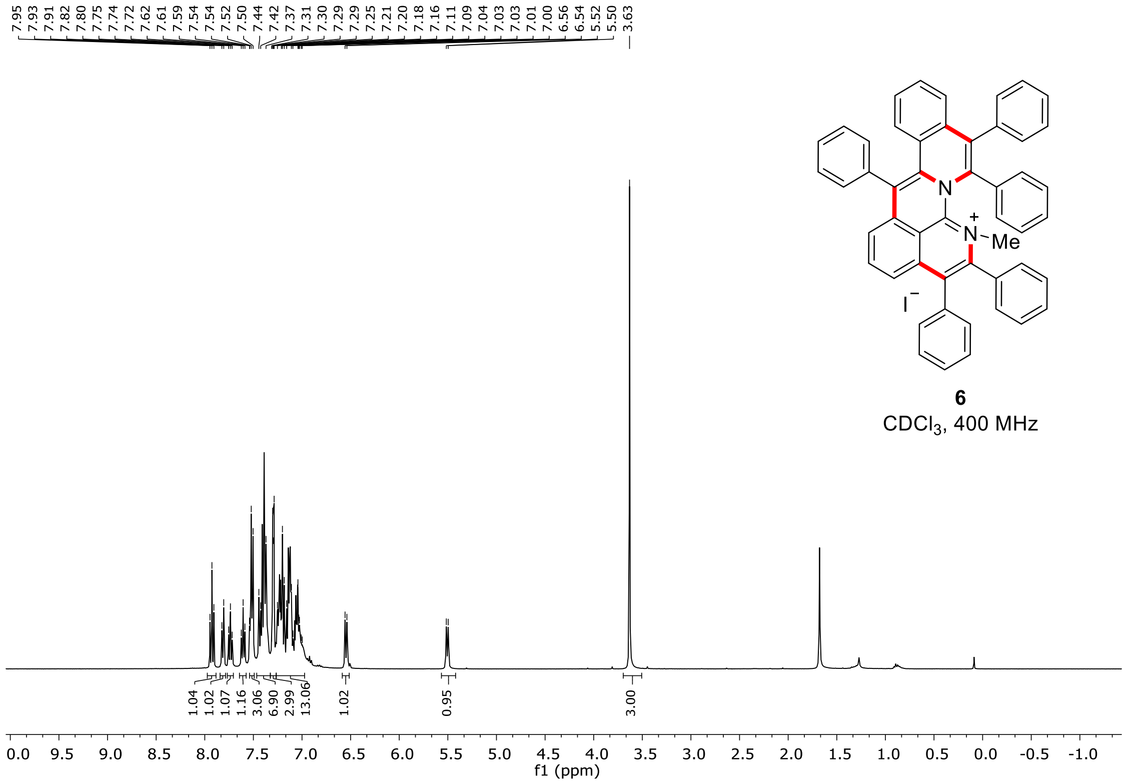
N
N
Me
I
6
CDCl3, 400 MHz
f1 (ppm)

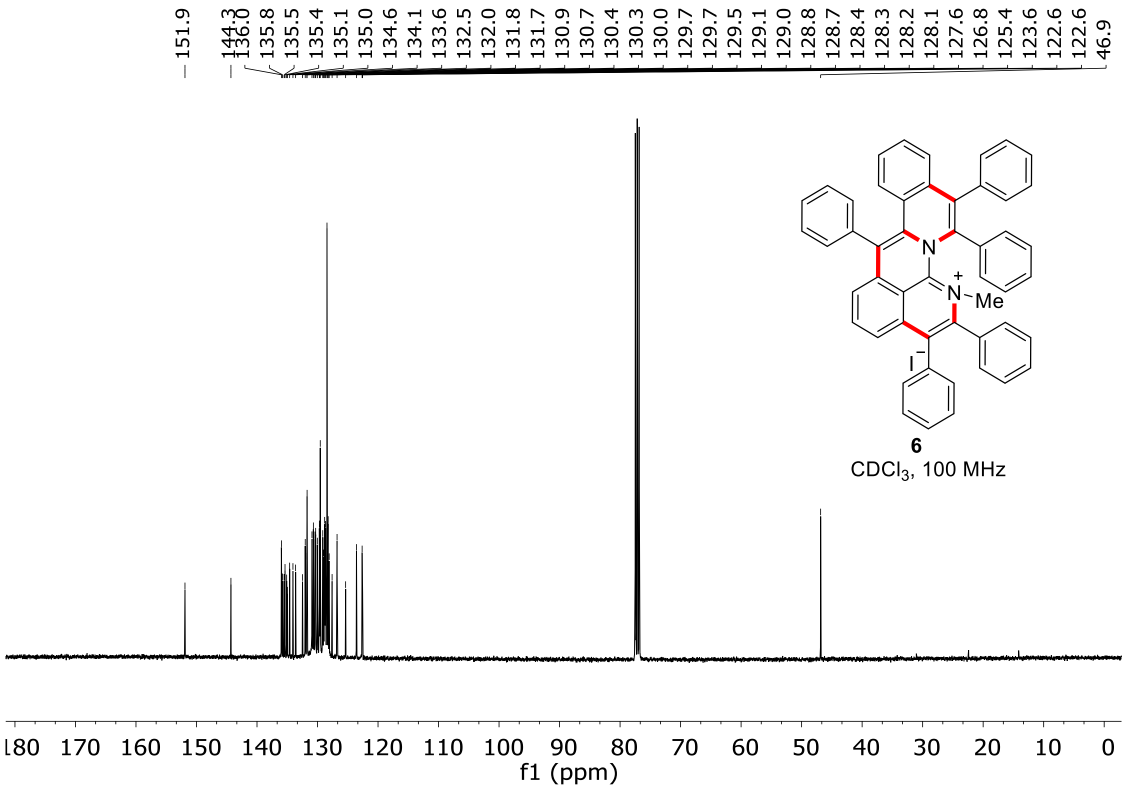
N
N
Me
I
6
CDCl3, 100 MHz
f1 (ppm)

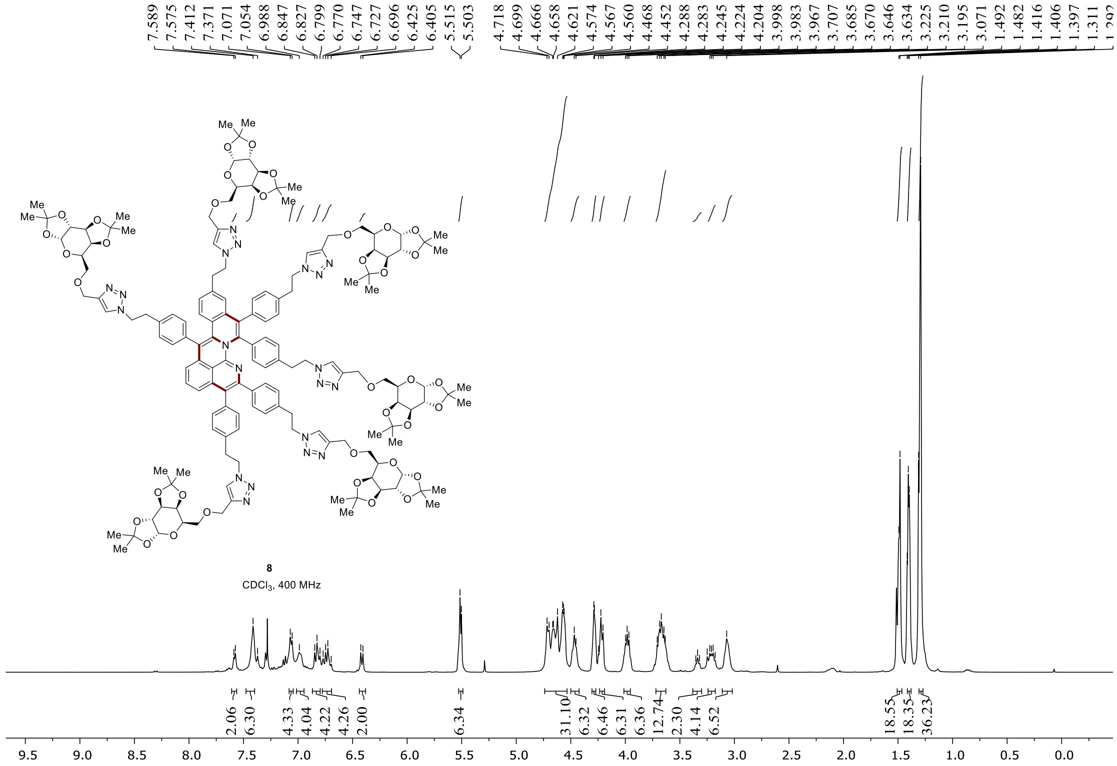
8
CDCl3, 400 MHz

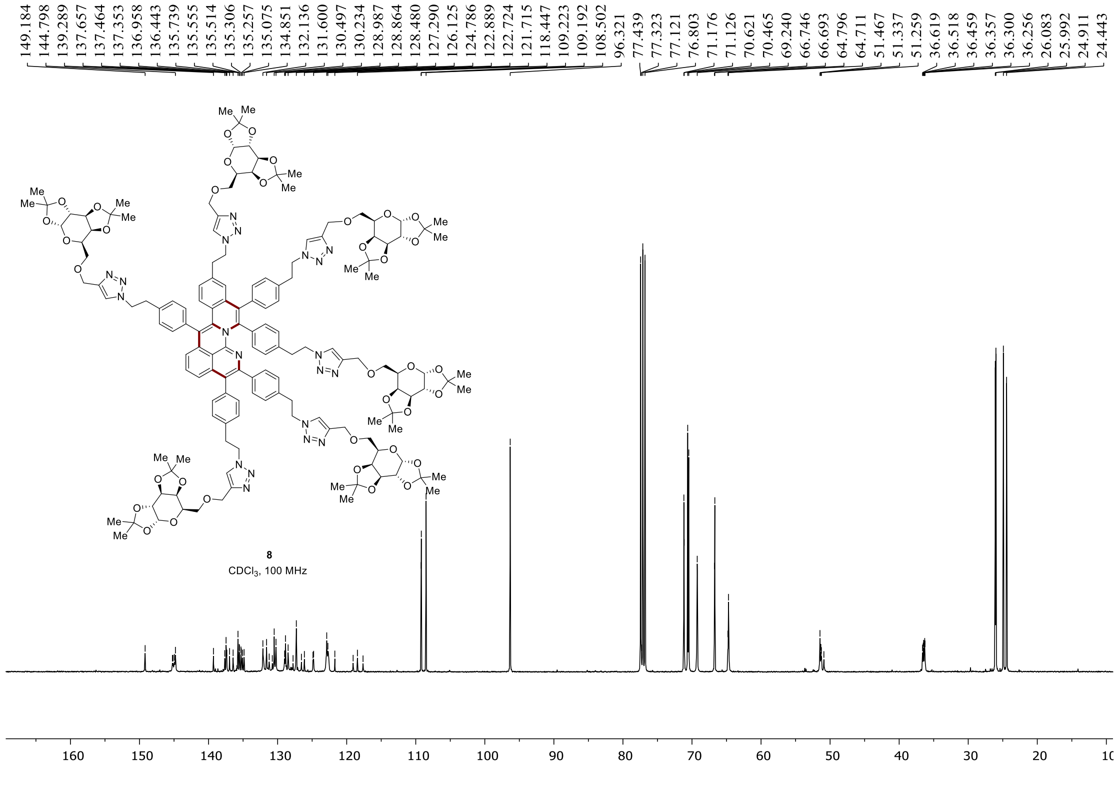
8
CDCl3, 100 MHz
